# Tumor intrinsic immunity related proteins may be novel tumor suppressors in some types of cancer

**DOI:** 10.1038/s41598-019-47382-3

**Published:** 2019-07-29

**Authors:** Donghai Xiong, Yian Wang, Ming You

**Affiliations:** Center for Disease Prevention Research and Department of Pharmacology and Toxicology, Medical College of Wisconsin, 8701 Watertown Plank Road, Milwaukee, WI 53226 USA

**Keywords:** Cancer genetics, Cancer

## Abstract

Immune checkpoint blockade therapy (ICBT) can unleash T-cell responses against cancer. However, only a small fraction of patients exhibited responses to ICBT. The role of immune checkpoints in cancer cells is not well understood. In this study, we analyzed T-cell coinhibitory/costimulatory genes across more than 1100 samples of the Cancer Cell Line Encyclopedia (CCLE). Nearly 90% of such genes were not expressed or had low expression across the CCLE cancer cell lines. Cell line screening showed the enrichment of cancer cells deprived of the expression of *CD27*, *CEACAM1*, *CTLA4*, *LRIG1*, *PDCD1LG2*, or *TNFRSF18*, suggesting their role as tumor suppressor. The metagene expression signature derived from these six genes - Immu6Metagene was associated with prolonged survival phenotypes. A common set of five oncogenic pathways were significantly inhibited in different types of tumors of the cancer patients with good survival outcome and high Immu6Metagene signature expression. These pathways were TGF-β signaling, angiogenesis, EMT, hypoxia and mitotic process. Our study showed that oncoimmunology related molecules especially the six genes of the Immu6Metagene signature may play the tumor suppressor role in certain cancers. Therefore, the ICBT targeting them should be considered in such context to improve the efficacy.

## Introduction

Immune checkpoint blockade therapies (ICBT) have demonstrated significant therapeutic promise. However, the success of ICBT is limited to a small number of cancer types and a small proportion of the cancer patients. Recent studies showed that some patients even developed hyperprogressive diseases (HPD) with accelerated tumor growth after ICBT^1–11^. T-cell coinhibitory/costimulatory molecules are the major targets of ICBT, which are now being investigated intensively for their therapeutic potentials. However, the current knowledge of the fundamental biological roles of these molecules is very limited and unsatisfactory. The previous efforts had largely focused on CTLA4 and PD-1 immune checkpoint blockades while the other T-cell coinhibitory/costimulatory molecules had not been well understood in terms of their roles in cancer biology. Combinatorial therapies involving more than one immunotherapy are actively being tested with the hope of significantly improving the efficacy and achieving a wide breadth of treating different types of tumors. However, the progress is not strikingly impressive due to the many unknown aspects of these molecules in cancer etiology.

The basic research of immunotherapy has provided a list of T-cell immunity related immune checkpoints that are the important targets of ICBT. According to the generally accepted concept in the field^12–15^, immune checkpoints are the molecules that either promote or inhibit T-cell activation. The therapeutic potential of them is now being investigated preclinically and clinically. Paradoxically, in addition to their role of promoting or inhibiting T-cell based immunity, the checkpoint proteins may play the role of ‘tumor suppressor’ in cancer cells^16,17^. It is easy to understand that the T cell immunity co-stimulatory genes may act as tumor suppressors because the stimulation of T cell activity by such genes can enhance the anti-tumor immunity of the tumor microenvironment (TME). On the other hand, it may be hard to understand why the T cell immunity co-inhibitory genes could act as tumor suppressors. Regardless of their role as either co-stimulatory or inhibitory in immune cells, these genes may also play the tumor suppressor role in cancer cells *per se*. For example, PD-1 is the most well-known T cell immunity co-inhibitory gene. However, recent studies revealed its role as tumor suppressor^16,17^. The underlying biological mechanisms may include multiple possibilities, such as 1) PD-1 activation increases cancer cell apoptosis through upregulation of pro-apoptotic proteins such as BIM; 2) PD-1 activation impedes cancer cell cycle progression at the G1-S checkpoint through multiple complex mechanisms, including the upregulation of the G1 phase inhibitor p15INK4 and indirectly increasing inhibition of cyclin-dependent kinase 2^16,17^. Therefore, both co-stimulatory and inhibitory genes may act as tumor suppressor genes depending on the context and their role in cancer cells. These researches suggested that more comprehensive analysis of the oncoimmunology related genes should be performed, which may reveal the unexpected novel roles of these genes such as the tumor suppressor functions in certain types of cancer.

The Cancer Cell Line Encyclopedia (CCLE, http://www.broadinstitute.org/ccle/home) is a compiled public resource that contains mutation, gene expression and massively parallel sequencing data from more than 1000 cancer cell lines^18^. We utilized the genomic data from CCLE to analyze the mutational and gene expression status of the 31 prominent genes the encode the T-cell coinhibitory or costimulatory immune checkpoint molecules. These candidate genes were selected based on the results of multiple outstanding reviews^12–15^ that provide a comprehensive list of T-cell immunity related checkpoints. For the validation of the tumor suppressor role of the candidate genes, we also analyzed the RNAi screening data from the cancer dependency map project^19^, TCGA (The Cancer Genome Atlas)^20^ clinical and gene expression data from the Broad Institute TCGA GDAC Firehose project (https://gdac.broadinstitute.org/), as well as gene expression data from distinct microarray studies. Our results showed that a subset of the cancer immunity related molecules could play the tumor suppressor role so the ICBT targeting them should be taken with caution.

## Results

### Overall mutation and expression pattern of the prominent immune coinhibitory/costimulatory genes in cancer cell lines

**Figure 1:**
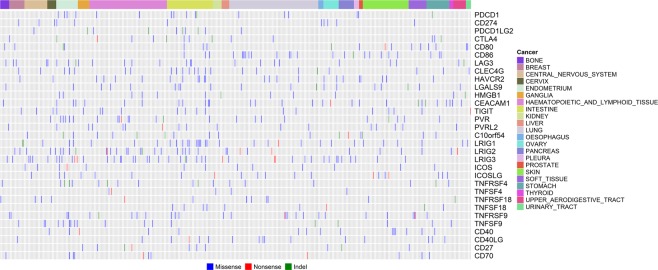
Overall profiling of the Nonsilent somatic mutations of the immune checkpoint genes in the CCLE cancer cell lines. The 370 CCLE cancer cell lines from 22 tissues had significant number of nonsilent somatic mutations in the 31 immune checkpoint genes.

We compiled a list of 31 prominent immune coinhibitory/costimulatory genes (Table 1) according to the recent progress in the field^13^. Focusing on these genes, firstly we analyzed the cancer cell line mutation data from the CCLE project. After filtering germline mutations and SNPs archived in the public databases, it was found that 370 out of the 1509 CCLE cell lines originating from the 22 tissues had a significant number of nonsilent somatic mutations (including nonsynonymous [missense] mutations, nonsense [stopgain] mutations, insertions and deletions [indels]) in the set of 31 prominent immune coinhibitory/costimulatory genes (Fig. 1). The rate of nonsilent somatic mutations within the immune coinhibitory/costimulatory genes in the cancer cell lines is therefore estimated to be around 24.5% (370/1509). The gene expression data of the 1103 CCLE cell lines from the 22 tissues were also downloaded and analyzed. Among the 1103 cell lines with expression data, 27 cell lines did not have mutation data available, 799 cell lines had mutation data but did not contain nonsilent mutations in any of the 31 prominent immune coinhibitory/costimulatory genes, and 277 cell lines did have nonsilent mutations in at least one of the 31 genes (Fig. S1). The expression of each of the 31 genes did not change significantly across the 1103 CCLE cancer cell lines stratified by the overall mutation status of the set of 31 immune coinhibitory/costimulatory genes (Fig. S2, mutation group vs non-mutation group, adjusted P value > 0.1 for each of the 31 genes).

**Table 1:** Information of the genes encoding the 31 T-cell coinhibitory/costimulatory molecules that were investigated in this study.

Symbol	Entrez Gene Name	Type(s)
CTLA4	cytotoxic T-lymphocyte associated protein 4	transmembrane receptor
CD80 (B7-1)	CD80 molecule	transmembrane receptor
CD86 (B7-2)	CD86 molecule	transmembrane receptor
PDCD1 (PD-1)	programmed cell death 1	phosphatase
CD274 (PD-L1)	CD274 molecule	enzyme
PDCD1LG2 (PD-L2)	programmed cell death 1 ligand 2	enzyme
LAG3	lymphocyte activating 3	transmembrane receptor
CLEC4G (LSECtin)	C-type lectin domain family 4 member G	other
HAVCR2 (TIM3)	hepatitis A virus cellular receptor 2	other
LGALS9	galectin 9	other
HMGB1	high mobility group box 1	transcription regulator
CEACAM1	carcinoembryonic antigen related cell adhesion molecule 1	transporter
TIGIT	T cell immunoreceptor with Ig and ITIM domains	other
PVR (CD155)	poliovirus receptor	other
PVRL2 (NECTIN2, CD112)	nectin cell adhesion molecule 2	transmembrane receptor
VISTA (VSIR, C10orf54)	V-set immunoregulatory receptor	other
LRIG1	leucine rich repeats and immunoglobulin like domains 1	other
LRIG2	leucine rich repeats and immunoglobulin like domains 2	other
LRIG3	leucine rich repeats and immunoglobulin like domains 3	other
ICOS	inducible T cell costimulator	transmembrane receptor
ICOSLG	inducible T cell costimulator ligand	other
OX40 (TNFRSF4)	TNF receptor superfamily member 4	transmembrane receptor
OX40L (TNFSF4)	TNF superfamily member 4	cytokine
GITR (TNFRSF18)	TNF receptor superfamily member 18	transmembrane receptor
GITRL (TNFSF18)	TNF superfamily member 18	cytokine
4-1BB (TNFRSF9)	TNF receptor superfamily member 9	transmembrane receptor
4-1BBL (TNFSF9)	TNF superfamily member 9	cytokine
CD40 (TNFRSF5)	CD40 molecule	transmembrane receptor
CD40L (CD40LG, TNFSF5)	CD40 ligand	cytokine
CD27 (TNFRSF7)	CD27 molecule	transmembrane receptor
CD70 (TNFSF7)	CD70 molecule	cytokine

### Potential tumor suppressor role of immune coinhibitory/costimulatory genes indicated by no/low expression in most of the cancer cell lines and downregulation in the tumors across different TCGA cancer types

We examined the expression of the immune coinhibitory/costimulatory genes in the 1103 CCLE cell lines stratified by their tissue origins (from a total of 22 tissue sources). According to the previous established criteria by the “Expression Atlas” resource (https://www.ebi.ac.uk/gxa/FAQ.html), we found that none of the 31 immune coinhibitory/costimulatory genes was highly expressed in any of the CCLE cell lines. These genes were classified into three groups based on the expression status: A) “No expression” group, i.e., not expressed across almost all the CCLE cancer cell lines, involving 6 genes - *CD40LG*, *CD80*, *CLEC4G*, *TIGIT*, *TNFRSF4*, and *ICOS* (Fig. S3); B) “Low expression” group, i.e., not expressed or having low expression in the majority of cancer cell lines across the 22 distinct types of tissues, involving 22 genes – *C10orf54* (*VISTA*), *CD27*, *CD40*, *CD86*, *CD274* (PD-*L1*), *CEACAM1*, *CTLA4*, *HAVCR2* (TIM3), *CD70*, *ICOSLG*, *LAG3*, *LGALS9*, *LRIG1*, *LRIG2*, *LRIG3*, *PDCD1* (*PD-1*), *PDCD1LG2* (*PD-L2*), *TNFRSF9*, *TNFRSF18*, *TNFSF4*, *TNFSF9*, and *TNFSF18* (Fig. S4); C) “Medium expression” group, i.e., having medium expression in the majority of cancer cell lines across the 22 distinct types of tissues, involving 3 genes – *HMGB1*, *PVR*, and *PVRL2* (*NECTIN2*) (Fig. S5).

We further examined the differential expression of the 28 genes from the above Group A or B between tumor samples and normal tissues across the different TCGA cancer types using the GEPIA program^21^, which used the unpaired t-test for comparison of tumor and normal tissue expression data. The tumor samples had detectable expression of these genes due to the contamination by normal cells and the presence of immune cells infiltrating the tumors such as TILs (Tumor-infiltrating lymphocytes) and stromal cells. However, even under the “impurity tumor” circumstances, these 28 genes were still downregulated in the tumor samples compared to the normal tissues across many TCGA cancer types (Table 2, Fig. S6). In summary, we observed either no expression or extremely low expression of the 28 genes from Group A and B in the CCLE cancer cell lines and the downregulation of them in the tumor samples relative to the normal tissues across different TCGA cancer types.

**Table 2:** The expressions of the 28 genes from no/low expression groups in cancer cell lines were downregulated in the tumor samples compared to the normal tissues across different TCGA cancer types, which are shown in this table and in Fig. S6.

Gene	Downregulated in TCGA tumors vs normal tissues
CTLA4	KICH, PAAD, THYM
CD80 (B7-1)	LUAD, LUSC, PAAD, THCA, THYM
CD86 (B7-2)	COAD, LUAD, LUSC, PAAD, PCPG, READ
PDCD1 (PD-1)	BLCA, KICH, PAAD, READ, THCA, THYM
CD274 (PD-L1)	LUAD, LUSC, PAAD, PRAD, UCEC
PDCD1LG2 (PD-L2)	BRCA, COAD, KICH, KIRP, LIHC, LUAD, LUSC, PAAD, READ, UCEC
LAG3	COAD, KICH, PAAD, PRAD, READ, UCEC
CLEC4G (LSECtin)	BLCA, BRCA, CESC, CHOL, COAD, LIHC, PAAD, PCPG, READ, STAD, THYM, UCEC
HAVCR2 (TIM3)	LUAD, LUSC, PAAD, PCPG, READ
LGALS9	COAD, LUSC, PAAD, READ
CEACAM1	COAD, HNSC, KICH, KIRC, KIRP, PCPG, PRAD, READ, SARC, THYM
TIGIT	PAAD, READ, THYM
VISTA (VSIR, C10orf54)	BLCA, BRCA, CESC, COAD, KICH, LUAD, LUSC, PRAD, READ, STAD, THYM, UCEC
LRIG1	BLCA, BRCA, CESC, ESCA, HNSC, LUAD, LUSC, READ, THCA, THYM
LRIG2	BRCA, KICH, KIRP, THCA, THYM, UCEC
LRIG3	BLCA, BRCA, KICH, READ, THCA, UCEC
ICOS	LUAD, LUSC, PAAD, THYM
ICOSLG	BLCA, HNSC, KICH, KIRC, KIRP, LUSC, PAAD, PCPG, THYM
OX40 (TNFRSF4)	PAAD, THYM
OX40L (TNFSF4)	KICH, KIRP, PAAD
GITR (TNFRSF18)	COAD, PAAD, THYM
GITRL (TNFSF18)	KICH, PAAD
4-1BB (TNFRSF9)	KICH, PAAD, THYM
4-1BBL (TNFSF9)	BLCA, KICH, PCPG, THYM
CD40 (TNFRSF5)	BRCA, COAD, LUAD, LUSC, PAAD, PRAD, READ, UCEC
CD40L (CD40LG, TNFSF5)	BLCA, BRCA, COAD, HNSC, KICH, LUAD, LUSC, PAAD, READ, THCA, THYM, UCEC
CD27 (TNFRSF7)	COAD, PAAD, READ, THCA, THYM
CD70 (TNFSF7)	KICH, THYM

### Loss-of-function RNAi screening experiments and survival analysis further supported six immune coinhibitory/costimulatory genes’ roles as tumor suppressors

**Figure 2:**
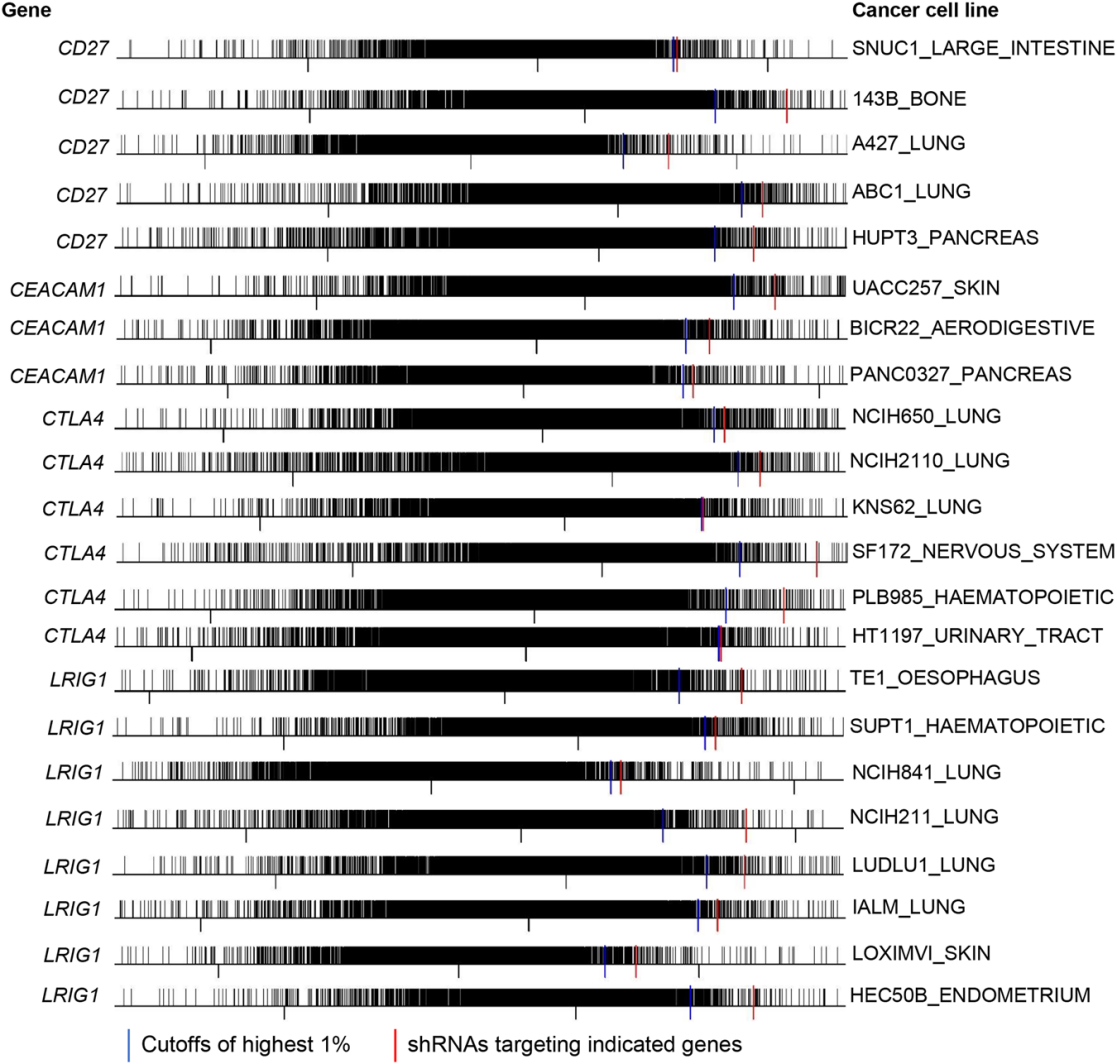
*In vitro* RNAi screening experiments suggested six genes - *CD27*, *CEACAM1*, *CTLA4*, *LRIG1*, *PDCD1LG2*, and *TNFRSF18* as tumor suppressors. Frequency histogram analysis showed that the shRNAs (short hairpin RNAs) targeting one of the six genes were ranked within the top 1% highly expressed shRNAs from the genome-scale library of ∼100,000 pooled shRNAs targeting about 17,080 genes. Plots of the first 22 cell lines for the genes - *CD27*, *CEACAM1*, *CTLA4*, and *LRIG1* were shown.

**Figure 3:**
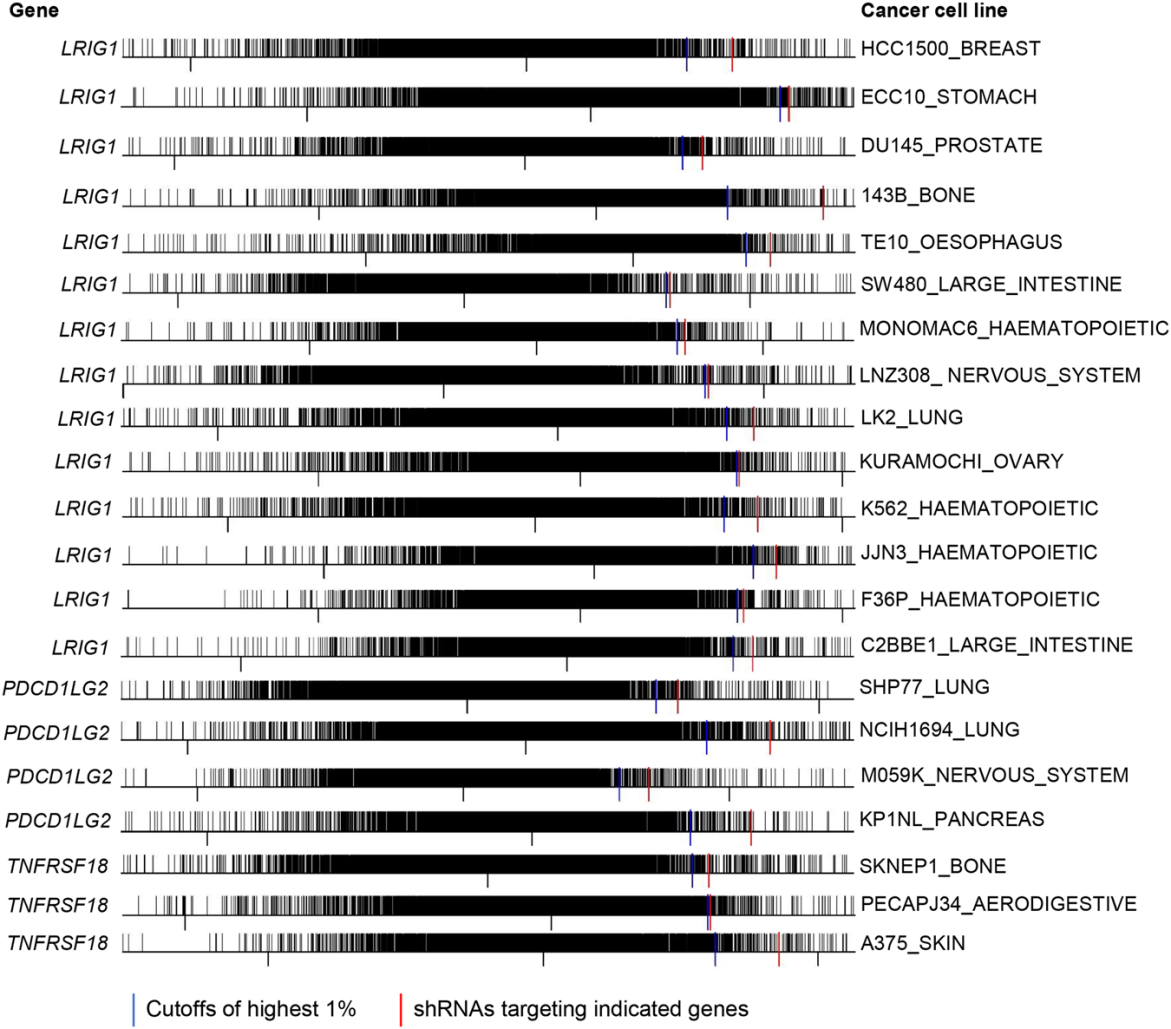
*In vitro* RNAi screening experiments suggested six genes - *CD27*, *CEACAM1*, *CTLA4*, *LRIG1*, *PDCD1LG2*, and *TNFRSF18* as tumor suppressors. Frequency histogram analysis showed that the shRNAs (short hairpin RNAs) targeting one of the six genes were ranked within the top 1% highly expressed shRNAs from the genome-scale library of ∼100,000 pooled shRNAs targeting about 17,080 genes. Plots of the last 21 cell lines for the genes - *LRIG1*, *PDCD1LG2* (*PD-L2*), and *TNFRSF18* were shown.

The functional impact of knocking down the 31 immune coinhibitory/costimulatory genes (Table 1) on tumor survival was further analyzed by using the RNAi screening data from the cancer dependency map project that performed genome-wide scale loss-of-function screens in diverse human cancer cell lines^19^. The results calculated by the DEMETER computational model were used for the analysis of the 31 immune coinhibitory/costimulatory genes. As for the DEMETER results^19^, the high dependency scores meant that the corresponding genes were not essential to cancer cell proliferation and the top ranked scores indicated the corresponding genes may function as tumor suppressors because of the enrichment of the cancer cells expressing their targeting small hairpin RNAs (shRNAs) that knocked down such genes^19,22^. The analysis of RNAi data found that six genes - *CD27*, *CEACAM1*, *CTLA4*, *LRIG1*, *PDCD1LG2* (*PD-L2*), and *TNFRSF18* had the dependency scores ranking within the highest 1% of the 17,080 genes targeted by pooled RNAi screening in 43 cancer cell lines (Figs 2 and 3), suggesting the enrichment of the cancer cells deprived of the corresponding gene expression and thus supporting their role as tumor suppressor.

**Figure 4:**
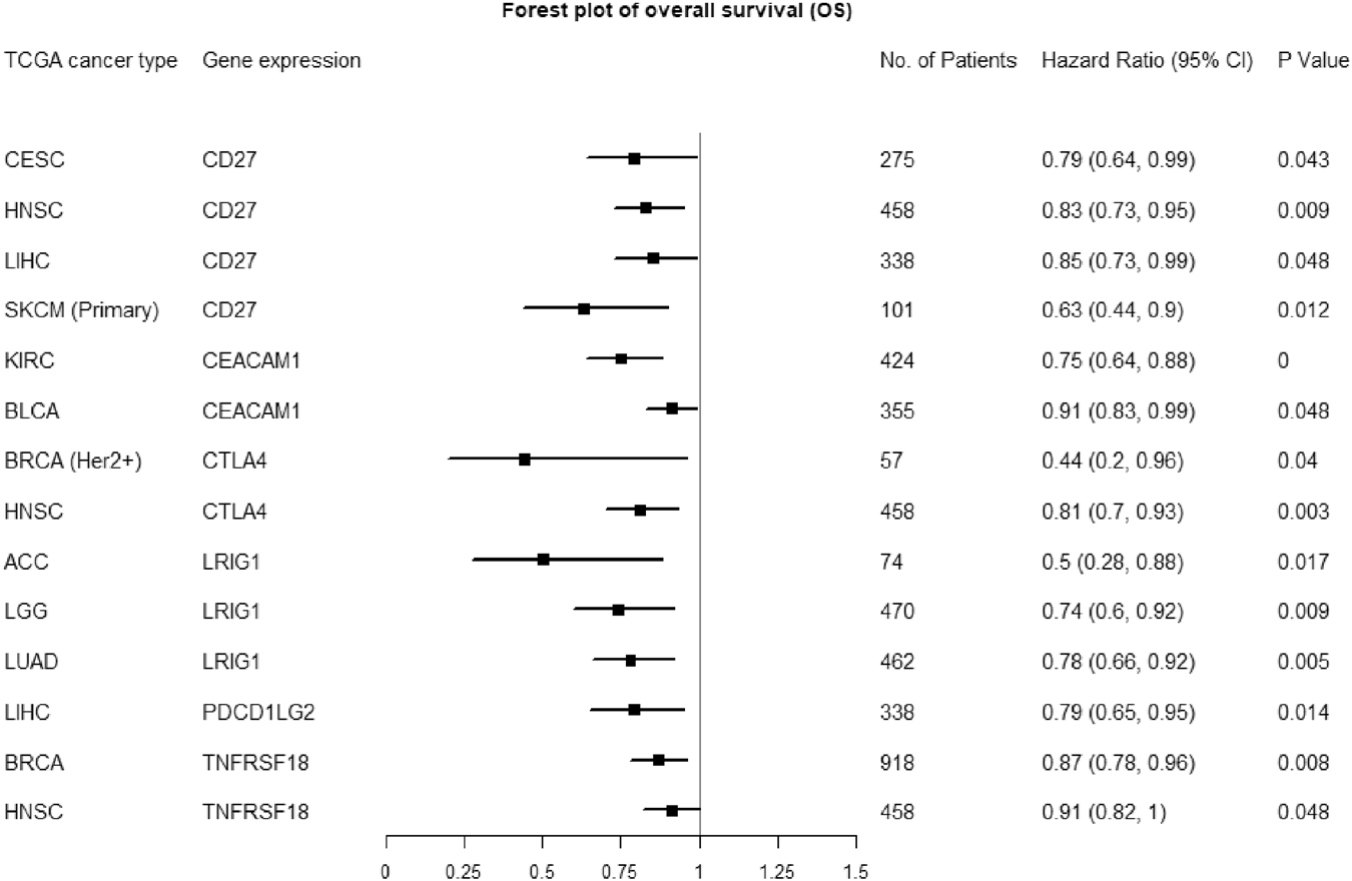
Forest plot of the hazard ratios for overall survival assessed by the expression levels of each of the six genes after correcting for the covariates of ‘tumor purity’ and the abundance of immune infiltrates. The higher expression of each gene from Immu6Metagene significantly associated with the prolonged overall survival in TCGA patient cohorts across different cancers.

In addition, we performed survival analysis of this set of 31 immune coinhibitory/costimulatory genes using the data from the TCGA database^20^. In total, 20 of the 31 genes showed significant associations with overall survival in at least one of the 25 types of TCGA cancers (Table 3). The potential roles of the six immune related genes identified by RNAi experiments as tumor suppressors were further supported by survival analyses. To obviate the confounding effects on survival analyses caused by the factors like the tumor purity and the tumor infiltrating immune cells, we analyzed the association of expression of these six genes with overall survival of TCGA cancer patients using the multivariable Cox proportional hazard model that corrected for multiple covariates of ‘tumor purity’ and the abundance of immune infiltrates. As the covariates that were adjusted in the multivariable Cox model, the abundances of the major immune infiltrates (B cells, CD8+ T cells, CD4+ T cells, Macrophages, Neutrphils, Dendritic cells, NK [Natural killer] cells and mast cells) were estimated by the statistical methods and validated using pathological estimations that were implemented in the TIMER approach by the previous study^23,24^. Take cancer type – CESC and gene – *CD27*, for example, the Cox proportional hazard model is given as follows: Surv(CESC) ~ Purity + B_cell + CD8^+^T-cell + CD4^+^T-cell + Macrophage + Neutrophil + Dendritic + NK_cell + mast_cell + CD27. Forest plot of the hazard ratios for overall survival assessed by the expression levels of each of the six genes after correcting for the covariates was shown in Fig. 4. The higher expression (i.e., above the median expression cutoff values) of each of the six genes – *CD27*, *CEACAM1*, *CTLA4*, *LRIG1*, *PD-L2*, and *TNFRSF18* significantly associated with the prolonged overall survival in TCGA patient cohorts, giving low hazard ratio (HR) values significantly less than 1 (ranging from 0.44 to 0.91) across different cancers. Representative Kaplan–Meier survival curves of these overall survival analyses were given in Fig. S7, which showed that the expression of each of the six genes served as the prognostic biomarker that independently predicted the better overall survival outcome after the correction for the abundance of tumor infiltrating immune cells. These suggested that the tumor suppressive effects of these six genes may be specific to the cancer cells since the significant effects of their expression on overall survival remained after adjusting for the the effects of abundance of tumor infiltrating immune cells on survival outcome.

**Table 3:** The higher expression of one or multiple immune checkpoint genes of the 31-gene set was associated with prolonged overall survival in each of the 25 TCGA cancer types.

TCGA cancer type	Abbreviation	Immune coinhibitory/costimulatory genes whose lower expression associated with shorter overall survival
Adrenocortical carcinoma	ACC	CD40, CD274 (PD-L1), LRIG1
Bladder urothelial carcinoma	BLCA	CEACAM1, TNFRSF18
Breast invasive carcinoma	BRCA	TNFRSF18, CD40LG, CLEC4G, TNFRSF4, C10orf54 (VISTA), CTLA4
Cervical and endocervical cancers	CESC	CD27, LGALS9
Colorectal adenocarcinoma	COADREAD	TIGIT, CTLA4, ICOS, LGALS9, PDCD1 (PD-1)
Glioblastoma multiforme	GBM	TNFSF18
Glioma	GBMLGG	LRIG1
Head and neck squamous cell carcinoma	HNSC	TNFRSF18, CD27, CTLA4, TNFRSF4
Kidney chromophobe	KICH	PVRL2 (NECTIN2)
Kidney renal clear cell carcinoma	KIRC	CD274 (PD-L1), CEACAM1, LRIG3
Brain lower grade glioma	LGG	LRIG1
Liver hepatocellular carcinoma	LIHC	CD27, PDCD1LG2 (PD-L2)
Lung adenocarcinoma	LUAD	ICOSLG, LRIG1, LRIG2, LRIG3
Lung squamous cell carcinoma	LUSC	TNFRSF18
Mesothelioma	MESO	C10orf54 (VISTA)
Ovarian serous cystadenocarcinoma	OV	CD40
Pancreatic adenocarcinoma	PAAD	TNFRSF4
Rectum adenocarcinoma	READ	ICOS, PDCD1 (PD-1), PDCD1LG2 (PD-L2), TNFRSF9
Sarcoma	SARC	TNFRSF4, CD40, CD40LG, LGALS9, C10orf54 (VISTA)
Skin Cutaneous Melanoma	SKCM	CD27, TNFRSF18
Stomach adenocarcinoma	STAD	TIGIT, LGALS9, PDCD1 (PD-1), C10orf54 (VISTA)
Stomach and Esophageal carcinoma	STES	TNFSF4, CD40, CD70, PDCD1LG2 (PD-L2), TNFRSF18
Thymoma	THYM	ICOS
Uterine Carcinosarcoma	UCS	ICOS
Uveal Melanoma	UVM	CD40, PDCD1LG2 (PD-L2)

### Microarray studies validated that the higher expression of the metagene expression signature derived from six immunity related genes was associated with prolonged survival phenotypes in cancer patients

**Figure 5:**
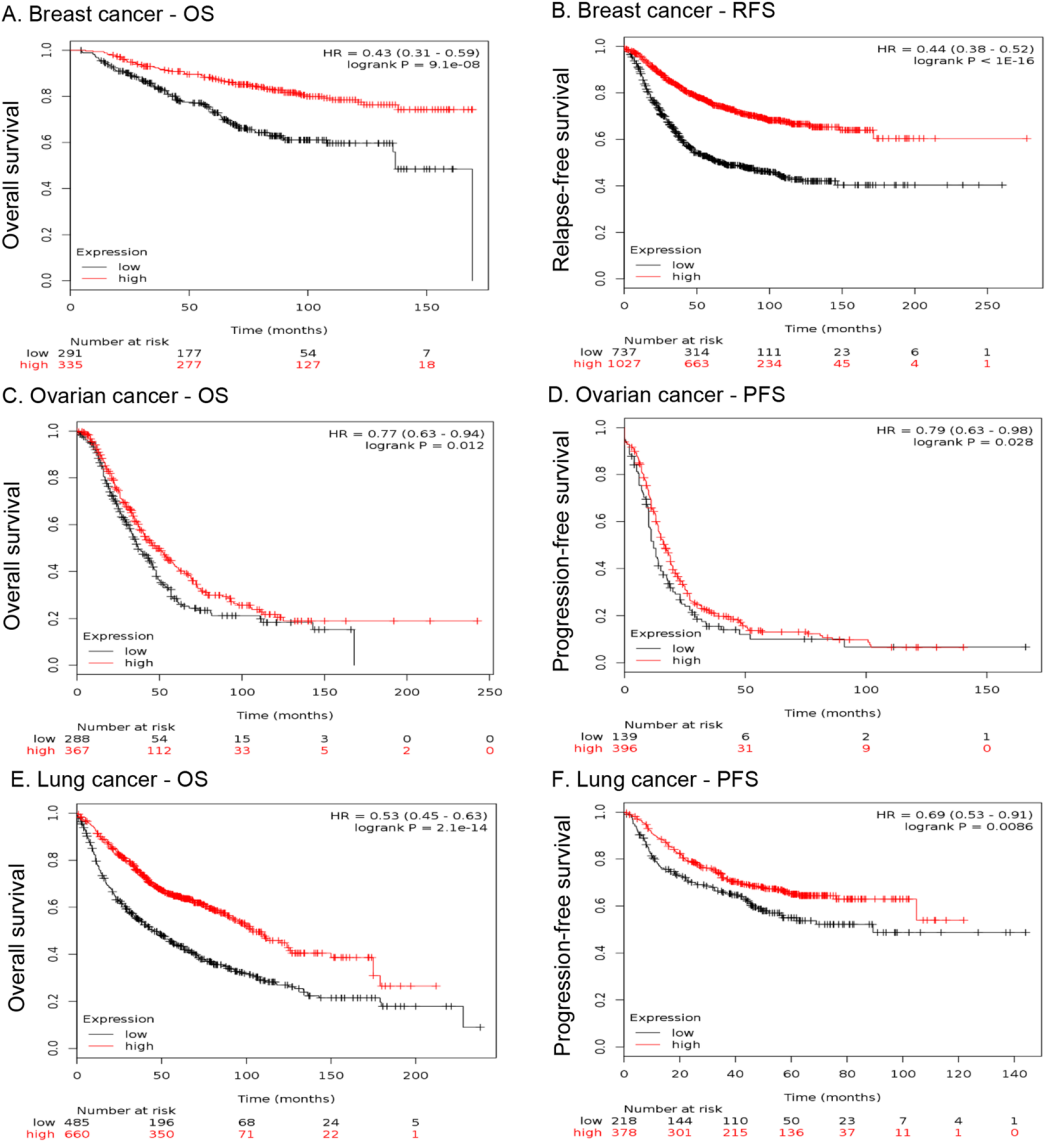
Six immune related genes of the metagene signature - Immu6Metagene showed significant prognostic value in cancer survival based on microarray datasets from GEO. KM plots of OS and PFS/RFS probability of breast cancer patients (**A**,**B**), ovarian cancer patients (**C**,**D**) and lung cancer patients (**E**,**F**) are shown.

The above results highlighted immune coinhibitory/costimulatory genes as tumor suppressors, especially for the six genes - *CD27*, *CEACAM1*, *CTLA4*, *LRIG1*, *PDCD1LG2*, and *TNFRSF18*, which had been supported by the data from mutation, expression, functional screening and survival analyses. This led to the hypothesis that the metagene signature composed of these six genes could be important prognostic biomarker whose higher expression associated with good clinical outcome. The above survival analyses based on the TCGA RNA-seq data already showed the ‘good prognostic’ implications of the individual genes of this metagene signature, which was called as “Immu6Metagene”. To validate the Immu6Metagene signature, we used the online KMPlotter platform^25–27^ to estimate the prognostic impact of the Immu6Metagene signature on the breast, ovarian and lung cancer patients (Details can be seen in Methods section). The respective patients have been stratified into high (red lines) or low (black lines) expression group by considering the mean of median transcript-expressions of *CD27*, *CEACAM1*, *CTLA4*, *LRIG1*, *PDCD1LG2*, and *TNFRSF18*. In other word, mean of combined expression of respective gene-probe sets were utilized to quantify the Immu6Metagene signature expression. As can be seen from Fig. 5, higher expressions of Immu6Metagene signature were significantly associated with prolonged OS (overall survival) in breast (Fig. 5A), ovarian (Fig. 5C), and lung cancer patients (Fig. 5E). In addition, higher expressions of Immu6Metagene signature were significantly associated with prolonged progression-free survival (PFS) or recurrence-free survival (RFS) in breast (Fig. 5B), ovarian (Fig. 5D), and lung cancer patients (Fig. 5F).

For independent validation of the microarray results from KMPlotter, we also assessed the effect of the Immu6Metagene signature on overall survival using the SurvExpress program to analyze the RNA-seq data of the TCGA cancer cohorts of BRCA (Breast invasive carcinoma), CESC (Cervical and endocervical cancers) and LUAD (Lung adenocarcinoma). As shown in Fig. S8, the higher Immu6Metagene signature expressions associated with the low risk groups (prolonged survival) in different cancer types were driven by different combinations of the six genes. In BRCA, the higher 6-gene signature expression in the low risk group compared to the high-risk group was driven by the higher expressions of the 5 genes, i.e., *CD27*, *CEACAM1*, *CTLA4*, *LRIG1*, and *TNFRSF18* (Fig. S8A,B). In CESC, the higher 6-gene signature expression in the low risk group was driven by the higher expressions of another set of 5 genes, i.e., *CD27*, *CEACAM1*, *CTLA4*, *PDCD1LG2*, and *TNFRSF18* (Fig. S8C,D). In LUAD, the higher 6-gene signature expression in the low risk group was driven by the higher expressions of the 4 genes, i.e., *CD27*, *CEACAM1*, *CTLA4*, and *LRIG1* (Fig. S8E,F). These further supported that higher expressions of the six genes contributed to the prolonged survival in the low risk groups across different cancers.

In addition, we analyzed the DNA methylation levels of the six genes of the Immu6Metagene signature in terms of their associations with overall survival using the TCGA datasets. It was found that higher DNA methylation levels of these six genes were significantly associated with worse overall survival outcomes in the TCGA cancer cohorts of BRCA, CESC and LUAD (Fig. S9). Because DNA methylation usually correlates negatively with corresponding gene expression^28,29^, these data further supported the results of our survival analyses of the six genes of the Immu6Metagene signature.

### Oncogenic pathways were inhibited in the tumors of cancer patients with good survival outcome characterized by the higher expression of the Immu6Metagene signature

**Figure 6:**
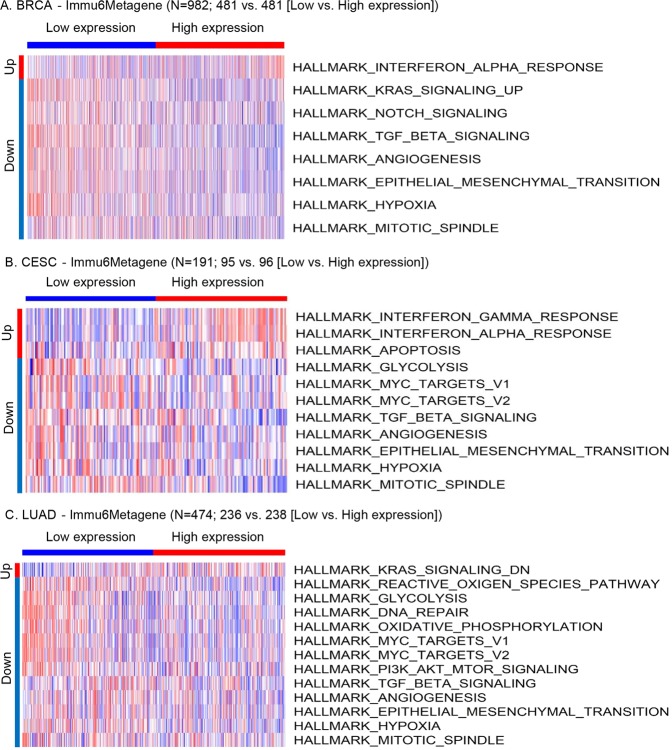
Hallmark gene set activity changes across the TCGA cancer patients with low or high Immu6Metagene signature expression levels. The significant pathway changes were shown for three cancer types: (**A**) Breast cancer (BRCA); (**B**) Cervical and endocervical cancers (CESC); (**C**) Lung adenocarcinoma.

Because the higher and lower expression levels of the Immu6Metagene signature separated the low-risk group with good survival outcome from the high-risk group with poor survival outcome, it is interesting to investigate whether there were significant pathway activity changes between the low risk group with high Immu6Metagene expression and the high-risk group with low Immu6Metagene expression. Using the GSVA method^30^ and the Molecular Signatures Database (MSigDB) hallmark gene set collection^31,32^, we analyzed the RNA-seq data of the TCGA cancer cohorts of BRCA, CESC and LUAD. In the tumor samples of the BRCA cohort, compared to the patients of the low Immu6Metagene signature expression group, the patients of the high expression group had significantly elevated interferon alpha (IFNα) response and significantly inhibited activity of multiple oncogenic pathways including Kras signaling, Notch signaling, TGF-β signaling, angiogenesis, EMT (epithelial mesenchymal transition), hypoxia and mitotic process (Fig. 6A). As for the tumors of the CESC cohort, interferon alpha (IFNα) and gamma (IFNγ) responses and apoptosis of cancer cells were significantly elevated while the oncogenesis related pathways of glycolysis, MYC signaling, TGF-β signaling, angiogenesis, EMT, hypoxia and mitotic process were significantly inhibited in the patients of the high Immu6Metagene signature expression compared to the low expression (Fig. 6B). For the tumors of the LUAD cohort, a large number of oncogenic pathways were inhibited in the high Immu6Metagene signature expression group compared to the low expression group, including KRAS signaling, reactive oxygen species pathway, glycolysis, DNA repair, oxidative phosphorylation, MYC signaling, PI3K-AKT-MTOR signaling, TGF-β signaling, angiogenesis, EMT, hypoxia and mitotic process (Fig. 6C). Interestingly, these three types of cancers had a common set of five oncogenesis related pathways that were significantly inhibited in the tumors of the good prognostic patients with high Immu6Metagene signature expression. These pathways were TGF-β signaling, angiogenesis, EMT, hypoxia and mitotic process (Fig. 6). These analyses indicated that higher expression of the Immu6Metagene signature could be associated with the suppressed activities of the above five oncogenic pathways, which may contribute to the good survival outcome in cancer patients.

## Discussion

In this study, we utilized the CCLE data resources to characterize the potential role of T-cell coinhibitory/costimulatory molecules in the context of cancer cells. A considerable fraction of cancer cell lines had nonsilent somatic mutations in these genes. Especially, more than 90% of the immune coinhibitory/costimulatory genes (28/31) were not expressed at all or only expressed at low levels across the majority of the 1103 CCLE cell lines originating from 22 tissues. These 28 genes were also downregulated in the tumor samples compared to the normal tissues across many TCGA cancer types. The above evidence suggested that the T-cell immunity related genes were largely inactivated in the cancer cells. By analyzing the RNAi screening data from the cancer dependency map project^19^, we further identified six genes - *CD27*, *CEACAM1*, *CTLA4*, *LRIG1*, *PDCD1LG2*, and *TNFRSF18*, the knockdown of which resulted in the enrichment of corresponding cancer cells expressing their respective targeting shRNAs. The survival analyses of the TCGA data after adjusting for the abundances of tumor infiltrating immune cells showed that the higher expression of the individual gene of this Immu6Metagene signature was significantly associated with the prolonged survival phenotypes in the cancer patients. Microarray studies further validated the significant association of Immu6Metagene signature expression with good survival outcomes. These results matched the findings from the RNAi screening experiments.

Although having strengths that include ease of use, low cost, and utility in diverse experimental studies, cancer cell lines are not perfect models in the study of cancer immunology. A main issue with the cancer cell line models is that important components of the TME (tumor microenvironment) are lacking, including cells of the immune system. The cancer cell line models are the approximations of tumors and cannot fully recapitulate the aspects of the tumor microenvironment especially the immune system within TME. Therefore, a limitation of our study is that the data from cell lines may be indicative but not representative. However, the results of the six genes of the Immu6Metagene signature had been consistent across both cancer cell line and tumor tissue samples and therefore more robust.

Another limitation is that mRNA expression does not necessarily correlates with protein expression. Hence, the final proof of the concept of this study can only be done with proper immunohistochemistry localizing expression levels of immune-regulatory proteins specifically on tumor cells only with subsequent comparison to survival. It is also worth mentioning that different types of immune infiltrates in tumors may influence the tumor gene expression beyond what the statistical modeling can adjust for. Moreover, although the roles of some types of stromal cells like cancer-associated fibroblasts (CAFs) and tumor vasculature were more ‘tumor-like’ and different from ‘healthy’ tissues, the stromal cells’ influence on tumor expression may still need to be considered since the tumor data like the TCGA data may contain certain stromal cell information that may be falsely interpreted as tumor cell expression. To adequately avoid the effects of immune infiltrates and ‘normal-like’ stromal cells on tumor expression, the single cell RNA-sequencing (scRNA-seq) approach may be necessary since it can clearly separate the large amount of cells within a tumor sample into distinct populations of pure tumor cells, immune infiltrating cells and stromal cells etc. to profile each individual cell populations’ gene expression at the whole transcriptome level. This will be the direction for our future studies.

Among the six genes comprising the Immu6Metagene signature, CD27 is a well-known costimulatory receptor on T cells. CD27 is constitutively present on all subsets of T cells^33^, a subset of NK cells^34^, and memory B cells^35^. Another name of CD27 is TNFRSF7 and it is a member of the tumor necrosis factor receptor (TNFR) superfamily and exists as a type 1 transmembrane, disulfide-linked homodimer^33^. CD27 signaling can improve T-cell function^36^. The CD70-CD27 interaction leads to recruitment of TNFR-associated factor (TRAF) proteins to the CD27 cytoplasmic tail^37,38^, which subsequently elicit cellular responses involving CD8+ T cell priming, proliferation, survival, and cytotoxicity^39–41^. The anti-tumor effect of CD27 ligation has been shown in different cancers, for example, the agonist Anti-CD27 Antibodies, i.e., the antibodies that activate CD27 rather than block its activity (like antagonistic antibodies), demonstrated the anti-tumor efficacy in both animal models^42,43^ and human phase I trials^44^. Although being well studied in T-cell immunity, the role of CD27 in cancer cells was not known well. Our study showed that *CD27* mRNA expression was lost in more than 98% of the 1103 CCLE cancer cell lines from 22 tissues (Fig. S4, #2). Its gene expression levels were also very low in the tumors across the distinct cancer types (Fig. S10A). The higher expression of *CD27* was a favorable prognostic marker and associated with good survival outcomes in multiple cancers like CESC, HNSC, LIHC and SKCM after adjusting for the abundances of tumor infiltrating immune cells (Fig. 4, Figs S7 and S11). These plus the RNAi data supported that CD27 may suppress the tumor progression or TME development in some types of cancers.

*CEACAM1* encodes the molecule that is a ligand for TIM3 (T-cell immunoglobulin domain and mucin domain-3, also known as HAVCR2)^13^. TIM3 has been shown to induce T-cell exhaustion in cancers and is being targeted for cancer immunotherapy^45^. TIM3 is co-expressed and forms a heterodimer with CEACAM1, which endows TIM3 with T-cell inhibitory function. In the lymphocytes infiltrated microenvironment, CEACAM1 and TIM3 mark exhausted T cells and co-blockade of them leads to enhancement of anti-tumor immune responses with improved elimination of tumors^45^. However, recent novel findings suggested that TIM3 is actually more similar to costimulatory receptors that are up-regulated after T cell activation than to a dominant inhibitory protein like PD-1^46^. This indicated that the roles of CEACAM1 and TIM3 are more complex than previously thought. In this study, we revealed that CEACAM1 may be a suppressive factor in tumor cells. *CEACAM1* had no or low expression in most of the cancer cell lines and various cancer types (Fig. S4, #6; Fig. S10B). The knockdown of CEACAM1 by RNAi experiment resulted in the growth advantage of the corresponding cancer cells with no or low CEACAM1 expression (Fig. 2). Higher expression of *CEACAM1* gene was associated with prolonged survival in KIRC and BLCA cancer patients after adjusting for the abundances of tumor infiltrating immune cells (Figs 4 and S7). Therefore, the role of CEACAM1 in cancer cells may be different from its role of inhibiting anti-tumor immune responses in T cells. Our findings were supported by the previous research pointing to the tumor suppressor role of CEACAM1^47–55^. A most recent study suggested that CEACAM1 overexpression significantly suppressed cancer cell proliferation, induced cancer cell apoptosis, and inhibited cancer cell invasion and migration possibly through activation of caspase-3 and downregulation of MMP-2 and MMP-9^47^. These supported our conclusion of the tumor suppressor role of *CEACAM1*.

CTLA4 is mostly known for its role in inhibiting TCR (T-cell receptor) signaling and subsequent T-cell activation through competition with the costimulatory molecule CD28 for the B7 ligands B7-1 (CD80) and B7-2 (CD86)^13^. Anti-CTLA4 immunotherapy is based on this principle and has achieved great success. CTLA4 had no expression in nearly all the cancer cell lines (Fig. S4, #7) and many different cancer types (Fig. S10C). RNAi experiments followed up by NGS (next generation sequencing) showed that the cancer cells with *CTLA4* knocked down gained proliferation advantages (Fig. 2). Higher expressions of CTLA4 were significantly associated with the good survival outcomes in the TCGA BRCA (Her2+) and HNSC cancer patients after adjusting for the abundances of tumor infiltrating immune cells (Figs 4 and S7). The potential suppressive role of CTLA4 in tumors seems paradoxical to the prototypical role of CTLA4 in suppressing antitumor immunity. However, this is similar to the case of PD-1, which is another prototypical inhibitor of antitumor immunity that had recently been identified as a novel tumor suppressor^16,17^. PD-1 was also indicated as a potential tumor suppressor across multiple cancers based on our data (Tables 2 an 3; Fig. S4, #16; Fig. S6, #4). Back to discussing CTLA4, basic science research also lent support to our results. For example, genetically predisposed *CTLA4* insufficiency in humans has been associated with gastric cancer (GC) development^56–58^. The risk alleles of *CTLA4* promoter and exon 1 linked to GC^59,60^ led to the reduction of CTLA4 expression^61–63^. It was also found that CTLA4 insufficiency initiates *de novo* tumorigenesis in the mouse stomach through type 2 inflammation, with age-associated progression to malignancy accompanied by epigenetic dysregulation^64^. These and our new data are conceptually consistent and suggest that CTLA4 blockade therapy may promote tumorigenesis under certain circumstances.

LRIG1 was rarely studied in the oncoimmunology context before. However, LRIG1 may be a novel T cell costimulatory receptor that is required for optimal anti-tumor T cell activation (the related information can be seen on the following webpage - https://app.dimensions.ai/details/grant/grant.6663751). In addition, LRIG1 has been identified as containing multiple regions that could be the bindings site of FOXP3 in human regulatory T cells (Tregs)^65^. FOXP3 is the important transcription factor that is essential for the formation and function of regulatory T cells (Tregs), which are essential for maintaining immune homeostasis and tolerance. Therefore, LRIG1 could be an important FOXP3 targeting gene playing a role in oncoimmunology^65^. Here we studied LRIG1 from the cancer cells perspective. *LRIG1* has no or low expression in more than 80% across all the cancer cell lines (Fig. S4, #13) and different cancer types (Fig. S10D). It is also downregulated in many types of tumors relative to the control normal samples (Table 2 & Fig. S6, #14). The knockdown of *LRIG1* caused the more favorable proliferation of the corresponding 22 cancer cell lines (Figs 2 and 3). Higher expression of *LRIG1* was a good prognostic biomarker associated with the prolonged survival outcomes in the ACC, LGG and LUAD cancer patients after adjusting for the abundances of tumor infiltrating immune cells (Figs 4 and S7). Previous studies largely supported our results by showing the tumor suppressor role of *LRIG1* in multiple types of cancers^66–73^. Our data indicated that the drugs stimulating LRIG1 activity such as LRIG1-specific agonistic mAbs (monoclonal antibodies) could be a novel strategy for cancer therapy.

In addition, this study suggested that PDCD1LG2 (PD-L2) and TNFRSF18 could be the suppressor of tumor cells. PD-1 attenuates the anti-tumor T-cell responses through interaction with its ligands PD-L1 and PD-L2^13^. TNFRSF18 is also known as GITR, which functions as an important costimulatory molecule that enhances anti-tumor actions of effector T cells^13^. Therefore, previous research showed that the roles of PD-L2 and TNFRSF18 are opposite in cancer immunity, with the former and the latter being coinhibitory and costimulatory molecules for anti-tumor T-cell responses, respectively. However, they may both be the suppressive factors of tumor cells. We observed that they did not express or only had very low expression across about 99% of the cancer cell lines (Fig. S4, #17 and #19) and various types of cancers (Figure S10E, S10F). The RNAi screening experiments validated that the loss of expression of them contributed to the corresponding cancer cell proliferation (Fig. 3). The higher expression of *PD-L2* or *TNFRSF18* was significantly associated with good survival outcome in LIHC or BRCA/HNSC cancer patients, respectively, after adjusting for the abundances of tumor infiltrating immune cells (Figs 4 and S7). PD-L2’s potential role in inhibiting tumorigenesis may be mediated through PD-1 because that the previous studies have shown that the interaction between tumor-intrinsic PD-1 and its ligands like PD-L1/PD-L2 inhibits tumor progression^16,74^. In terms of *TNFRSF18*, it is inactivated during tumor progression in Multiple Myeloma (MM) through promoter CpG island methylation, leading to gene silencing in primary MM cells and MM cell lines^75^. Restoration of *TNFRSF18* expression in *TNFRSF18* deficient MM cells led to inhibition of MM proliferation *in vitro* and *in vivo* and induction of apoptosis^75^. Such anti-tumor function of TNFRSF18 may be mediated through the induction of p21 and PUMA, two direct downstream targets of p53, together with modulation of NF-κB in TNFRSF18-overexpressing MM cells^75^.

So far, the data relating knockout of the six genes of the Immu6Metagene signature to the outcomes of cancer incidence are lacking. However, there are diverse emerging evidence including mice gene knockout experiments supporting our findings. A recent study showed that CD27 stimulation activated the transcriptional programs that synergize for CD8 + T-cell-driven antitumor immunity^76^. Specifically, they showed that a clinically relevant agonist anti-human CD27 mAb (an activating CD27 antibody), varlilumab, contributes to the protection against lymphoma in human-CD27 transgenic mice^76^. The experiments investigating CEACAM1 in tumorigenesis denoted a pivotal role for CEACAM1 as a tumor suppressor. For example, prostate cancer cell line PC-3 transfected with CEACAM-1 demonstrated significantly lower growth rates and less tumorigenicity *in vivo* relative to controls^77^. The absence of CEACAM1 on hyperplastic tumors correlated with reduced apoptosis of malignant cells^52^. Moreover, *CEACAM1* knockout mice lacking CEACAM1 in WAP-T tumor cells had enhanced tumor phenotypes including increased Wnt signaling, promoted cellular invasiveness, and strongly enhanced rate of metastasis of mammary adenocarcinomas *in vivo*^78^. Miska *et al*. performed an innovative study by creating transgenic CTLA4 shRNA knockdown (CTLA4KD) mice to mimic CTLA4 insufficiency in humans^79^. They found that CTLA4 insufficiency, modeled by CTLA4KD or antibody blockade, caused the initiation of inflammatory tumorigenesis in the stomach of mice with susceptible genetic backgrounds^79^, which established the causality of CTLA4 insufficiency in gastric cancer and the tumor suppressive role of CTLA4. As for LRIG1, A recent knockout mouse study showed that *Lrig1* is a haploinsufficient tumor suppressor gene in malignant glioma. This was demonstrated by the experiment revealing that *Lrig1* KO mice developed higher grade gliomas than did wild-type mice^68^. Reciprocally, the ectopic expression of LRIG1 in the high-grade human glioma cell line decreased the invasion of orthotopic tumors in immunocompromised mice *in vivo* and reduced cell migration *in vitro*^68^. In fact, LRIG1 has arrived on the cancer biology scene as a tumor suppressor evidenced by KO mice studies in different types of cancers involving skin, intestine, lung, eye and other cancers as well summarized in a review article^80^. Till now, we did not find PDCD1LG2 (PD-L2) KO mice study in cancer context. However, previous research did show that expression of PD-L2 on the tumor cells promotes CD8 T cell–mediated rejection of tumor cells, at both the induction and effector phase of antitumor immunity^81^. Moreover, PD-L2 enhanced T cell killing of tumor cells in a PD-1–independent mechanism^81^. Similar to PD-L2, no knockout study of TNFRSF18 (GITR) to dissect its role in cancer was found currently. Yet, novel tumor suppressor function of TNFRSF18 had begun to be uncovered especially in the case of multiple myeloma, which is based on the observation that the tumor cell proliferation was significantly inhibited both *in vitro* and *in vivo* in mice injected with TNFRSF18 compared to the empty control^75^. Overall, the previous studies especially the knockout studies in mice suggested the novel tumor suppressor functions of the six genes involved in the identified Immu6Metagene signature.

An interesting question is whether the expression levels of the six genes of the Immu6Metagene signature in the normal cells were comparable to the immune cells in which they are known to play an important role. If the expressions of these genes in normal cells were not detected or extremely low compared to the immune cells, they may not be important to tumor cell biology. To answer this question, we downloaded the mouse data via the ‘Gene Skyline’ browser (http://rstats.immgen.org/Skyline/skyline.html) from the Immunological Genome project^82^ and compared the gene expression levels of these 6 genes across the T cells, dendritic cells (DCs) and normal epithelial cells. The data showed that these six genes were all considerably expressed in the normal epithelial cells with normalized counts ranging from 30 to 203 (Fig. S12). *Cd27* expression in the normal epithelial cells was lower than the CD4+ and CD8+ T cells. However, normal epithelial cells still express about 3% of *Cd27* as T cells. The epithelial cells’ *Cd27* expression value is 50.4, which is the medium expression status (Methods section) and similar to the *Cd27* level in DCs (Fig. S12) and thus cannot be neglected. The expression of *Tnfrsf18* (*Gitr*) in the normal epithelial cells was about 30, which is 11.0% and 15.1% of the *Tnfrsf18* expressions in the T cells and DCs, respectively (Fig. S12). In addition, the expression values of *Ceacam1*, *Ctla4*, *Lrig1*, and *Pdcd1lg2* were even higher in the normal epithelial cells than the immune cells including T cells and DCs (Fig. S12). These indicated that the six genes in the Immu6Metagene signature express and function normally in the healthy normal cells such as epithelial cells.

Malignant melanoma is the ideal and most frequently studied model tumor for oncoimmunology. Previous research showed that melanoma contains the most mutations^83^ and hence – in theory – can be addressed by the broadest CD8 TCR repertoire. However, even for this well-studied oncoimmunology model, little is known about the effective tumor and tumor-microenvironment (TME) biomarkers that are highly prognostic of malignant melanoma and of anti-tumor response. It remains largely unclear what molecular mechanisms govern T cell infiltration. Although the presence of immunogenic antigens is thought to be necessary for ICBT response, large-scale analyses of hundreds of melanomas in TCGA suggested that lack of antigens in the TME are unlikely to be the rate-limiting step in anti-tumor immunity^84^. Multiple pathways and aberrations in tumor cell signaling such as the Ras/MAPK pathway^85^, WNT/β-catenin^86,87^, PTEN/PI3K pathways^88^ and a novel transcriptional program termed IPRES (innate PD-1 resistance)^89^ have been analyzed in terms of their associations with absence of a T cell infiltrate and anti-tumor immune responses. However, these mutational and transcriptional alterations have more value as a roadmap to tumor-immune interactions rather than as true predictive biomarkers. Our study suggested that the roles of immune checkpoints in tumor cells *per se* may also need to be considered when assessing the effects of different factors on the efficacy of anti-tumor immunotherapy.

Finally, the pathway analysis across multiple types of cancers revealed that a common set of five oncogenic pathways were significantly inhibited in the tumors of the patients with good survival outcome and high Immu6Metagene signature expression (Fig. 6). These pathways were TGF-β signaling, angiogenesis, EMT, hypoxia and mitotic process. A particularly interesting finding is the downregulation of TGF-β signaling in good prognostic patients who had higher Immu6Metagene signature expression. TGF-β signaling pathway was found to play an important role in resistance to immunotherapy. For example, Mariathasan *et al*. reported that unresponsiveness to PD-L1 blockade was associated with TGF-β signaling in fibroblasts and indicated that TGF-β-mediated stromal remodeling restricts T-cell infiltration to suppress antitumor immunity and that TGF-β inhibition may enhance the efficacy of immune checkpoint blockade^90^. In parallel, Tauriello *et al*. found that single-agent PD-1/PD-L1 inhibition had little effect, but co-targeting TGF-β produced a robust antitumor immune response that could prevent the development of metastasis and eliminate established metastases in a mouse model^91^. Collectively, these studies indicate that inhibiting TGF-β could significantly improve the efficacy of anti-PD-1/anti-PD-L1 treatment^90,91^. Herein, our data suggested that enhanced TGF-β signaling could result from the blockade of checkpoint molecules involved in the Immu6Metagene signature such as CEACAM1 and CTLA4. Therefore, ICBT of these genes may also consider the blockade of TGF-β signaling simultaneously to improve the efficacy of the treatment.

In summary, this study revealed the tumor-suppressive function of the T-cell immunity related genes especially of the six genes of the Immu6Metagene signature. Our conclusions were consistent with those of the previous research. For instance, the expression of *CD27*, *CTLA4*, *PDCD1LG2*; *TNFRSF18* were all significantly inhibited across different types of TCGA cancers as can be seen in the comprehensive study of more than 10,000 tumors comprising 33 diverse cancer types by utilizing data compiled by TCGA^92^. This supported our finding that the Immu6Metagene signature genes may play the tumor suppressor role across different cancers. Accordingly, if immunotherapy targeting these molecules is considered, caution must be taken to avoid the potential adverse clinical outcomes including the hyperprogressive diseases resulting from the immunotherapy.

## Methods

### Mutation and gene expression analysis of the CCLE cell lines

The analysis focused on the set of 31 prominent immune coinhibitory/costimulatory genes (Table 1) proposed by the recent comprehensive review article for the progress made in the field of the immune checkpoint blockade therapies (ICBT)^13^. The mutation data from 1509 CCLE cell lines originating from the 22 tissues were downloaded from CCLE website (https://data.broadinstitute.org/ccle/). The data file used was “CCLE_DepMap_18q3_maf_20180718.txt”. Germline mutations and SNPs archived in the public databases were filtered. The SNP databases that we used for filtering include dbSNP build 137, 1000 Genomes, the NHLBI ESP6500 dataset, and the 69-whole-genome dataset (variant calls and allele-frequency information) from Complete Genomics. The gene expression data of the 1103 CCLE cell lines from the 22 tissues were also downloaded and analyzed. The data file used was “CCLE_DepMap_18q3_RNAseq_RPKM_20180718.gct”. Plots of mutations were generated using the “oncoPrint” function provided by the R package – ComplexHeatmap^93^. We convert the RPKM (Reads Per Kilobase Million) values to a log2 scale and do an ordinary limma analysis in the same way as for microarray data, using eBayes() function with trend = TRUE, which was implemented in the limma software^94^. FDR (False discovery rate) corrected *P*-values of less than 0.05 were used as criteria for significantly regulated genes across mutation vs non-mutation groups in terms of the 31 genes. The gene expression heatmaps was generated using the R package – heatmap3 (https://cran.r-project.org/web/packages/heatmap3/).

### Analysis of the expression of immune coinhibitory/costimulatory genes in the CCLE cell lines stratified by their tissue origins

According to the criteria established previously for RNA-seq based gene expression experiments by the “Expression Atlas” resource (https://www.ebi.ac.uk/gxa/FAQ.html), the expression status of a gene can be classified into one of four categories: 1) Not expressed (no expression): expression level is below cutoff (0.5 RPKM); 2) Low expression: expression level is low (between 0.5 to 10 RPKM); 3) Medium expression: expression level is medium (between 10 to 1000 RPKM); 4) High expression: expression level is high (more than 1000 RPKM). Based on these criteria and the RNA-seq data, we analyzed the 31 immune coinhibitory/costimulatory genes across the 1103 CCLE cancer cell lines from 22 tissue origins and made the polar histograms for each of the 31 genes (Figs S3–S5).

### Analysis of the data of the previous RNAi screening experiments

To systemically assess the roles of the 31 immune coinhibitory/costimulatory genes in cancer cells, we analyzed 501 genome-scale loss-of-function screens performed in diverse human cancer cell lines by the Cancer Dependency Map project (https://depmap.org/portal/)^19^. Specifically, the file named “ExpandedGeneZSolsCleaned.csv” recording the data of gene knockdown effect in cell lines inferred by the DEMETER computational model was downloaded from the Broad Institute Project Achilles webpage (https://portals.broadinstitute.org/achilles/datasets/15/download). As for the DEMETER results^19^, a lower dependency score means that a gene is more likely to be an oncogene essential to cancer cell proliferation. The deletion of such gene by its shRNAs resulted in the lower proportion of cancer cells expressing those shRNAs, which can be detected by NGS (next-generation-sequencing) and finally reflected by the lower score. On the contrary, the high dependency scores meant that the corresponding genes were not essential to cancer cell proliferation and the top ranked scores indicated the corresponding genes may function as tumor suppressors because of the enrichment of the cancer cells expressing their targeting shRNAs that knocked down such genes^19,22^.

### Survival analysis based on the data sets from the TCGA and GEO databases

To investigate the possible tumor suppressor roles indicated by the overall low expression of immune coinhibitory/costimulatory genes in cancer cell lines, we downloaded the TCGA (The Cancer Genome Atlas)^20^ clinical and gene expression data from the Broad Institute TCGA GDAC Firehose project (https://gdac.broadinstitute.org/) and performed survival analyses on the genes of this immune gene set (Table 1) using The R Project for Statistical Computing (https://www.r-project.org/). To obviate the confounding effects on survival analyses caused by the factors like the tumor purity and the tumor infiltrating immune cells, we analyzed the the six genes of the Immu6Metagene signature (i.e., *CD27*, *CEACAM1*, *CTLA4*, *LRIG1*, *PDCD1LG2*, and *TNFRSF18*) using the multivariable Cox proportional hazard model that corrected for multiple covariates of ‘tumor purity’ and the abundance of immune infiltrates. As the covariates that were adjusted in the multivariable Cox model, the abundances of six immune infiltrates (B cells, CD8+ T cells, CD4+ T cells, Macrophages, Neutrphils, and Dendritic cells) were estimated by the statistical methods and validated using pathological estimations that were implemented in the TIMER approach by the previous study^23,24^. Take cancer type – CESC and gene – *CD27*, for example, the Cox proportional hazard model is given as follows: Surv(CESC) ~ Purity + B_cell + CD8^+^T-cell + CD4^+^T-cell + Macrophage + Neutrophil + Dendritic + CD27. Forest plot of the hazard ratios for overall survival assessed by the expression levels of each of the six genes after correcting for the covariates was shown in Fig. 4. The forest plot of hazard ratios for overall survival was done using the R package ‘forestplot’ (https://cran.r-project.org/web/packages/forestplot/). For plotting the Kaplan–Meier survival curves of these overall survival analyses as shown in Fig. S7, the patients were dichotomized according to the cutoff of the median expression values of a specific gene, with the subjects having the specific gene expression larger than the cutoff being defined as higher expression patients and lower than the cutoff being defined as lower expression patients. The logrank p-value were adjusted for multiple testing using the Bonferroni method. The gene expression-based Kaplan–Meier survival analysis was also performed using the TCGA data from SurvExpress (http://bioinformatica.mty.itesm.mx:8080/Biomatec/SurvivaX.jsp) and from THE HUMAN PROTEIN ATLAS database (https://www.proteinatlas.org/), respectively. The DNA methylation-based survival analysis was conducted using the TCGA data in the website resource of MethSurv (https://biit.cs.ut.ee/methsurv/). To validate the Immu6Metagene signature composed of the six genes - *CD27*, *CEACAM1*, *CTLA4*, *LRIG1*, *PDCD1LG2*, and *TNFRSF18*, we used the KMPlotter platform^25–27^ to perform online survival analyses as implemented in the following website: http://kmplot.com/analysis/. This website based online analyses suites intrinsically utilized a large number of datasets of microarray gene expression data and clinical survival information from Gene Expression Omnibus (GEO) database^95^ to perform suvival analyses mainly on three types of cancers: breast cancer, ovarian cancer and lung cancer. Therefore, we utilized this tool to perform survival analyses of the Immu6Metagene signature on the above three types of cancers based on the microarray datasets internally available in KMPlotter. The corresponding Kaplan–Meier survival curves (Fig. 5) were also made by using KMPlotter online. We analyzed the Immu6Metagene signature as multigene classifier, which was defined by the expression values of the probesets for these six genes internally available in the KMPlotter program.

To identify the significant pathway activity changes between the low risk group with high Immu6Metagene expression and the high risk group with low Immu6Metagene expression, we applied the GSVA method^30^ and the Molecular Signatures Database (MSigDB) hallmark gene set collection^31,32^ to the analyses of the RNA-seq data of the TCGA cancer cohorts of BRCA (Breast invasive carcinoma), CESC (Cervical and endocervical cancers) and LUAD (Lung adenocarcinoma). The heatmaps of pathway activity pattern changes across the cancer patients were generated using the R package – heatmap3 (https://cran.r-project.org/web/packages/heatmap3/).

## Supplementary information

Supplemental_Information

## Data Availability

All the data used in this paper were publicly availabe datasets with corresponding website links given in the body text.
