# Supplementary material for: Tumor intrinsic immunity related proteins may be novel tumor suppressors in some types of cancer
Source: Sci Rep. 2019 Jul 29;9:10918. doi: 10.1038/s41598-019-47382-3 (PMC6662687; doi:10.1038/s41598-019-47382-3)
Supplement: Supplementary file 1 — Supplemental_Information [file 41598_2019_47382_MOESM1_ESM.pdf]

Tumor intrinsic immunity related proteins may be novel tumor suppressors in some types of cancer

Donghai Xiong[1], Yian Wang[1], and Ming You[1]

SUPPLEMENTARY FIGURES:

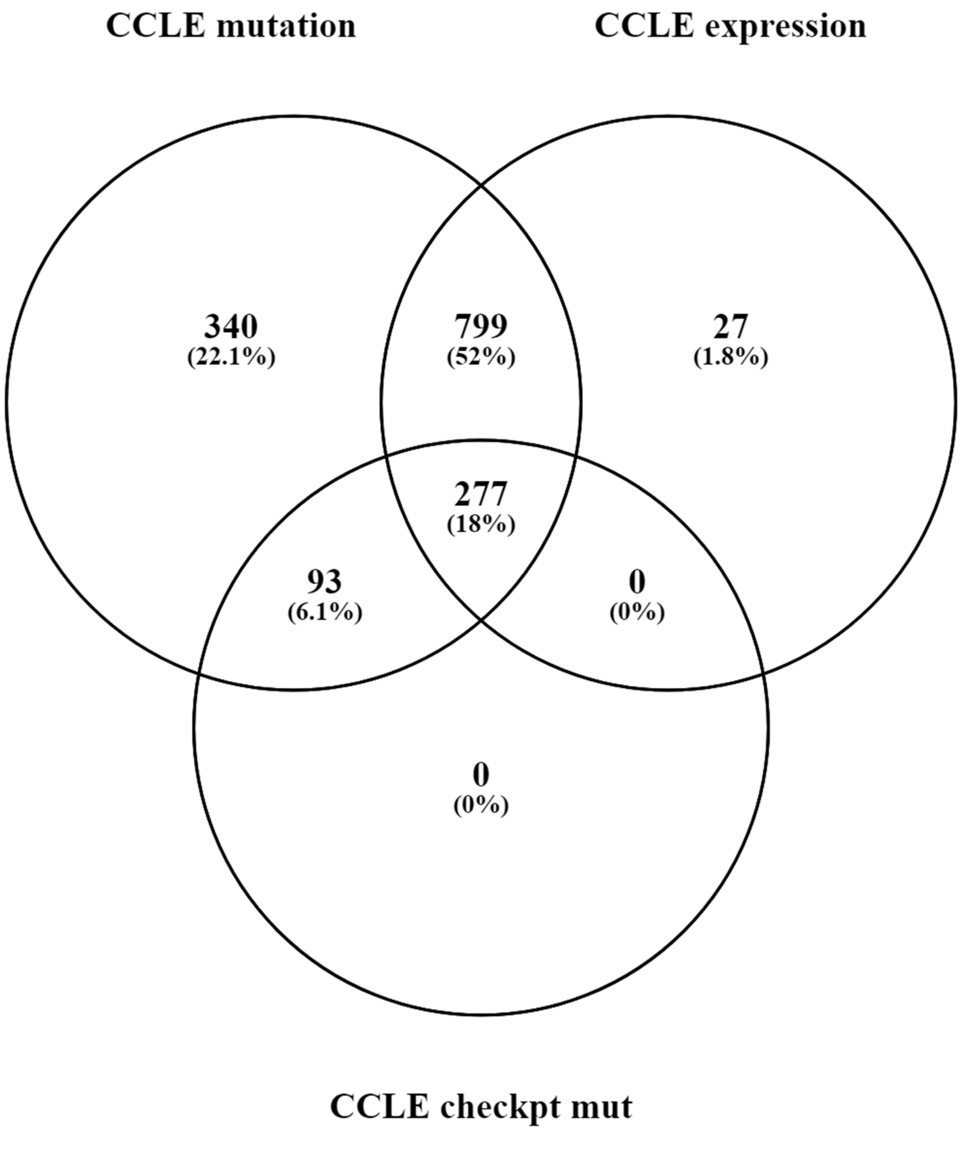

Figure S1. Venn diagram showed the numbers of the CCLE cell lines with mutation data available ("CCLE mutation" circle), the CCLE cell lines with expression data available ("CCLE expression" circle), and the CCLE cell lines having nonsilent somatic mutations in the 31 immune checkpoint genes ("CCLE checkpt mut" circle). The overlapping of these datasets were shown in this Venn

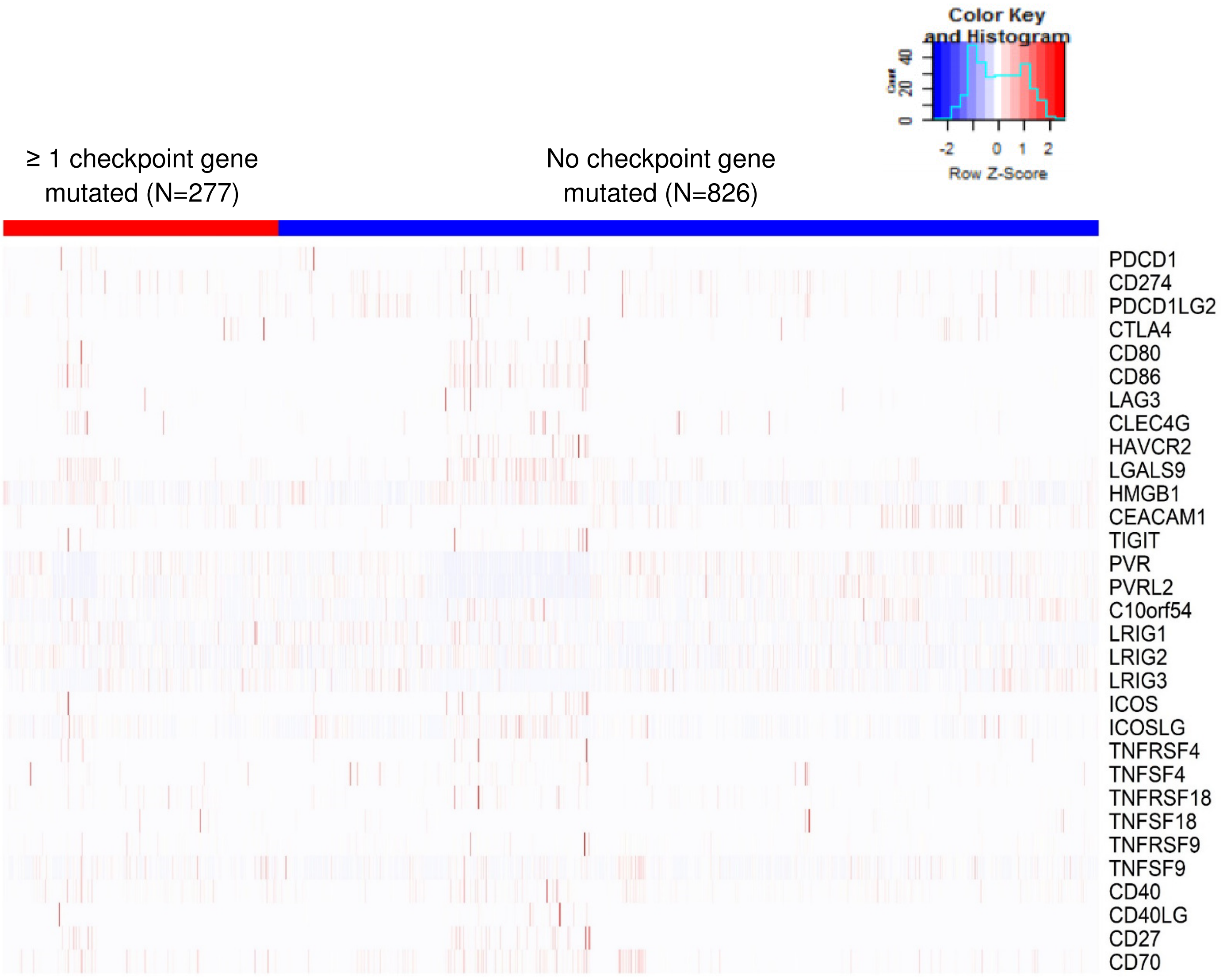

Figure S2. Expression profiling of the immune checkpoint genes in the CCLE cancer cell lines. Overall, the 1103 CCLE cancer cell lines from 22 tissues had low expression in the set of 31 immune checkpoint genes. The expression of the individual gene in this gene set did not change significantly according to the the overall nonsilent somatic mutation status of the set of 31 immune checkpoint genes (mutation group vs non-mutation group, adjusted P value > 0.1 for each of the 31 genes).

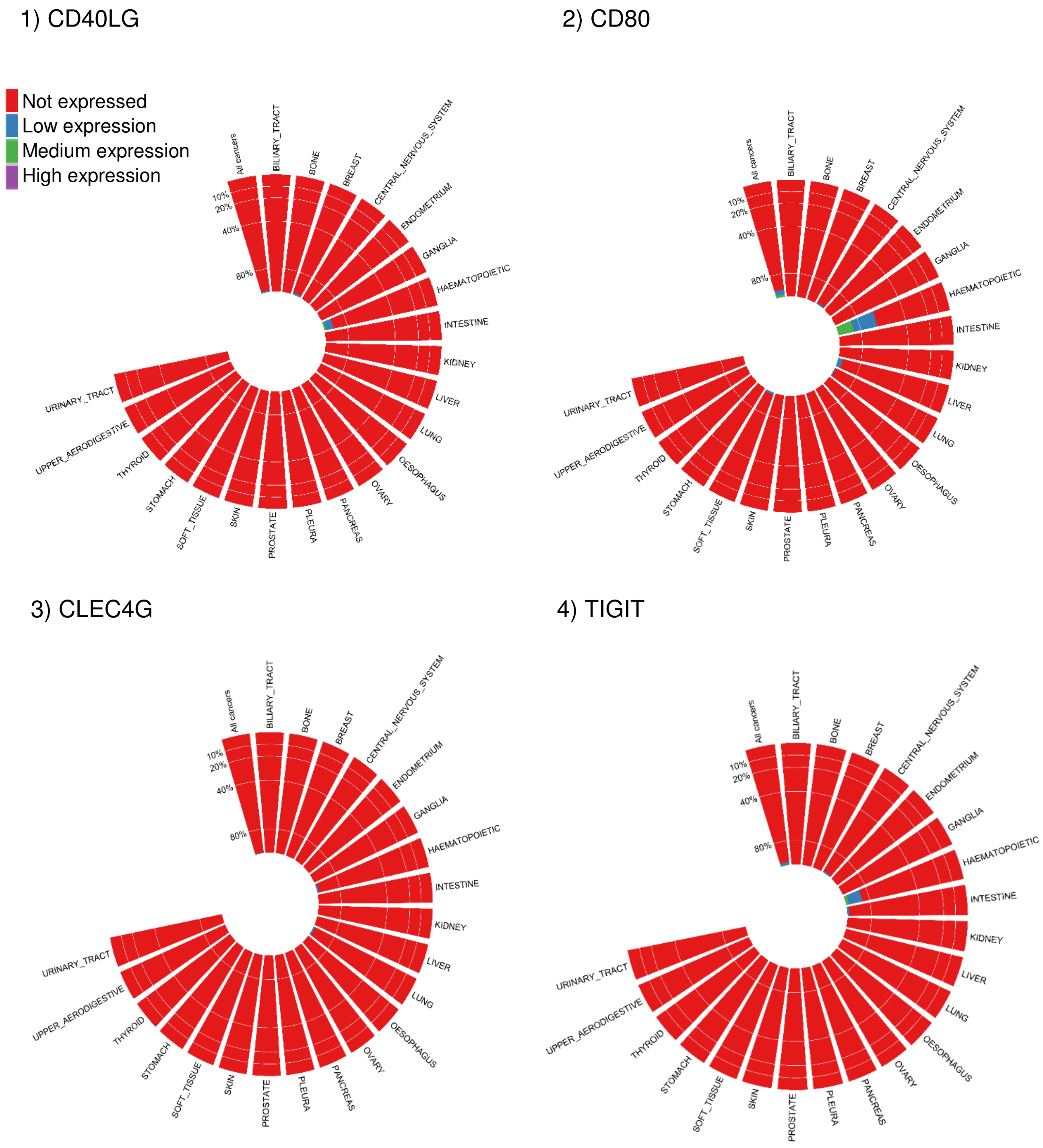

Figure S3. The immune checkpoint genes that were not expressed across almost all the CCLE cancer cell lines. These included 6 genes - CD40LG, CD80, CLEC4G, TIGIT, TNFRSF4, ICOS, the polar histograms of which were shown in 1) to 6).

5) TNFRSF4

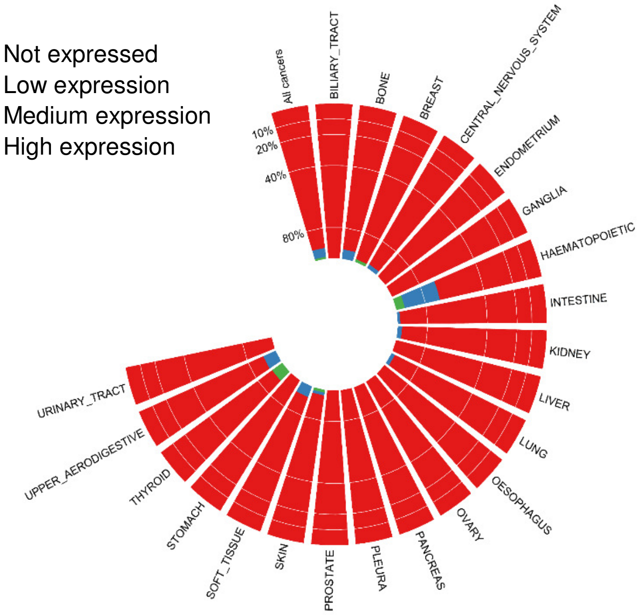

6) ICOS

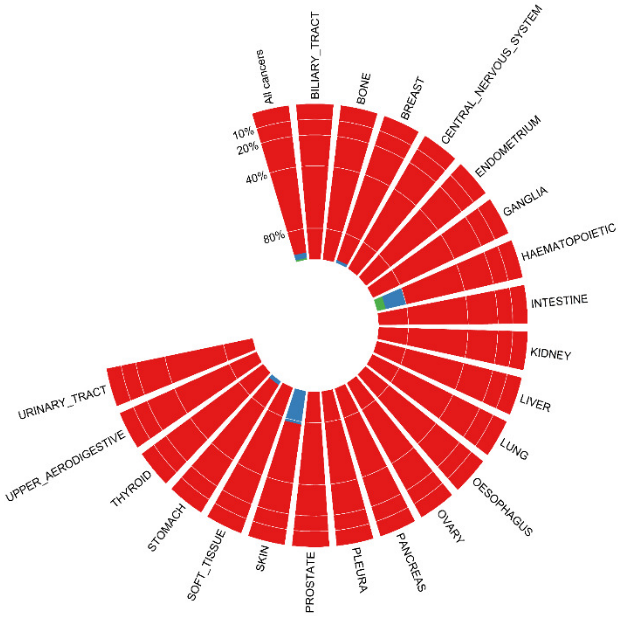

Figure S3 (continuing). The immune checkpoint genes that were not expressed across almost all the CCLE cancer cell lines. These included 6 genes - CD40LG, CD80, CLEC4G, TIGIT, TNFRSF4, ICOS, the polar histograms of which were shown in 1) to 6).

1) C10orf54 (VISTA)

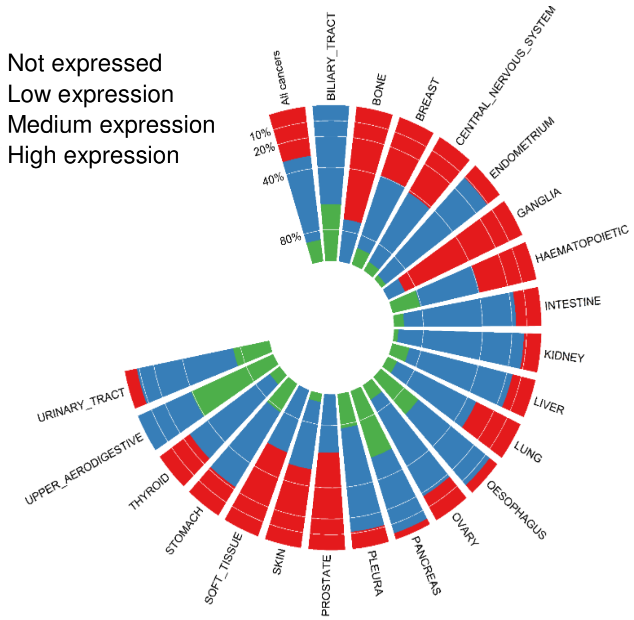

2) CD27

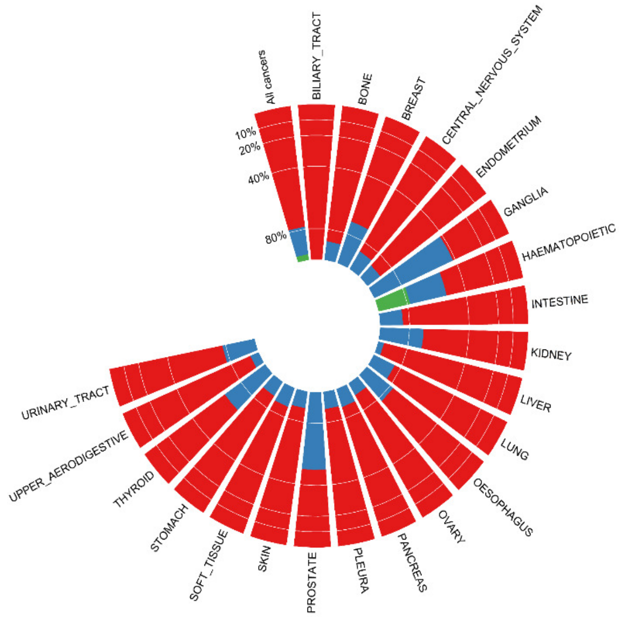

3) CD40

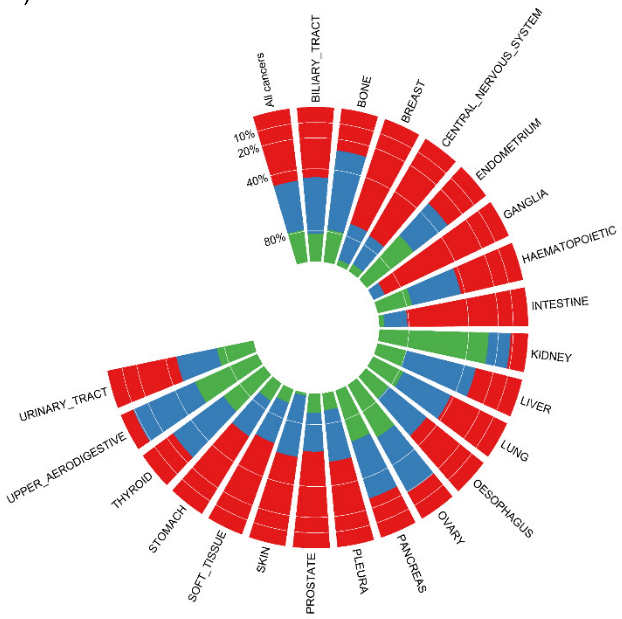

4) CD86

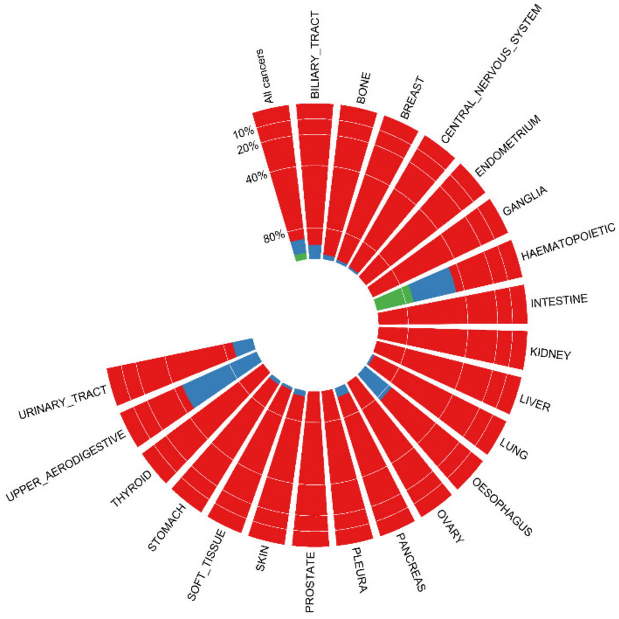

Figure S4. The immune checkpoint genes that were not expressed or having low expression in most cancer cell lines across the 22 distinct types of tissues. These included 22 genes - C10orf54 (VISTA), CD27, CD40, CD86, CD274 (PD-L1), CEACAM1, CTLA4, HAVCR2 (TIM3), CD70, ICOSLG, LAG3, LGALS9, LRIG1, LRIG2, LRIG3, PDCD1 (PD-1), PDCD1LG2 (PD-L2), TNFRSF9, TNFRSF18, TNFSF4, TNFSF9, TNFSF18, the polar histograms of which were shown in 1) to 22).

5) CD274 (PD-L1)

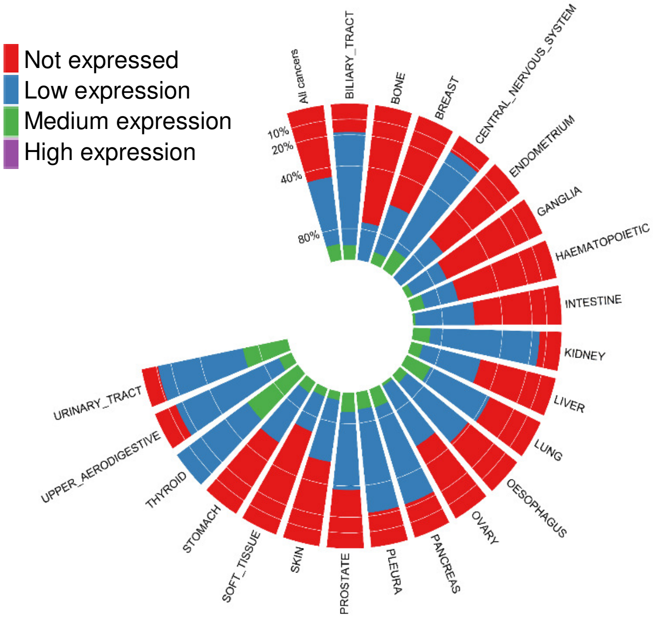

6) CEACAM1

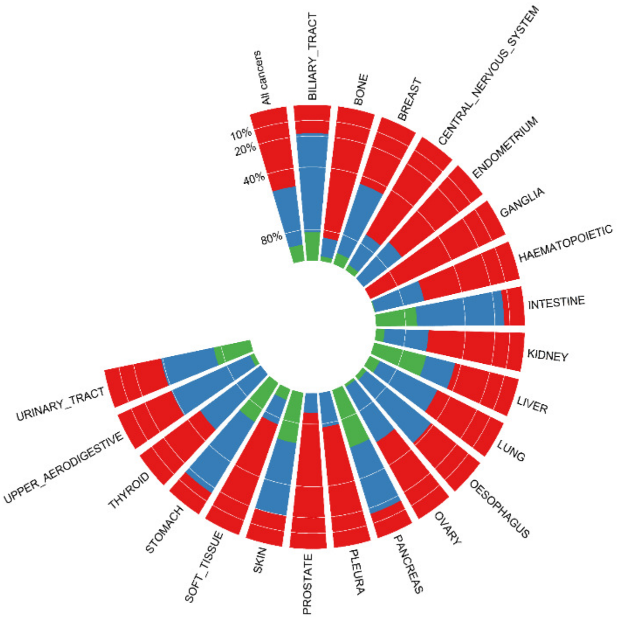

7) CTLA4

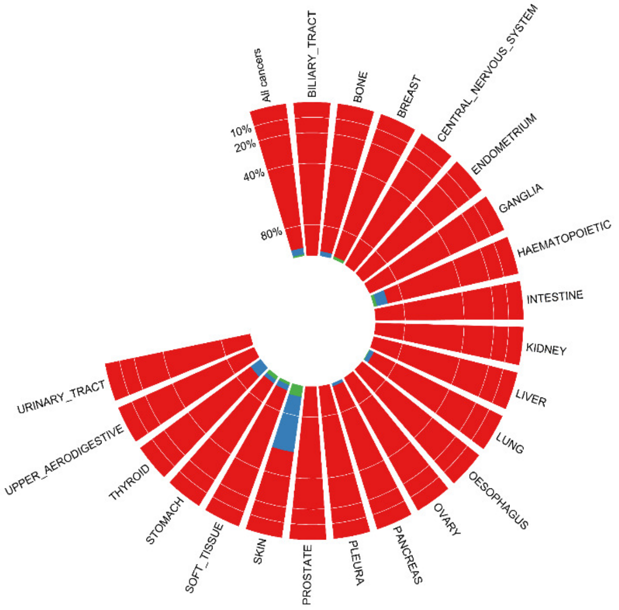

8) HAVCR2 (TIM3)

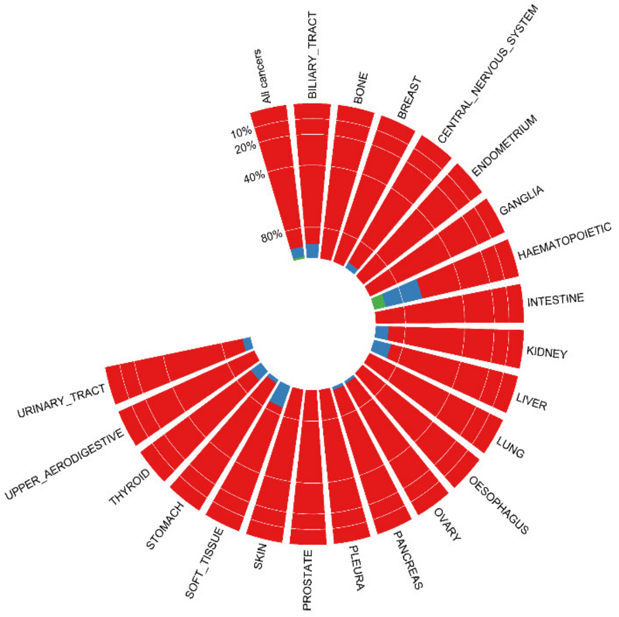

Figure S4 (continuing). The immune checkpoint genes that were not expressed or having low expression in most cancer cell lines across the 22 distinct types of tissues. These included 22 genes - C10orf54 (VISTA), CD27, CD40, CD86, CD274 (PD-L1), CEACAM1, CTLA4, HAVCR2 (TIM3), CD70, ICOSLG, LAG3, LGALS9, LRIG1, LRIG2, LRIG3, PDCD1 (PD-1), PDCD1LG2 (PD-L2), TNFRSF9, TNFRSF18, TNFSF4, TNFSF9, TNFSF18, the polar histograms of which were shown in 1) to 22).

9) CD70

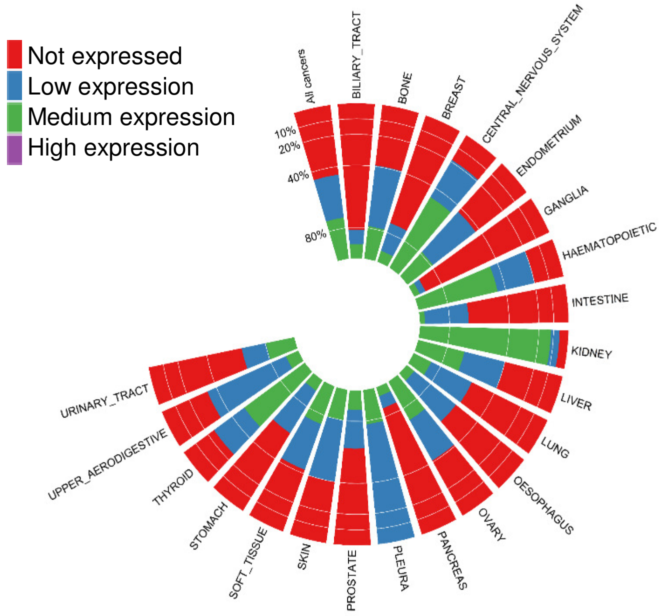

10) ICOSLG

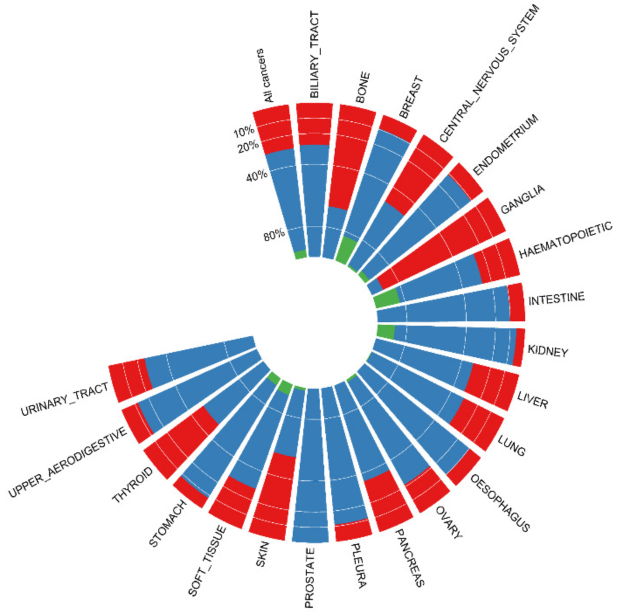

11) LAG3

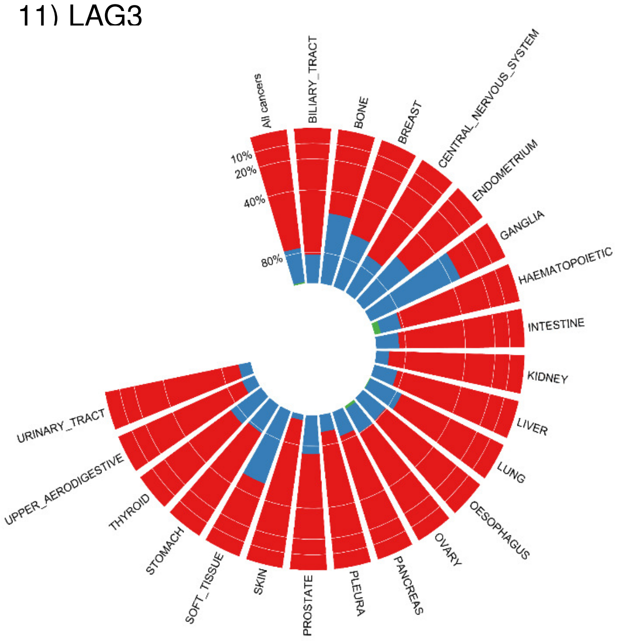

12) LGALS9

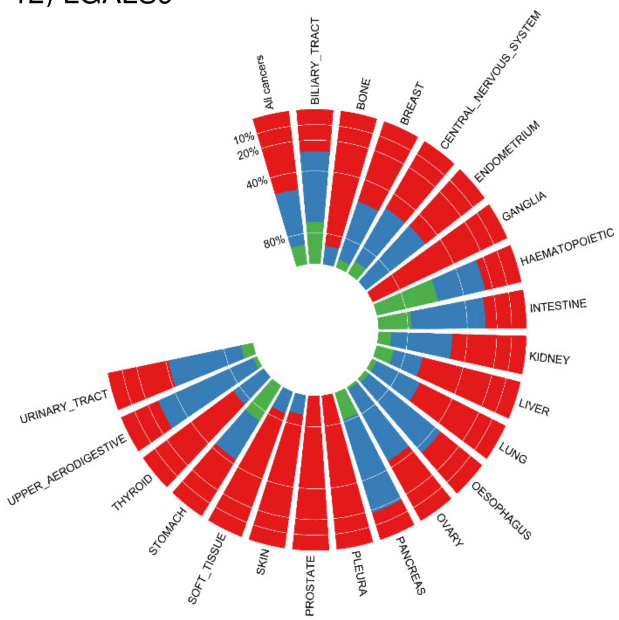

Figure S4 (continuing). The immune checkpoint genes that were not expressed or having low expression in most cancer cell lines across the 22 distinct types of tissues. These included 22 genes - C10orf54 (VISTA), CD27, CD40, CD86, CD274 (PD-L1), CEACAM1, CTLA4, HAVCR2 (TIM3), CD70, ICOSLG, LAG3, LGALS9, LRIG1, LRIG2, LRIG3, PDCD1 (PD-1), PDCD1LG2 (PD-L2), TNFRSF9, TNFRSF18, TNFSF4, TNFSF9, TNFSF18, the polar histograms of which were shown in 1) to 22).

13) LRIG1

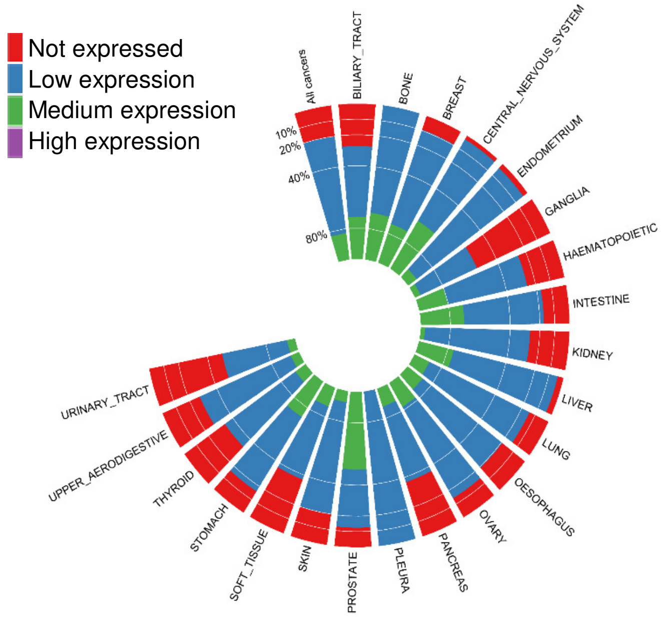

14) LRIG2

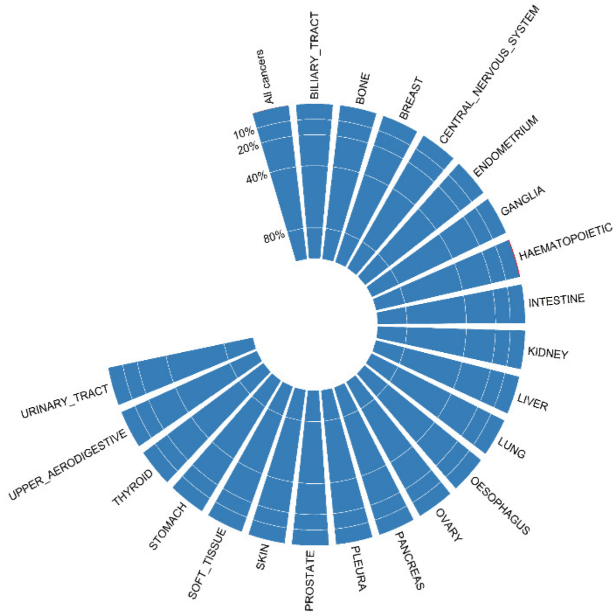

15) LRIG3

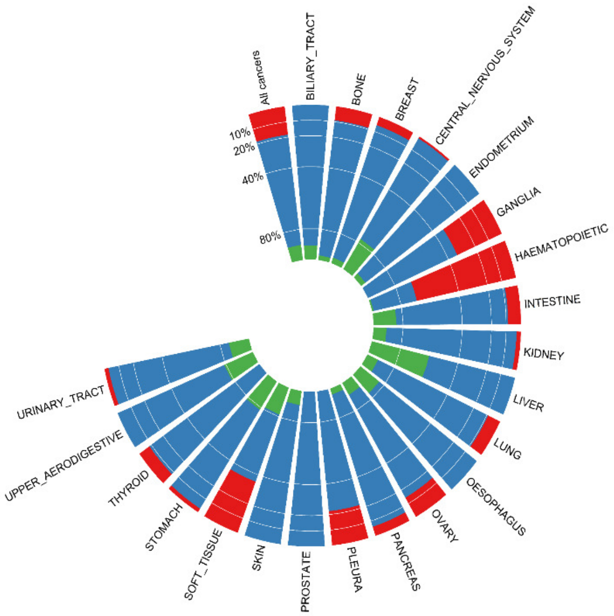

16) PDCD1 (PD-1)

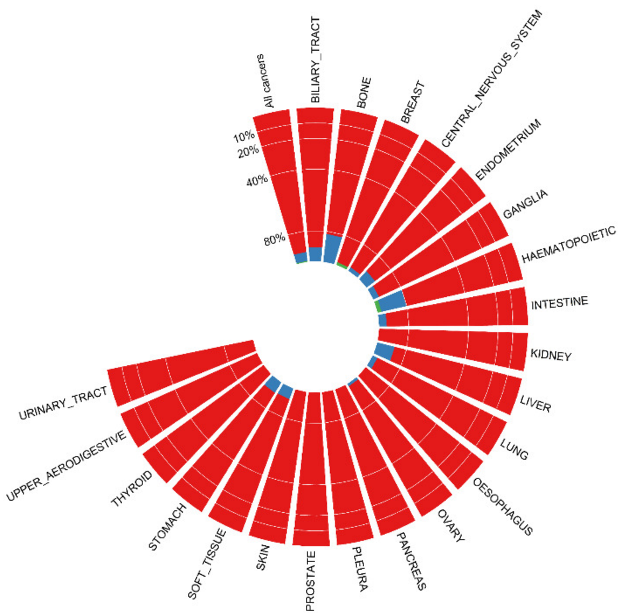

Figure S4 (continuing). The immune checkpoint genes that were not expressed or having low expression in most cancer cell lines across the 22 distinct types of tissues. These included 22 genes - C10orf54 (VISTA), CD27, CD40, CD86, CD274 (PD-L1), CEACAM1, CTLA4, HAVCR2 (TIM3), CD70, ICOSLG, LAG3, LGALS9, LRIG1, LRIG2, LRIG3, PDCD1 (PD-1), PDCD1LG2 (PD-L2), TNFRSF9, TNFRSF18, TNFSF4, TNFSF9, TNFSF18, the polar histograms of which were shown in 1) to 22).

17) PDCD1LG2 (PD-L2)

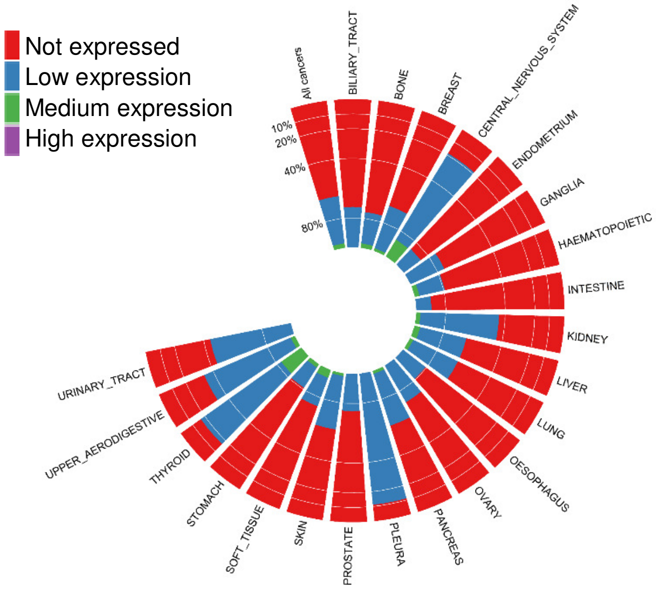

18) TNFRSF9

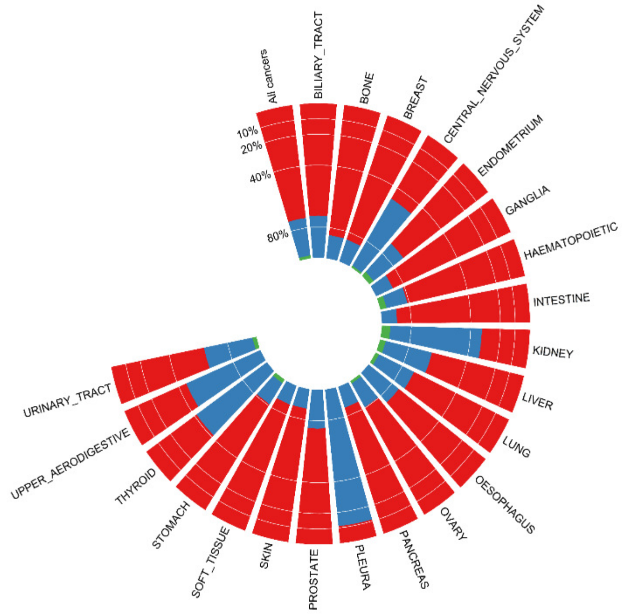

19) TNFRSF18

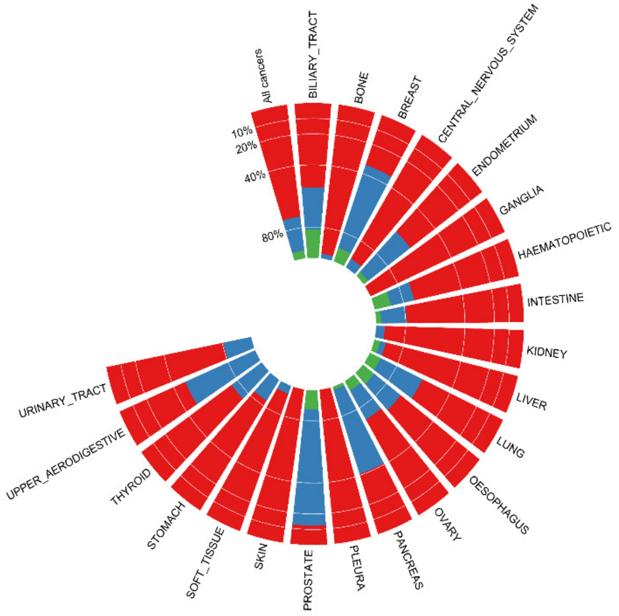

20) TNFSF4

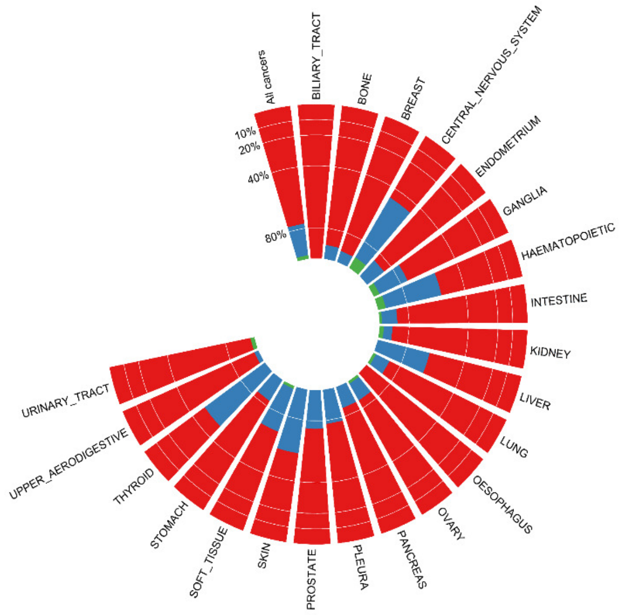

Figure S4 (continuing). The immune checkpoint genes that were not expressed or having low expression in most cancer cell lines across the 22 distinct types of tissues. These included 22 genes - C10orf54 (VISTA), CD27, CD40, CD86, CD274 (PD-L1), CEACAM1, CTLA4, HAVCR2 (TIM3), CD70, ICOSLG, LAG3, LGALS9, LRIG1, LRIG2, LRIG3, PDCD1 (PD-1), PDCD1LG2 (PD-L2), TNFRSF9, TNFRSF18, TNFSF4, TNFSF9, TNFSF18, the polar histograms of which were shown in 1) to 22).

21) TNFSF9

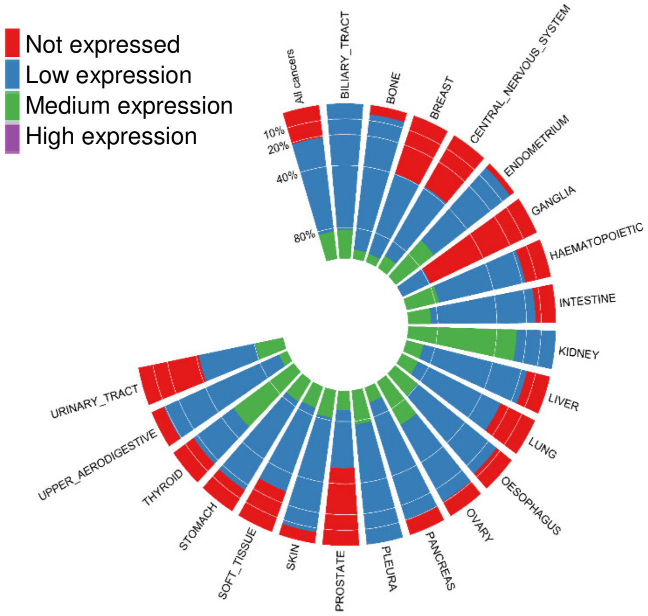

22) TNFSF18

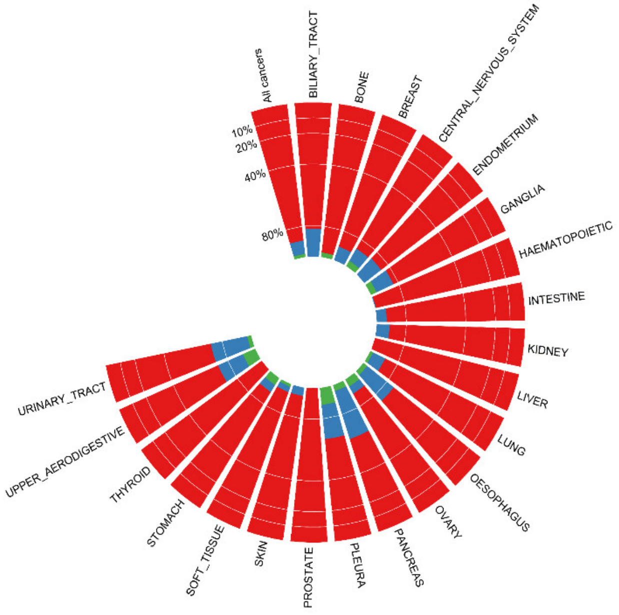

Figure S4 (continuing). The immune checkpoint genes that were not expressed or having low expression in most cancer cell lines across the 22 distinct types of tissues. These included 22 genes - C10orf54 (VISTA), CD27, CD40, CD86, CD274 (PD-L1), CEACAM1, CTLA4, HAVCR2 (TIM3), CD70, ICOSLG, LAG3, LGALS9, LRIG1, LRIG2, LRIG3, PDCD1 (PD-1), PDCD1LG2 (PD-L2), TNFRSF9, TNFRSF18, TNFSF4, TNFSF9, TNFSF18, the polar histograms of which were shown in 1) to 22).

1) HMGB1

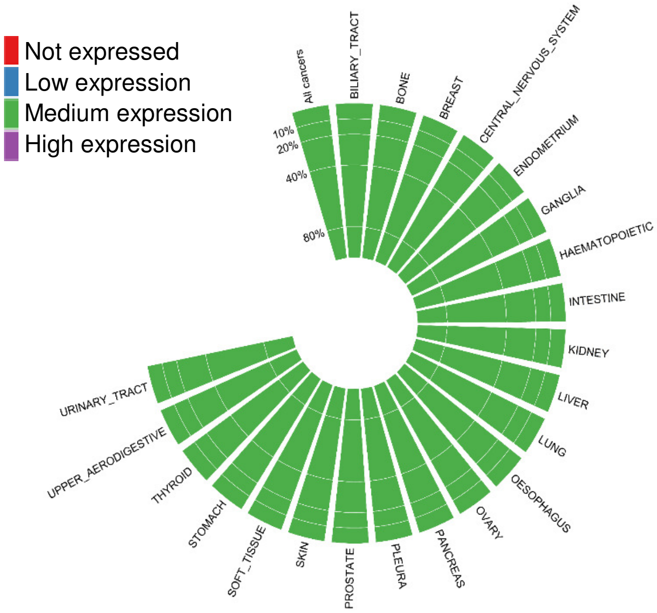

2) PVR

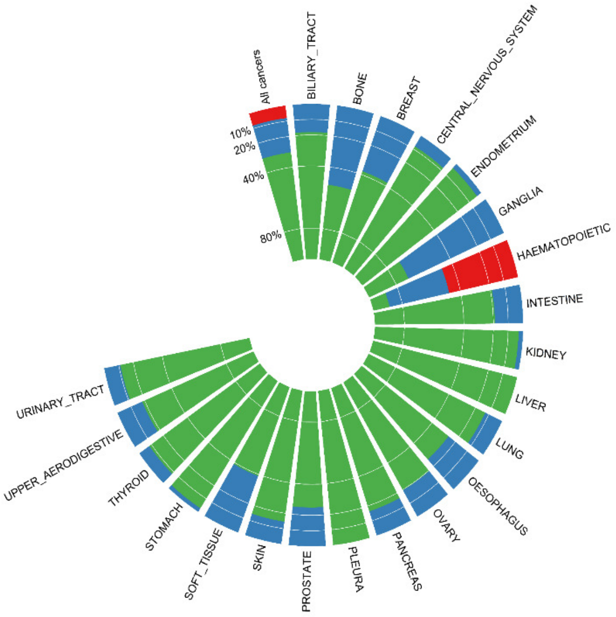

3) PVRL2 (NECTIN2)

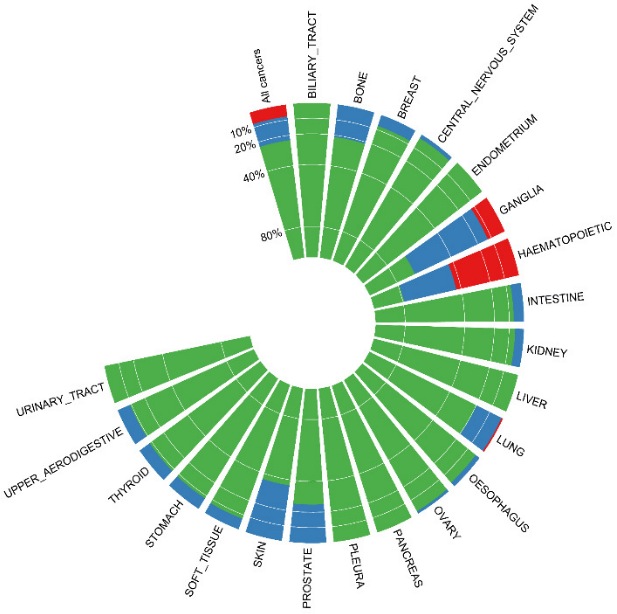

Figure S5. The immune checkpoint genes that had medium expression in most cancer cell lines across the 22 distinct types of tissues. These included 3 genes – HMGB1, PVR, PVRL2 (NECTIN2), the polar histograms of which were shown in 1) to 3).

1) CTLA4

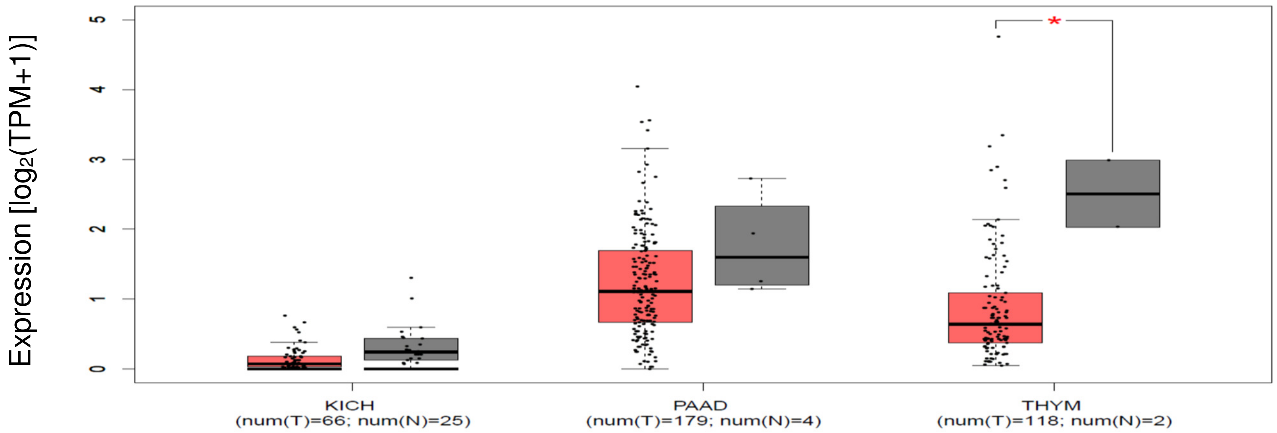

2) CD80 (B7-1)

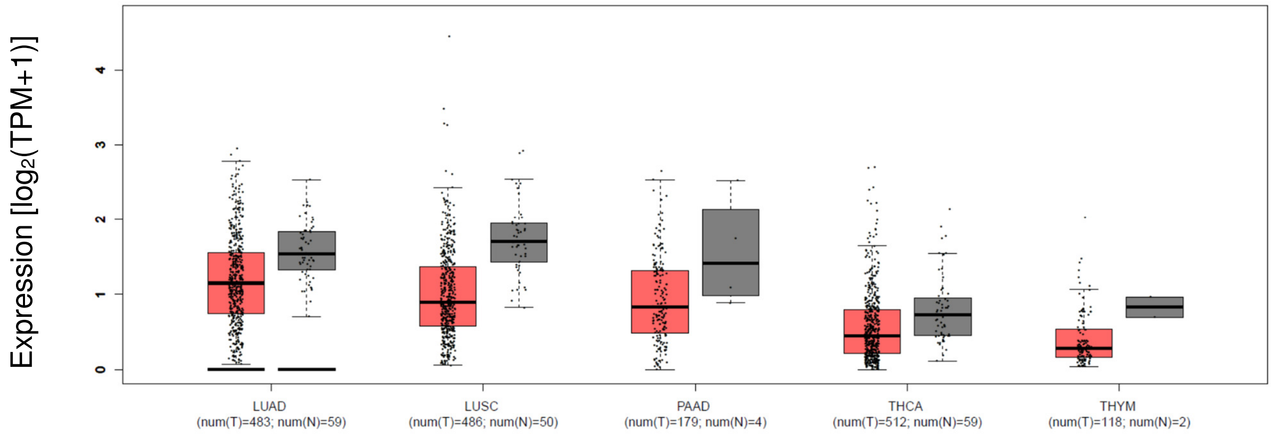

3) CD86 (B7-2)

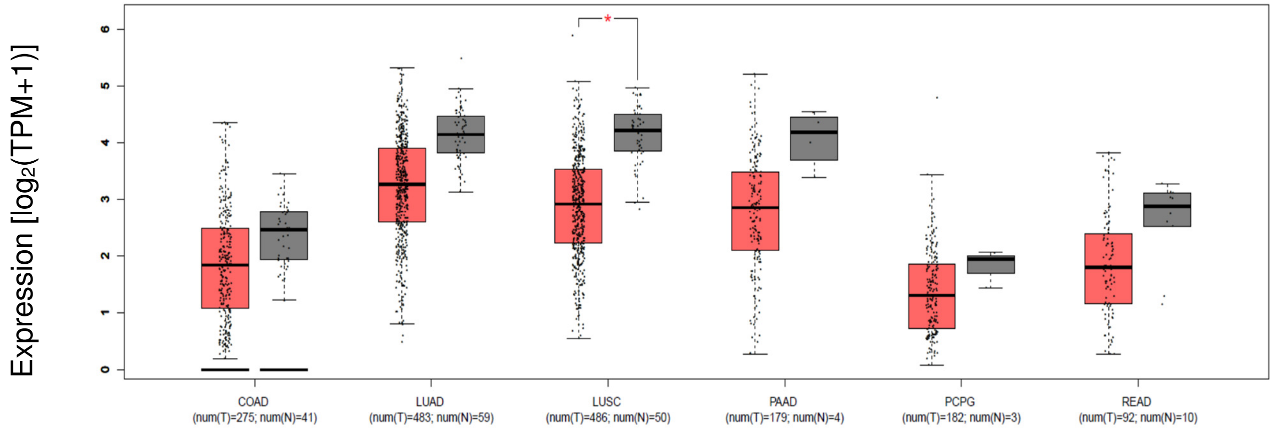

Figure S6. The expressions of the 28 genes from no/low expression groups in cancer cell lines were down-regulated in the tumor samples compared to the normal tissues across different TCGA cancer types. Expression values were given as $\log_2$(TPM + 1) for log-scale. Red boxes denote tumor samples, black boxes denote normal samples.

4) PDCD1 (PD-1)

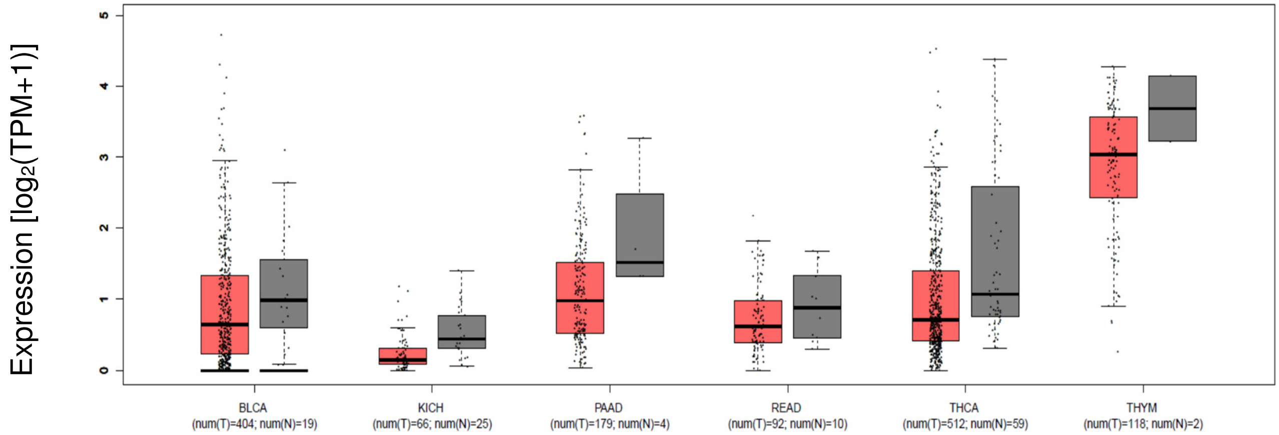

5) CD274 (PD-L1)

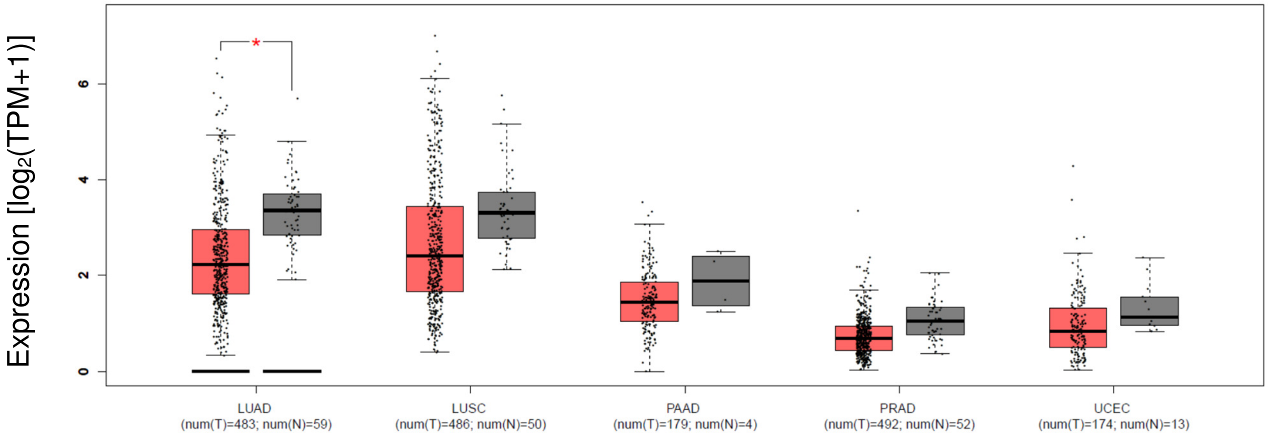

6) PDCD1LG2 (PD-L2)

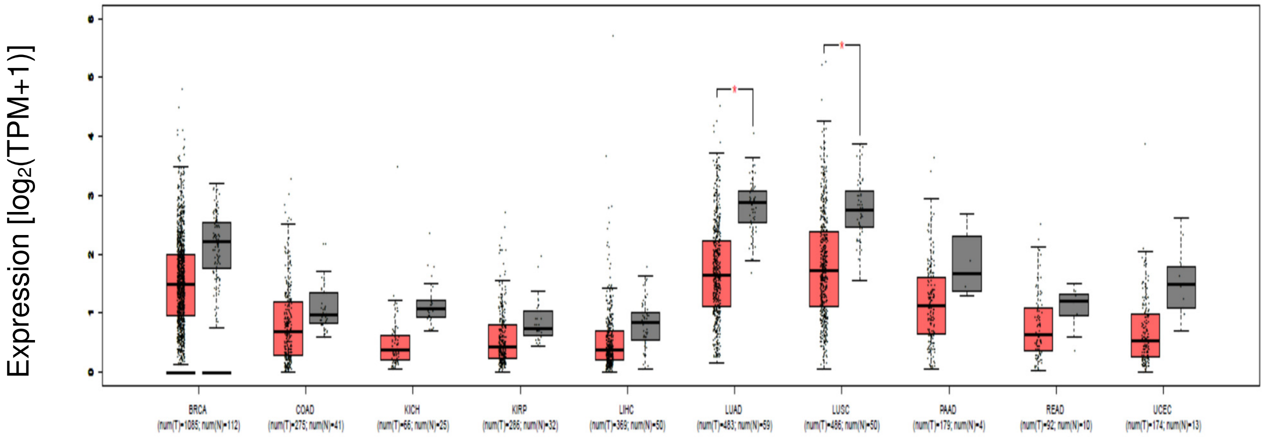

Figure S6. The expressions of the 28 genes from no/low expression groups in cancer cell lines were down-regulated in the tumor samples compared to the normal tissues across different TCGA cancer types. Expression values were given as $\log_2$(TPM + 1) for log-scale. Red boxes denote tumor samples, black boxes denote normal samples.

7) LAG3

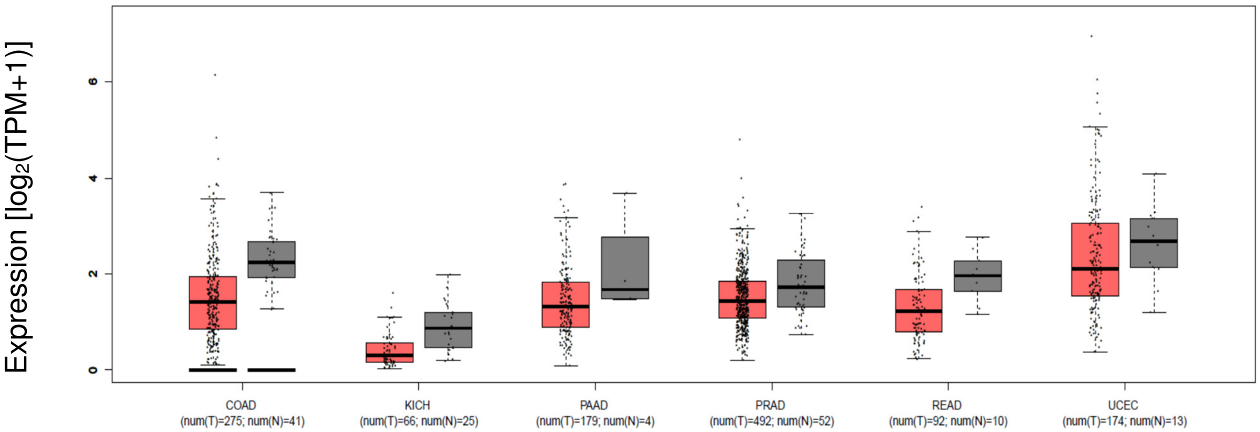

8) CLEC4G (LSECtin)

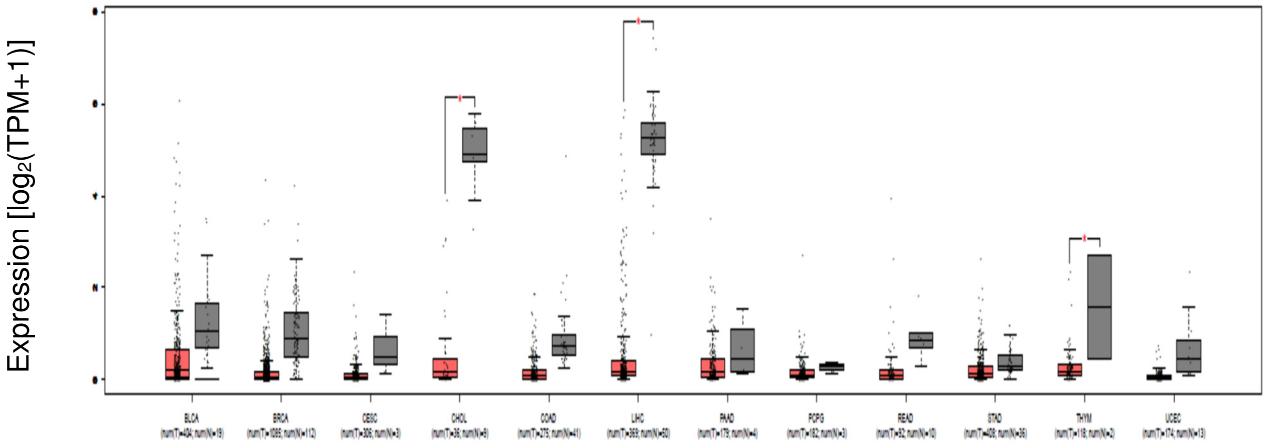

9) HAVCR2 (TIM3)

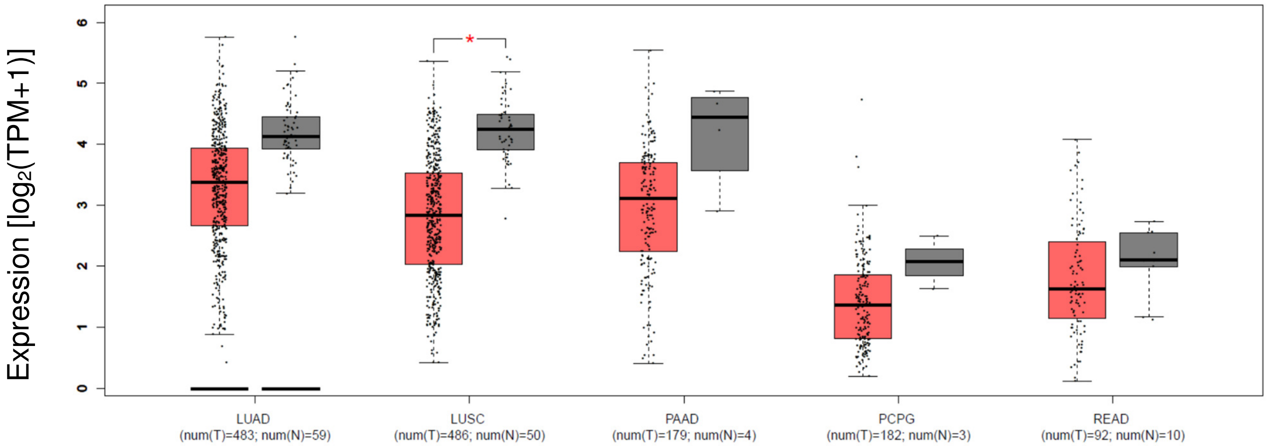

Figure S6. The expressions of the 28 genes from no/low expression groups in cancer cell lines were down-regulated in the tumor samples compared to the normal tissues across different TCGA cancer types. Expression values were given as log_2(TPM + 1) for log-scale. Red boxes denote tumor samples, black boxes denote normal samples.

10) LGALS9

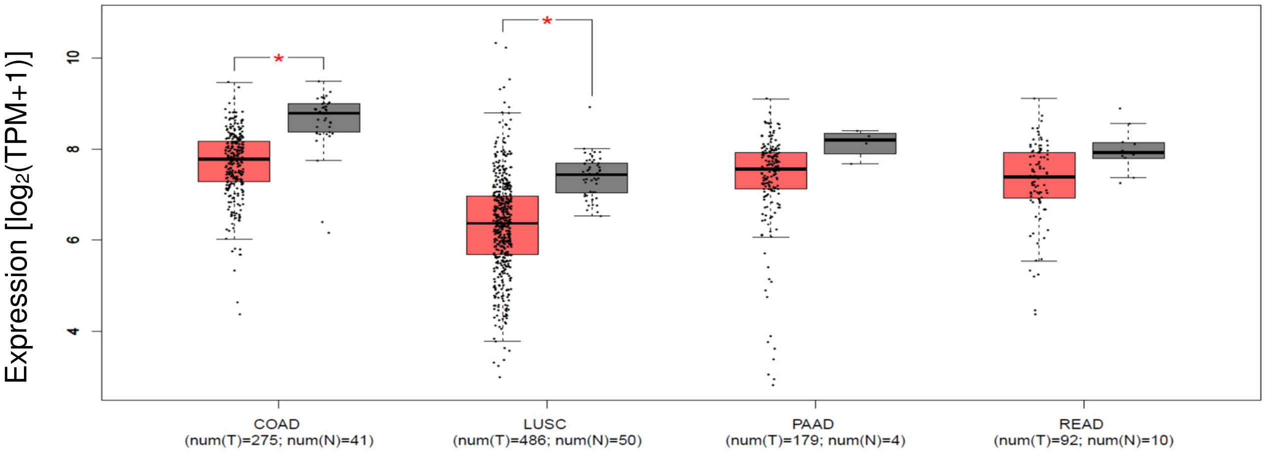

11) CEACAM1

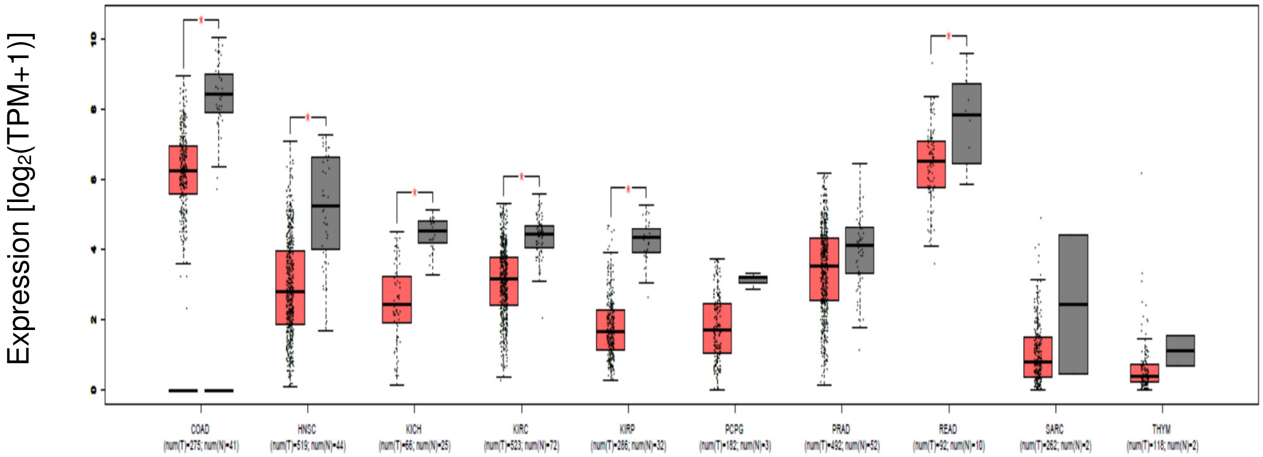

12) TIGIT

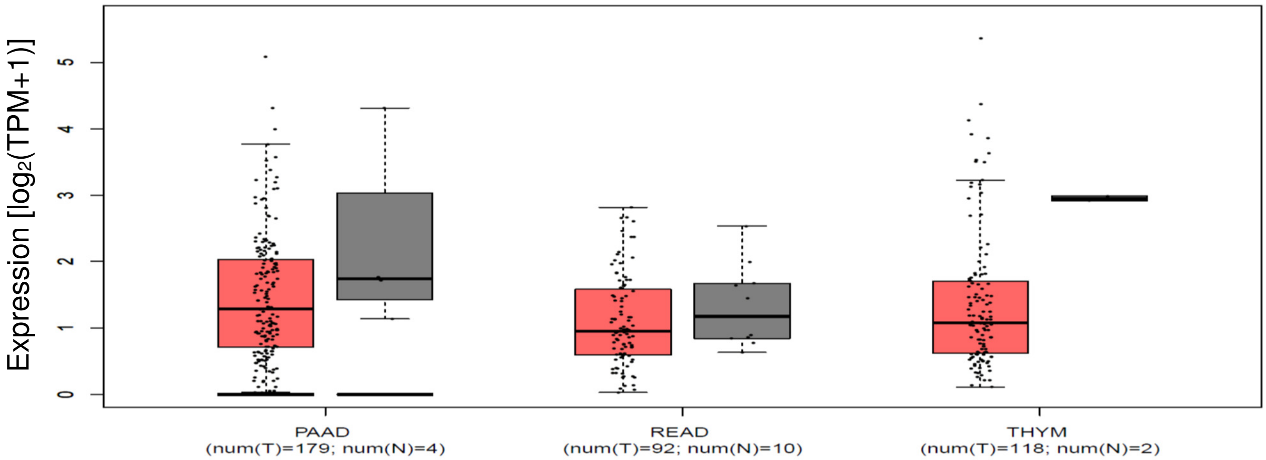

Figure S6. The expressions of the 28 genes from no/low expression groups in cancer cell lines were down-regulated in the tumor samples compared to the normal tissues across different TCGA cancer types. Expression values were given as $\log_2(\text{TPM} + 1)$ for log-scale. Red boxes denote tumor samples, black boxes denote normal samples.

13) VISTA (VSIR, C10orf54)

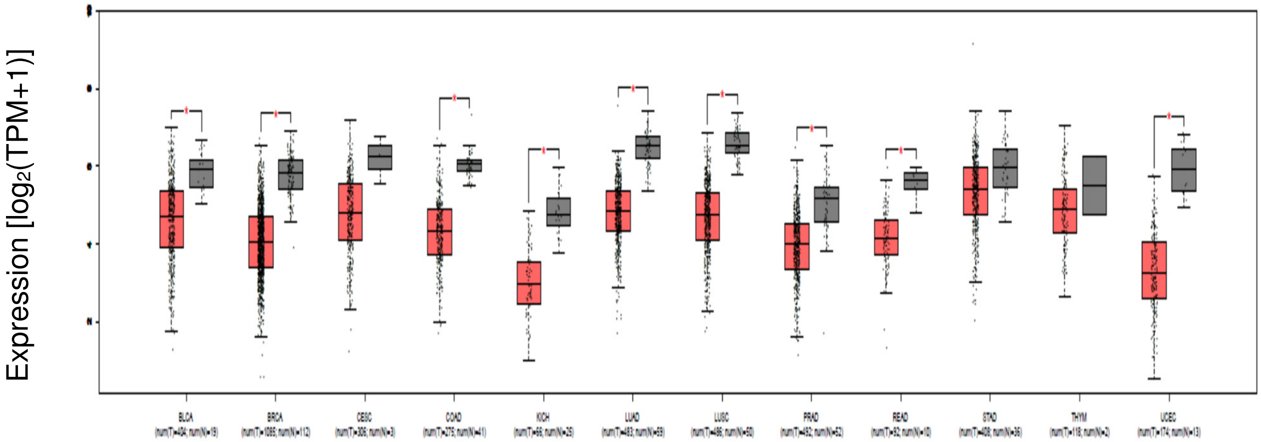

14) LRIG1

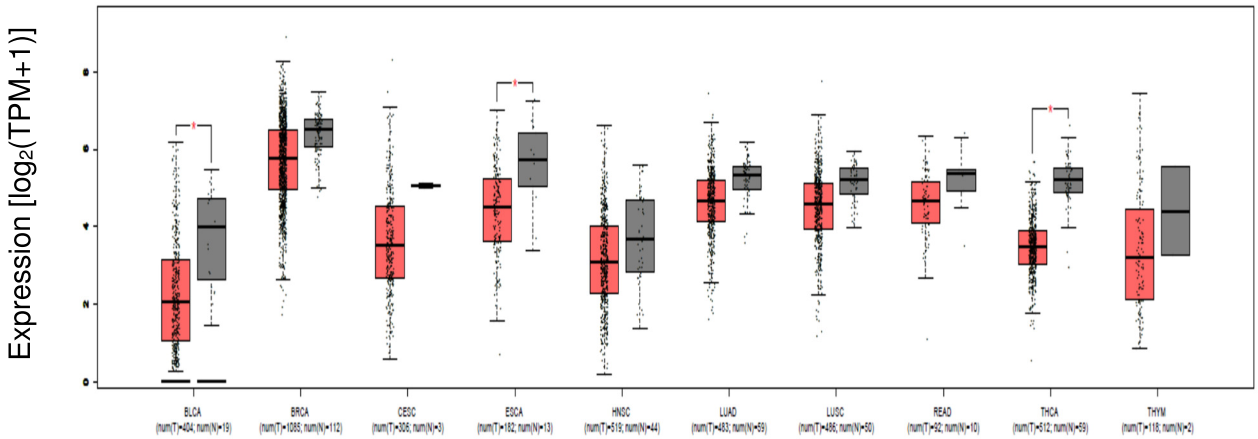

15) LRIG2

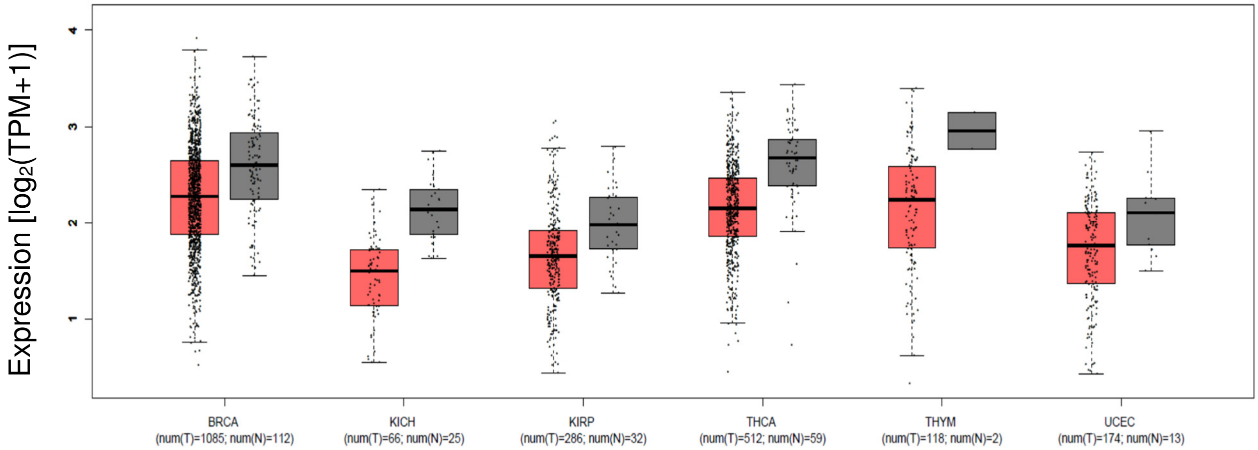

Figure S6. The expressions of the 28 genes from no/low expression groups in cancer cell lines were down-regulated in the tumor samples compared to the normal tissues across different TCGA cancer types. Expression values were given as $\log_2(TPM + 1)$ for log-scale. Red boxes denote tumor samples, black boxes denote normal samples.

16) LRIG3

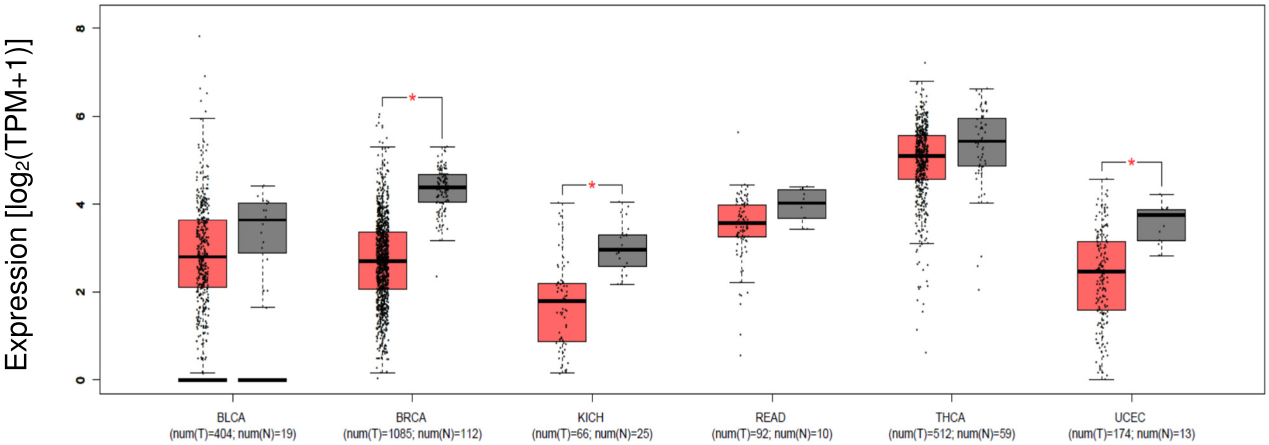

17) ICOS

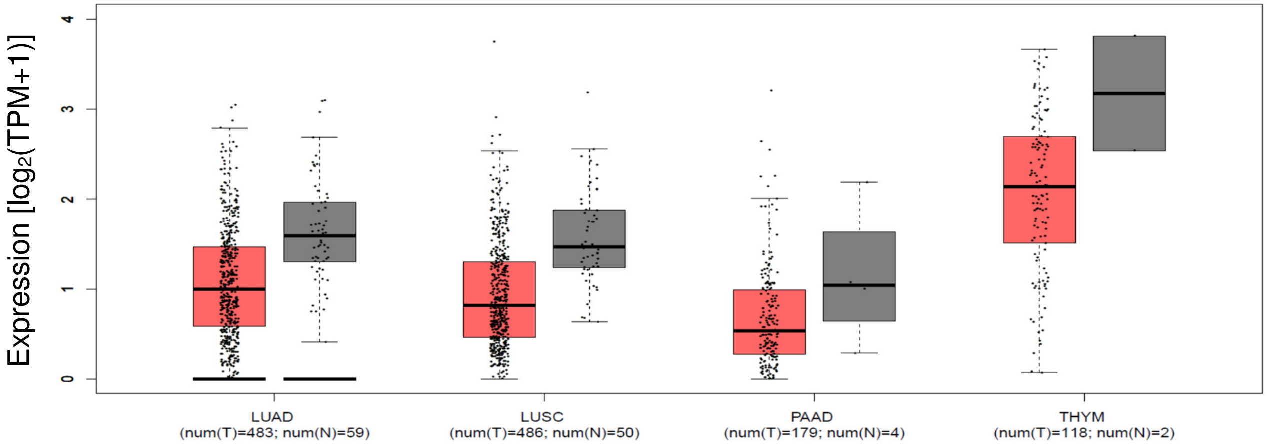

18) ICOSLG

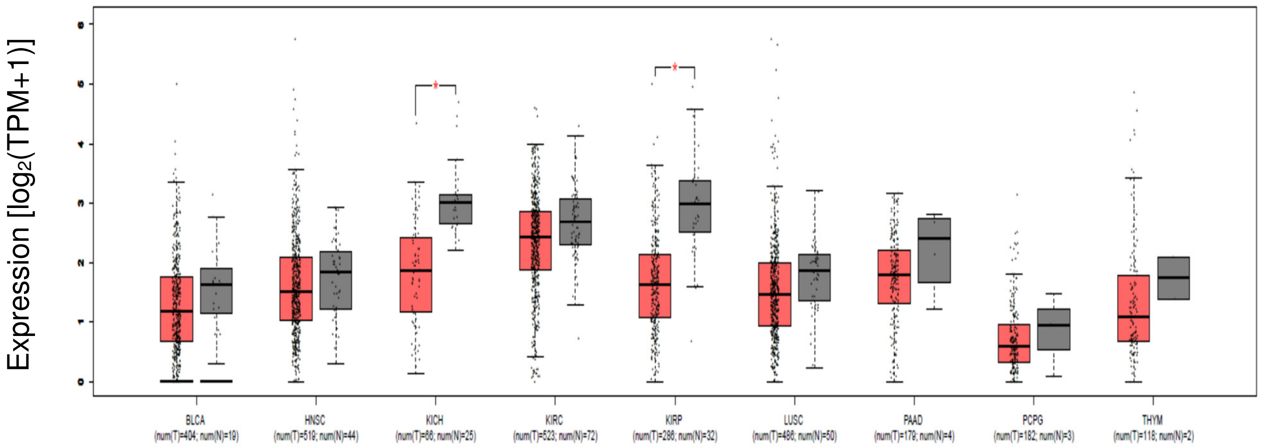

Figure S6. The expressions of the 28 genes from no/low expression groups in cancer cell lines were down-regulated in the tumor samples compared to the normal tissues across different TCGA cancer types. Expression values were given as $\log_2$(TPM + 1) for log-scale. Red boxes denote tumor samples, black boxes denote normal samples.

19) OX40 (TNFRSF4)

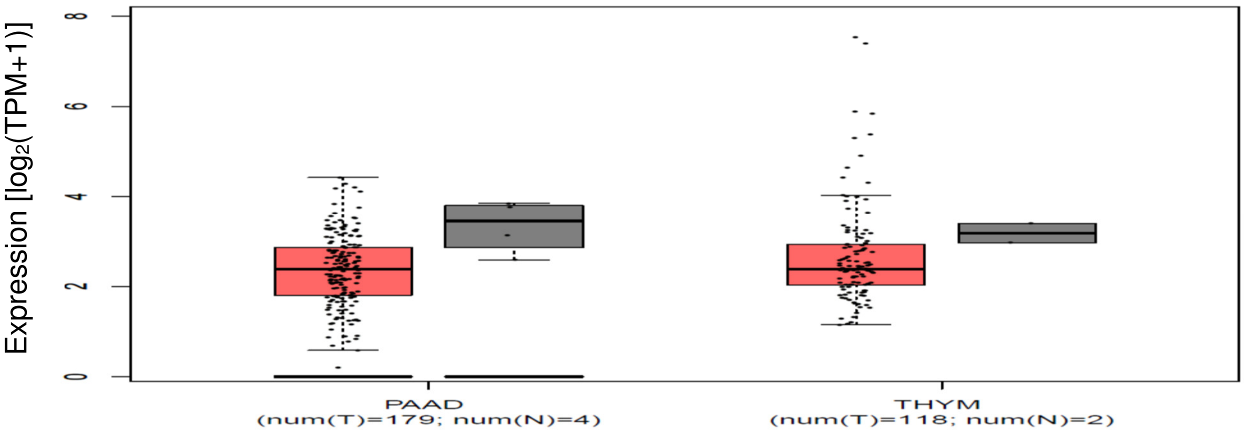

20) OX40L (TNFSF4)

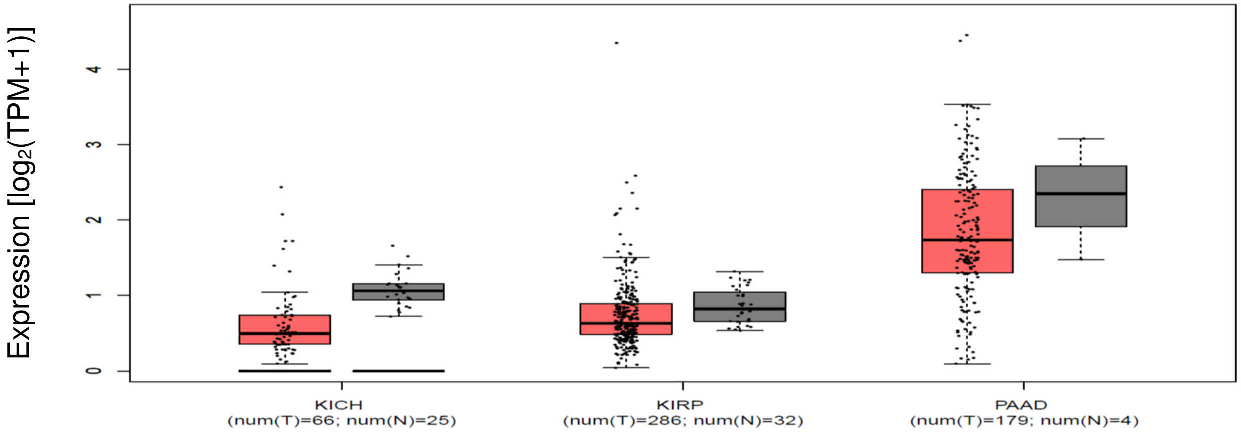

21) GITR (TNFRSF18)

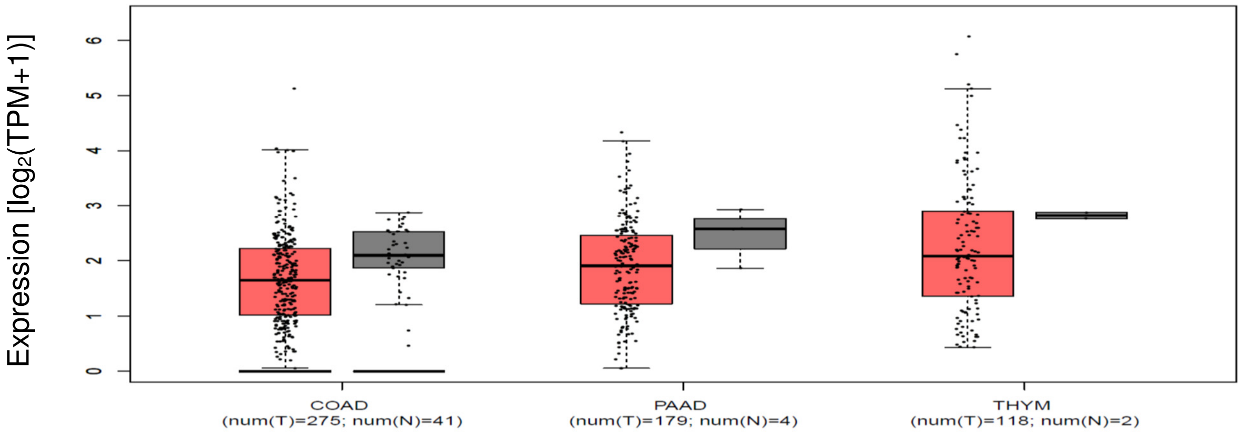

Figure S6. The expressions of the 28 genes from no/low expression groups in cancer cell lines were down-regulated in the tumor samples compared to the normal tissues across different TCGA cancer types. Expression values were given as log_2(TPM + 1) for log-scale. Red boxes denote tumor samples, black boxes denote normal samples.

22) GITRL (TNFSF18)

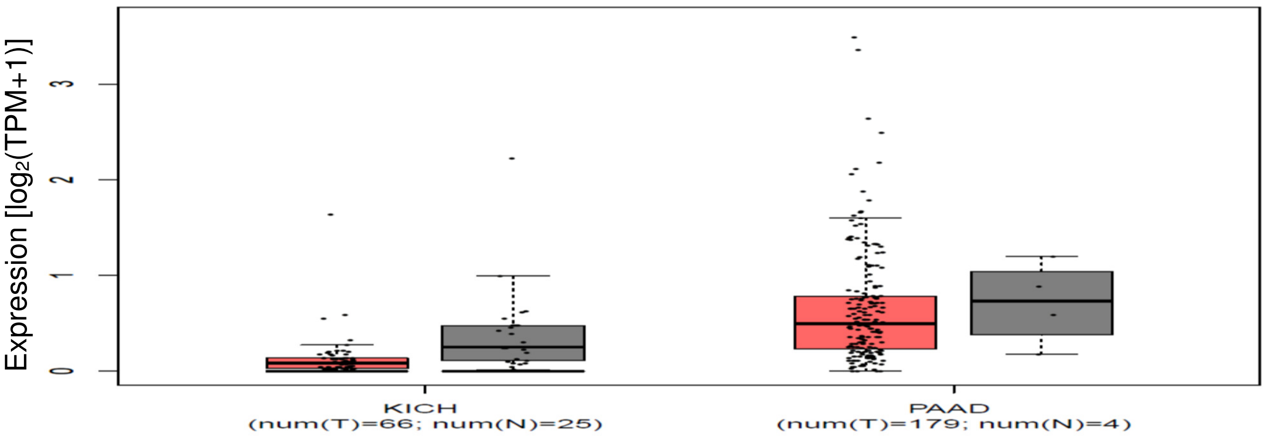

23) 4-1BB (TNFRSF9)

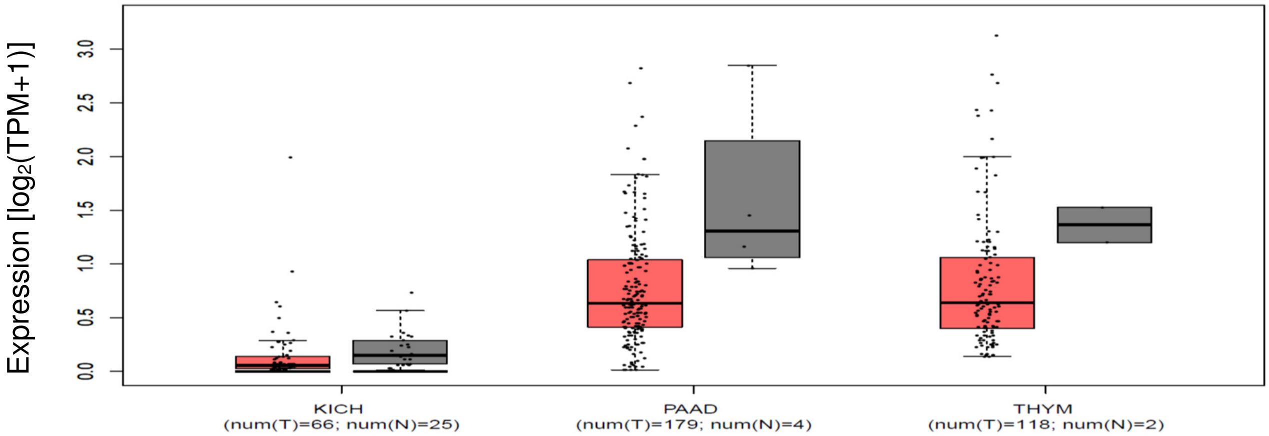

24) 4-1BBL (TNFSF9)

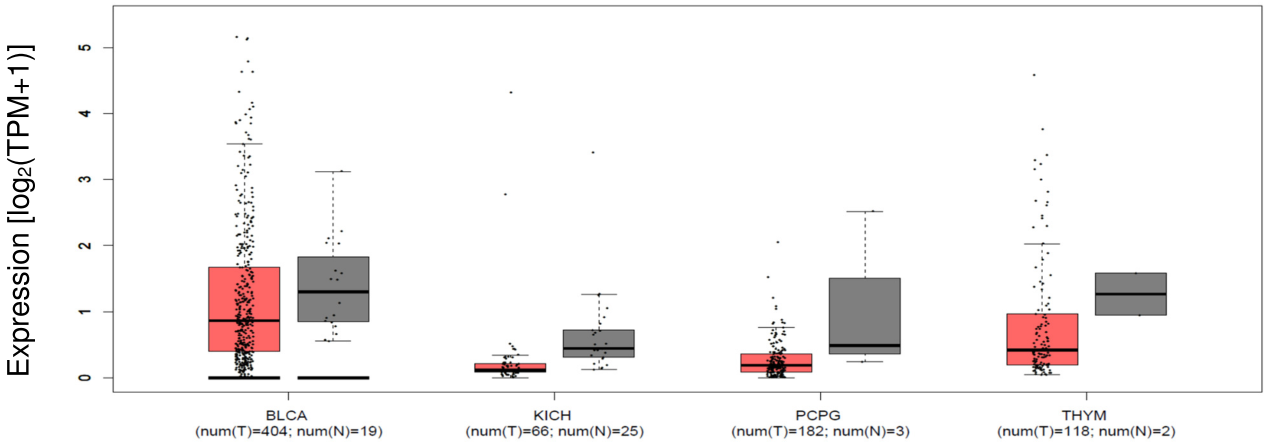

Figure S6. The expressions of the 28 genes from no/low expression groups in cancer cell lines were down-regulated in the tumor samples compared to the normal tissues across different TCGA cancer types. Expression values were given as log_2(TPM + 1) for log-scale. Red boxes denote tumor samples, black boxes denote normal samples.

25) CD40 (TNFRSF5)

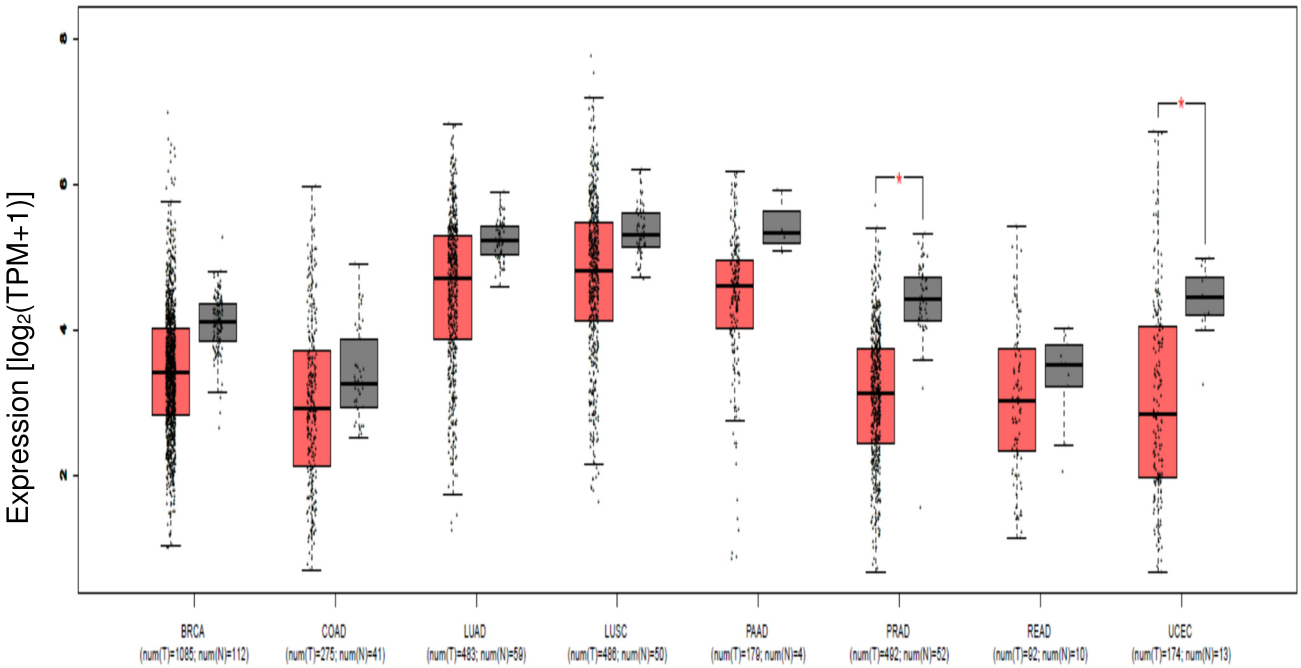

26) CD40L (CD40LG, TNFSF5)

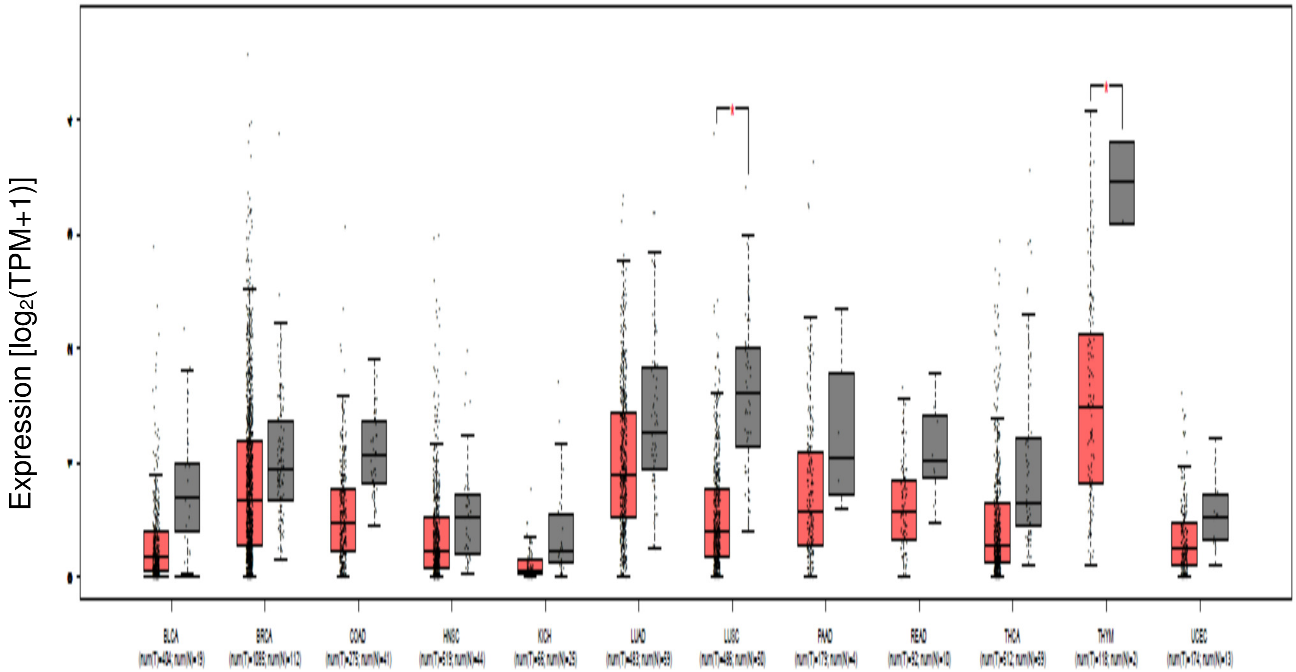

Figure S6. The expressions of the 28 genes from no/low expression groups in cancer cell lines were down-regulated in the tumor samples compared to the normal tissues across different TCGA cancer types. Expression values were given as log_2(TPM + 1) for log-scale. Red boxes denote tumor samples, black boxes denote normal samples.

27) CD27 (TNFRSF7)

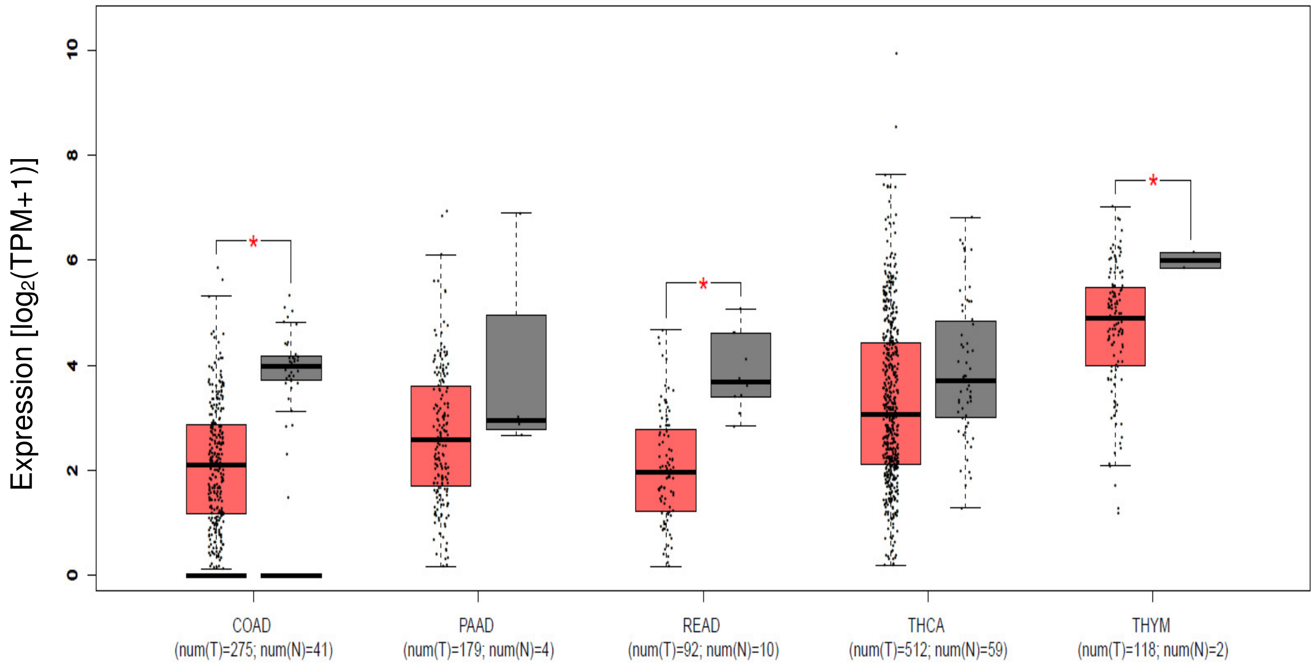

28) CD70 (TNFSF7)

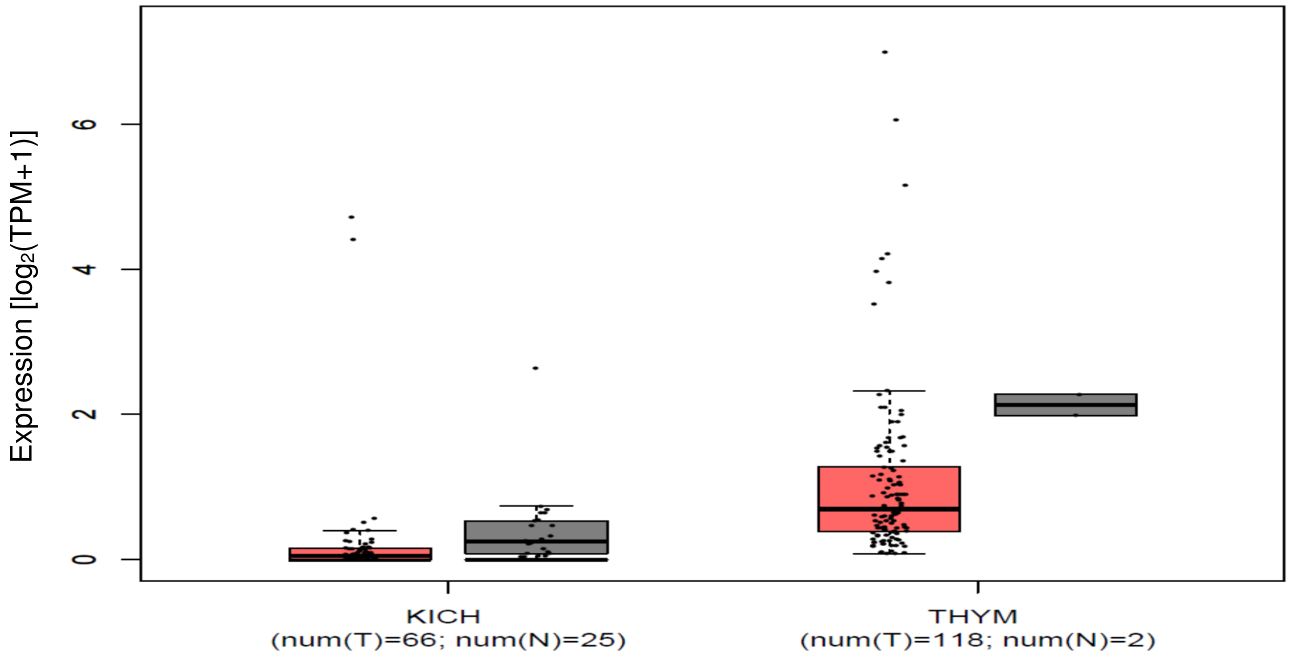

Figure S6. The expressions of the 28 genes from no/low expression groups in cancer cell lines were down-regulated in the tumor samples compared to the normal tissues across different TCGA cancer types. Expression values were given as $\log_2(TPM + 1)$ for log-scale. Red boxes denote tumor samples, black boxes denote normal samples.

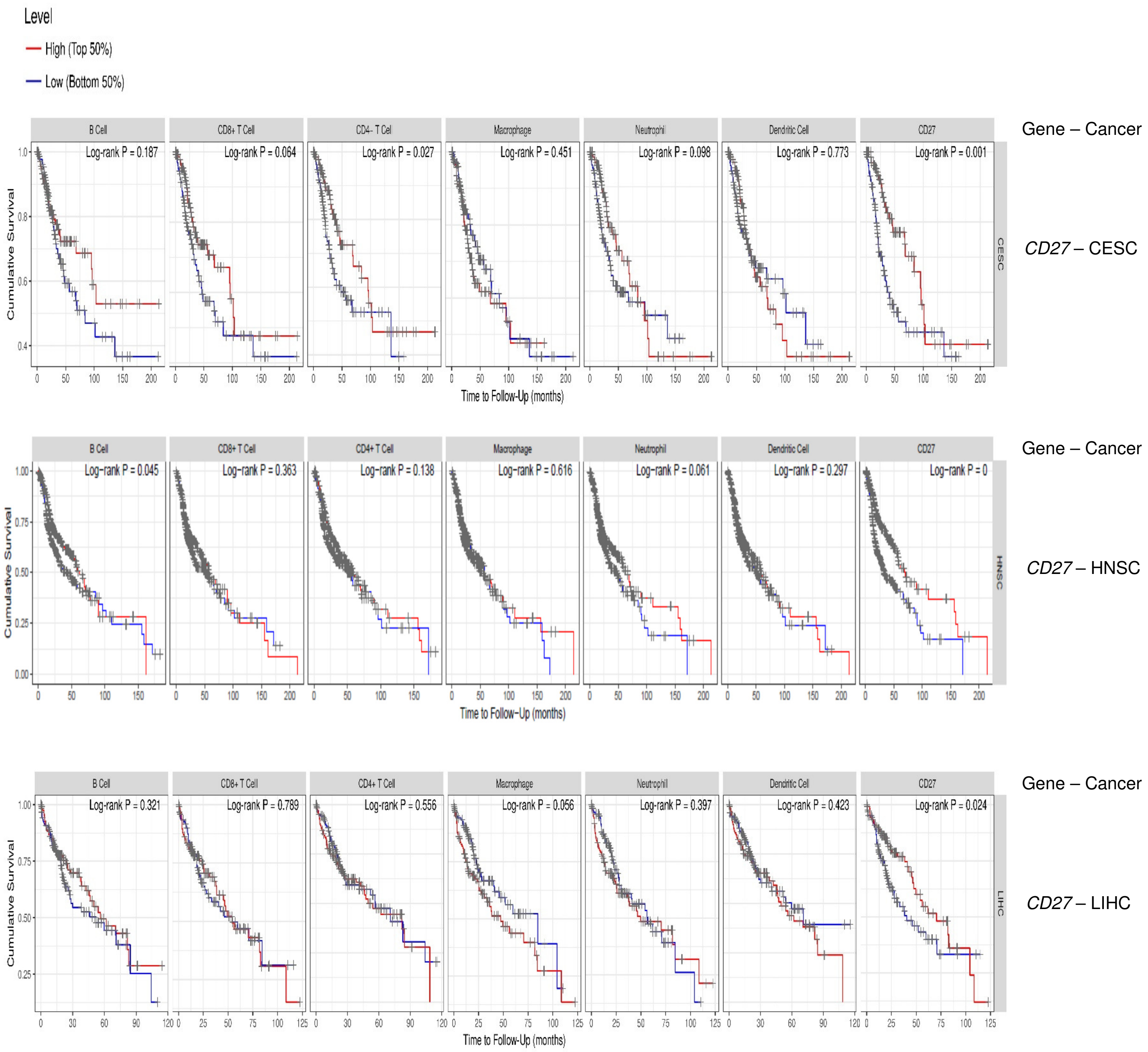

Figure S7. Kaplan–Meier survival curves of the six immune related genes for the overall survival analyses for different TCGA cancer types. It can be seen that the expression of each of the six genes *CD27*, *CEACAM1*, *CTLA4*, *LRIG1*, *PD-L2*, *TNFRSF18* served as the prognostic biomarker that independently predicted the better overall survival outcome after the correction for the abundance of tumor infiltrating immune cells of B cells, CD8+ T cells, CD4+ T cells, Macrophages, Neutrphils, and Dendritic cells.

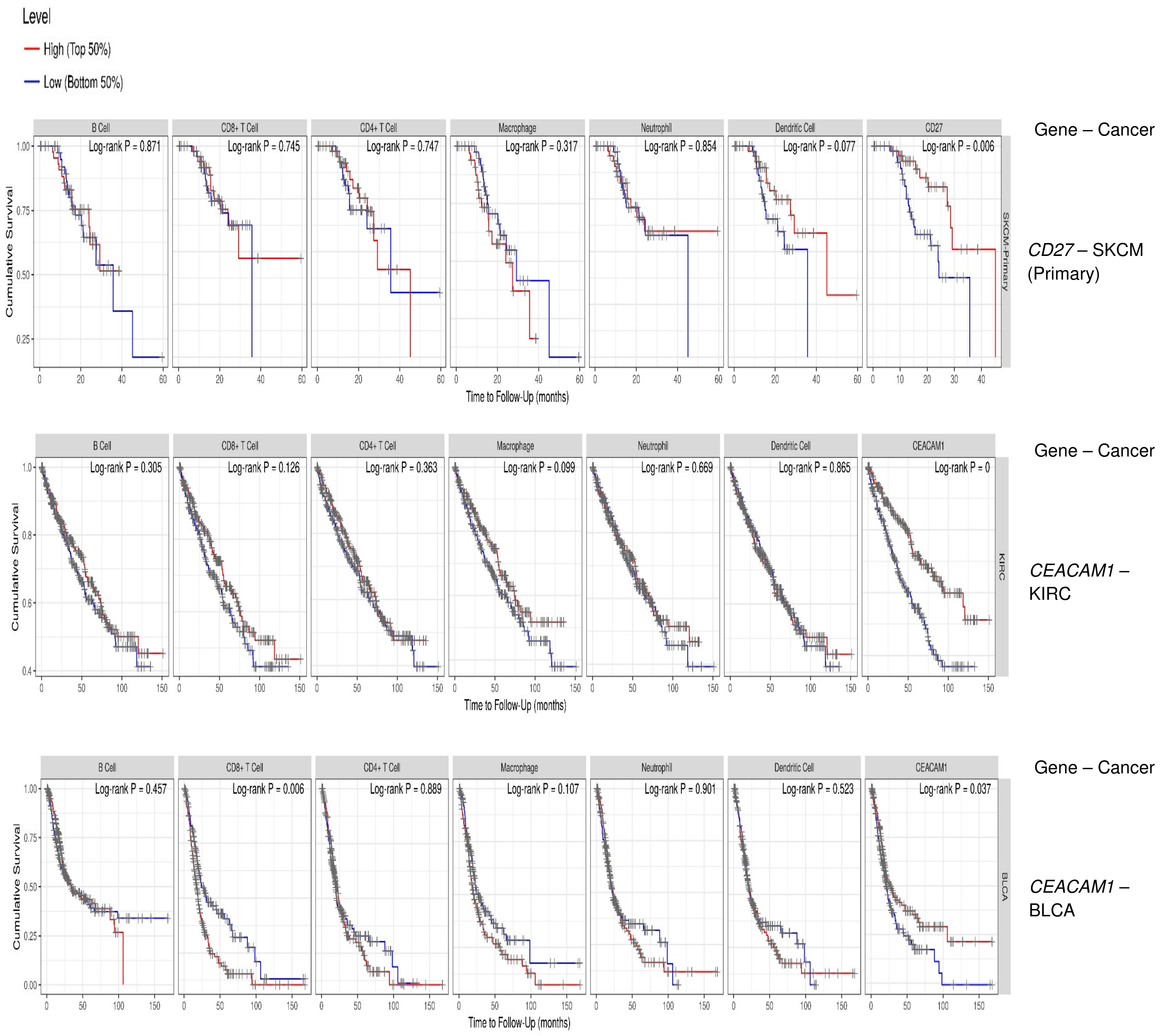

Figure S7. Kaplan–Meier survival curves of the six immune related genes for the overall survival analyses for different TCGA cancer types. It can be seen that the expression of each of the six genes *CD27*, *CEACAM1*, *CTLA4*, *LRIG1*, *PD-L2*, *TNFRSF18* served as the prognostic biomarker that independently predicted the better overall survival outcome after the correction for the abundance of tumor infiltrating immune cells of B cells, CD8+ T cells, CD4+ T cells, Macrophages, Neutrphils, and Dendritic cells.

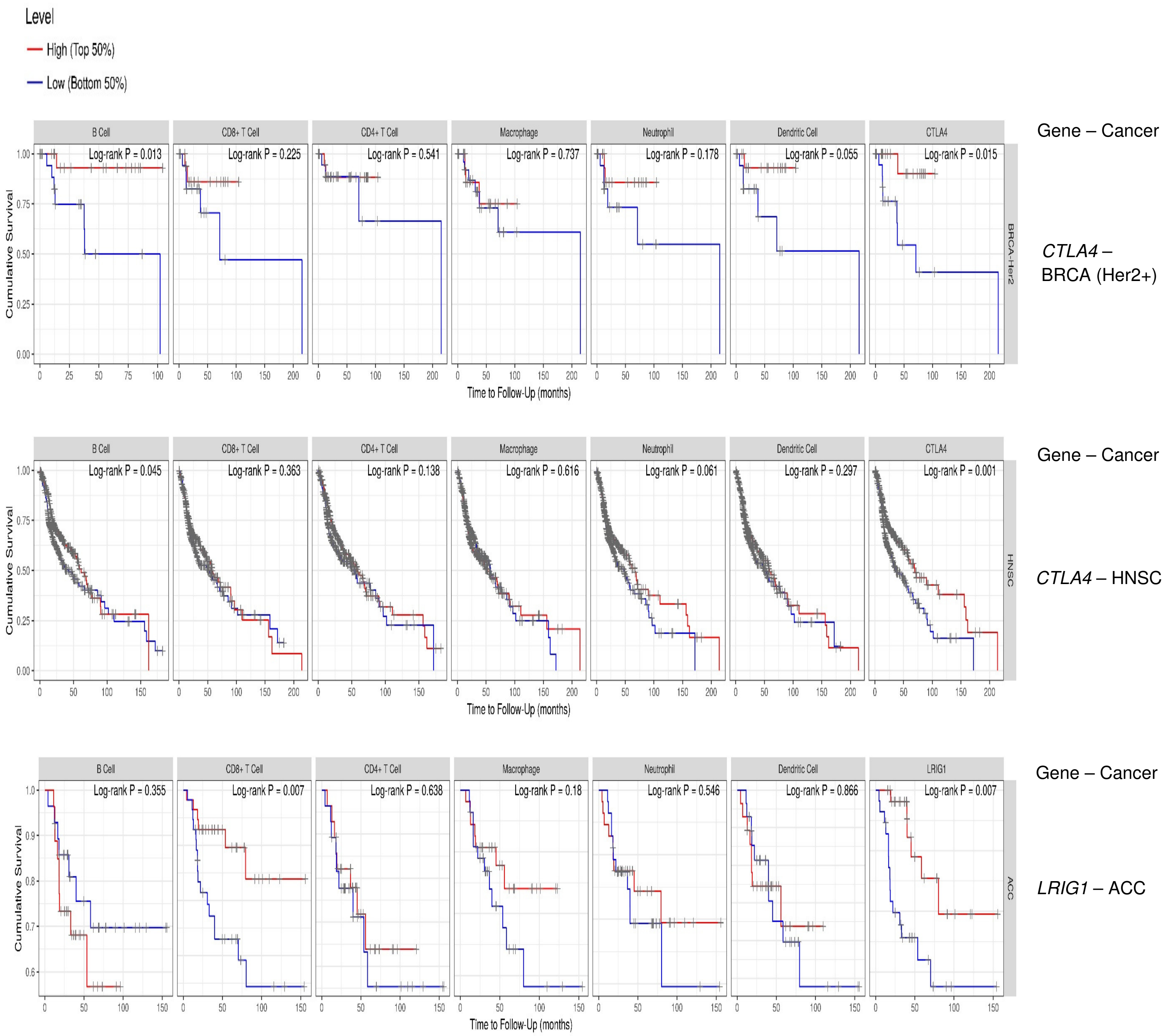

Figure S7. Kaplan–Meier survival curves of the six immune related genes for the overall survival analyses for different TCGA cancer types. It can be seen that the expression of each of the six genes *CD27*, *CEACAM1*, *CTLA4*, *LRIG1*, *PD-L2*, *TNFRSF18* served as the prognostic biomarker that independently predicted the better overall survival outcome after the correction for the abundance of tumor infiltrating immune cells of B cells, CD8+ T cells, CD4+ T cells, Macrophages, Neutrphils, and Dendritic cells.

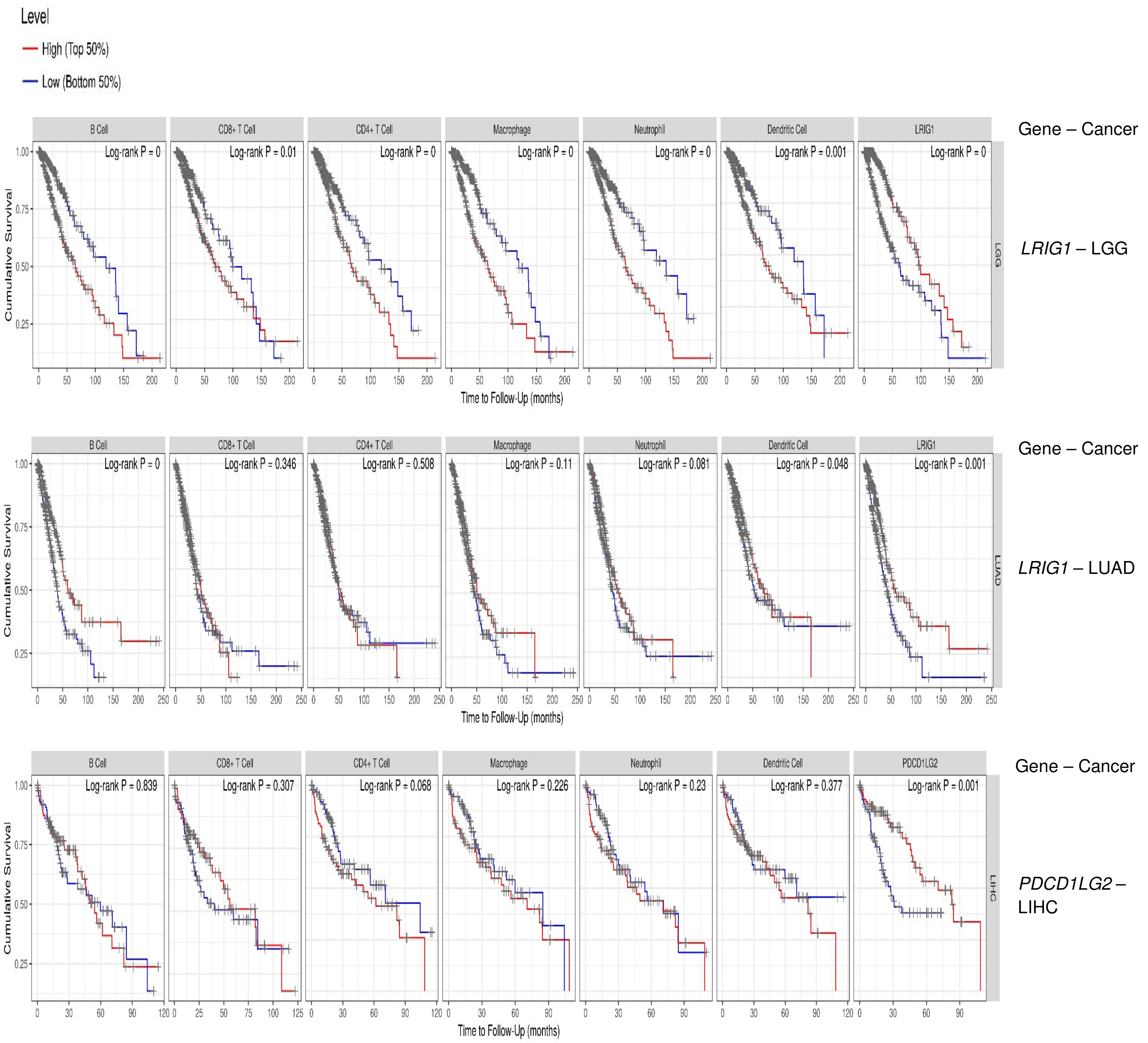

Figure S7. Kaplan–Meier survival curves of the six immune related genes for the overall survival analyses for different TCGA cancer types. It can be seen that the expression of each of the six genes *CD27*, *CEACAM1*, *CTLA4*, *LRIG1*, *PD-L2*, *TNFRSF18* served as the prognostic biomarker that independently predicted the better overall survival outcome after the correction for the abundance of tumor infiltrating immune cells of B cells, CD8+ T cells, CD4+ T cells, Macrophages, Neutrphils, and Dendritic cells.

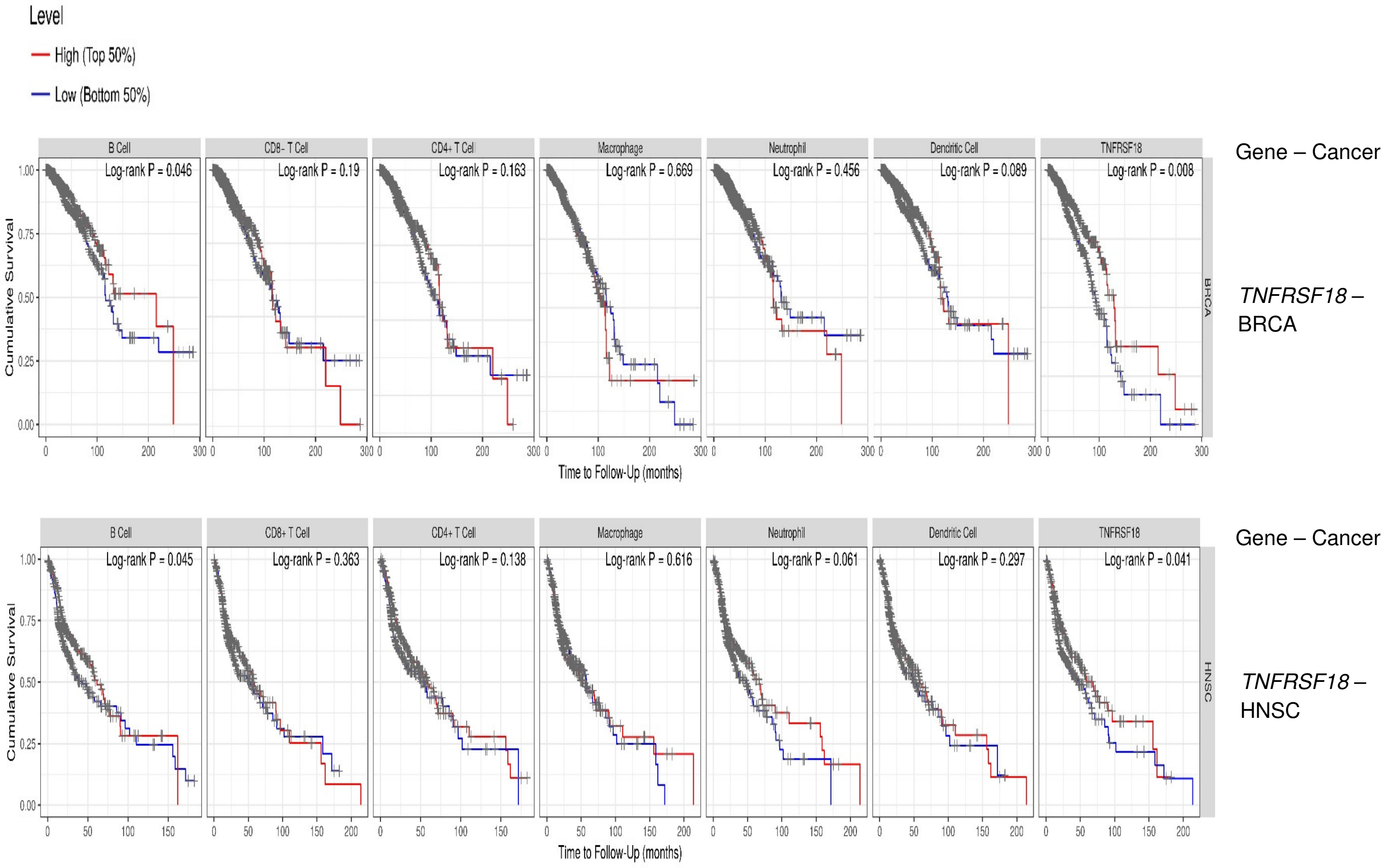

Figure S7. Kaplan–Meier survival curves of the six immune related genes for the overall survival analyses for different TCGA cancer types. It can be seen that the expression of each of the six genes *CD27*, *CEACAM1*, *CTLA4*, *LRIG1*, *PD-L2*, *TNFRSF18* served as the prognostic biomarker that independently predicted the better overall survival outcome after the correction for the abundance of tumor infiltrating immune cells of B cells, CD8+ T cells, CD4+ T cells, Macrophages, Neutrphils, and Dendritic cells.

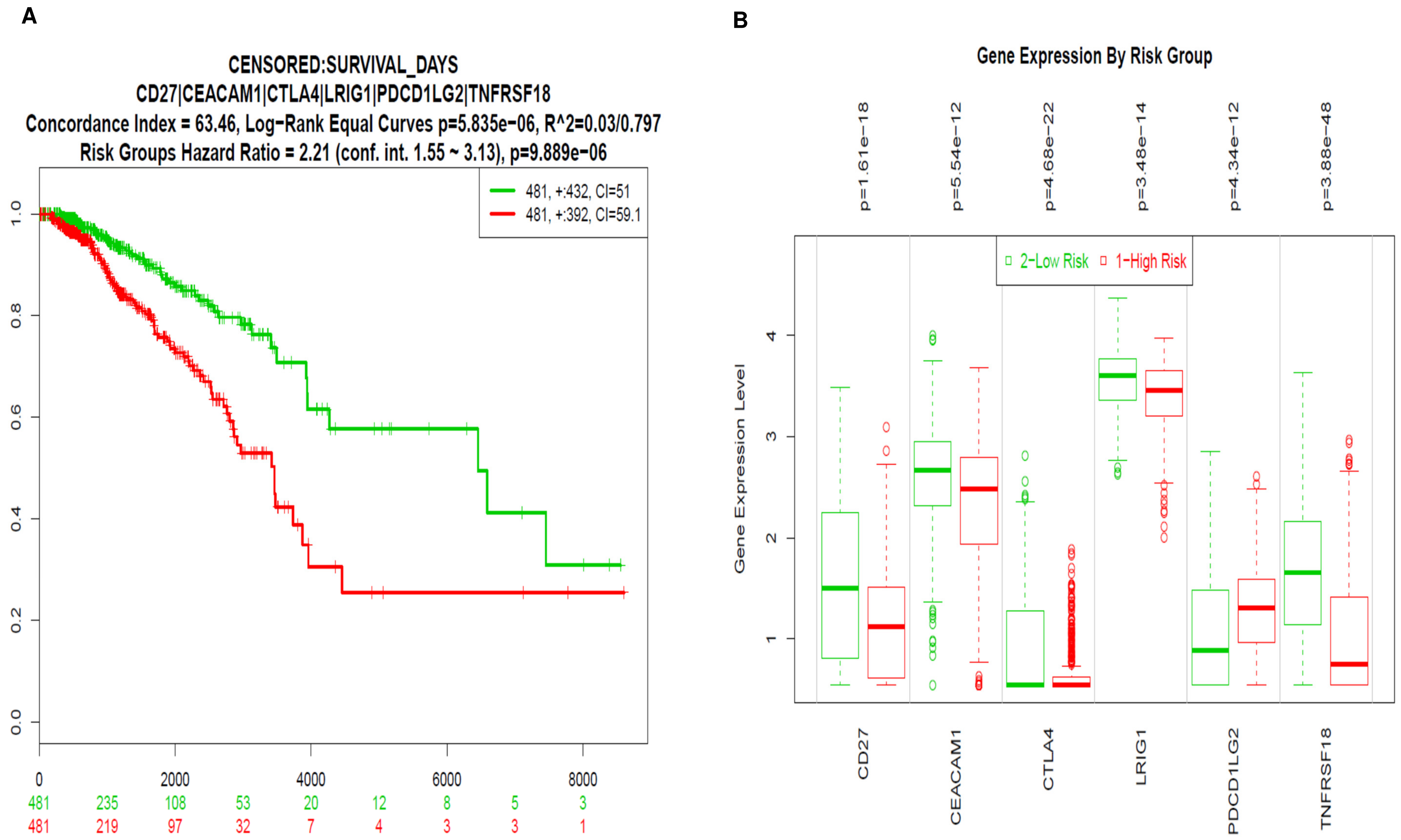

Figure S8. High and low risk groups of overall survival in patients were characterized by low and high expression of Immu6Metagene signature. KM plots and corresponding expression levels of the six genes for the high and low risk groups were shown for (A)-(B): BRCA (Breast invasive carcinoma); (C)-(D): CESC (Cervical and endocervical cancers); (E)-(F): LUAD (Lung adenocarcinoma).

C

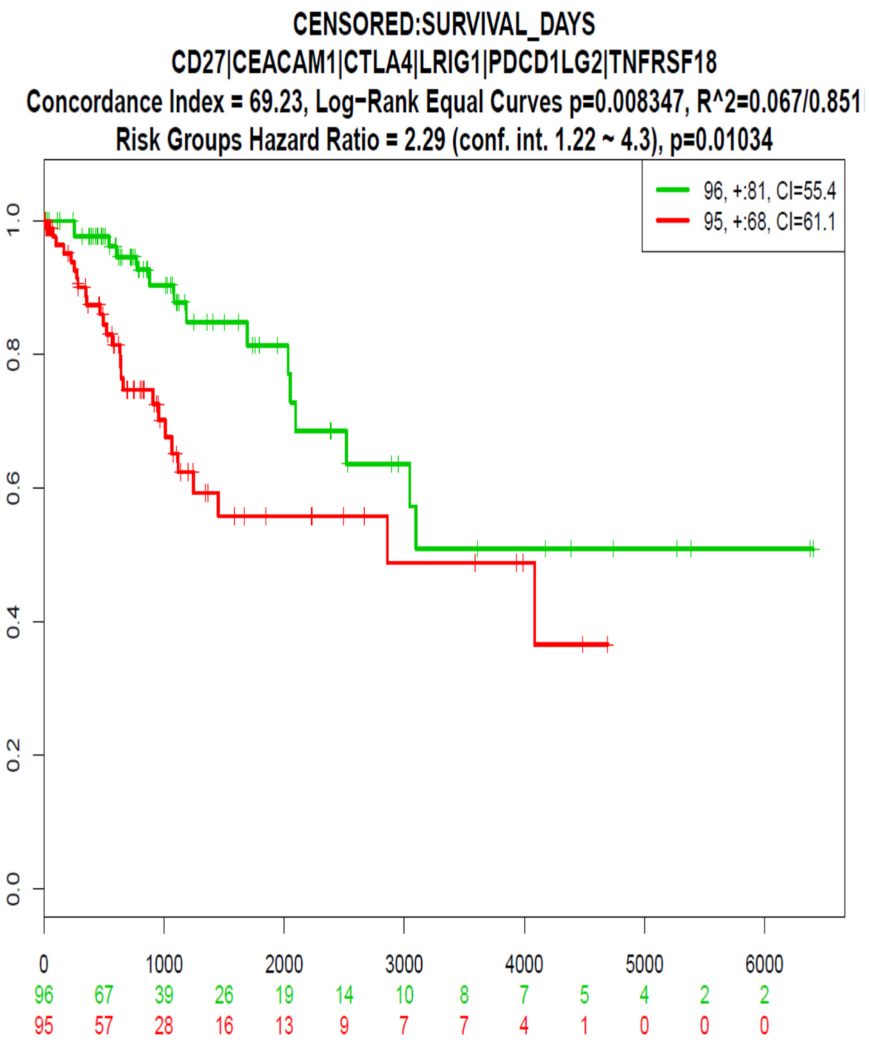

D

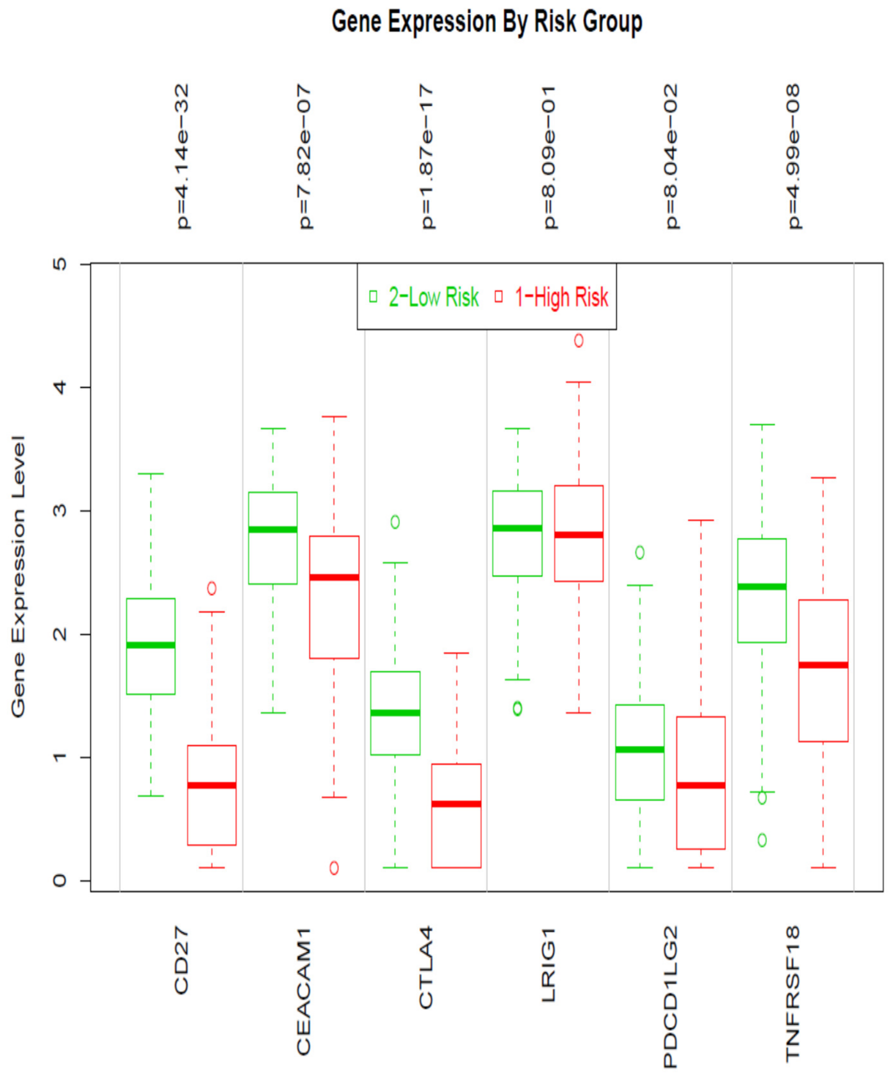

Figure S8. High and low risk groups of overall survival in patients were characterized by low and high expression of Immu6Metagene signature. KM plots and corresponding expression levels of the six genes for the high and low risk groups were shown for (A)-(B): BRCA (Breast invasive carcinoma); (C)-(D): CESC (Cervical and endocervical cancers); (E)-(F): LUAD (Lung adenocarcinoma).

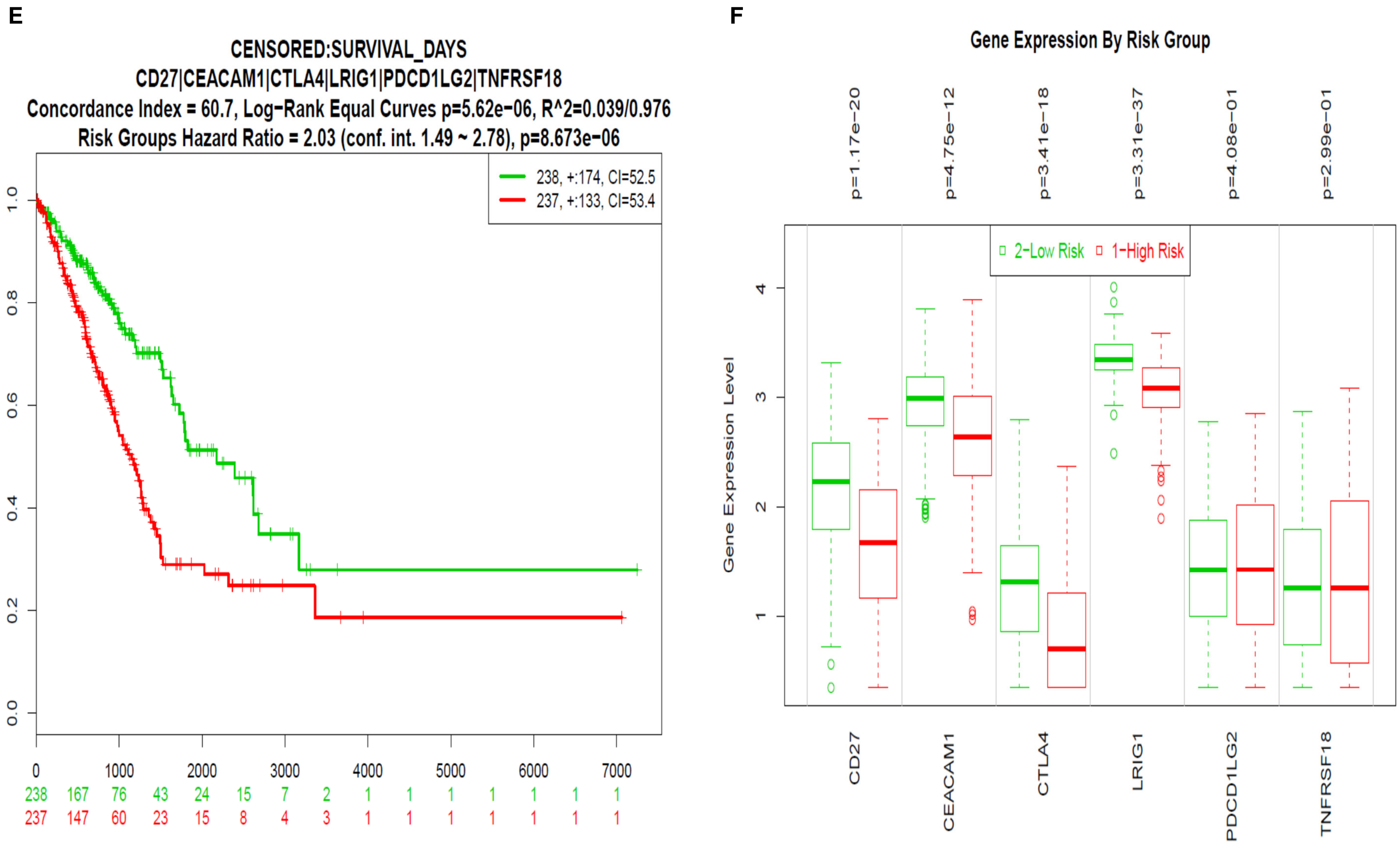

Figure S8. High and low risk groups of overall survival in patients were characterized by low and high expression of Immu6Metagene signature. KM plots and corresponding expression levels of the six genes for the high and low risk groups were shown for (A)-(B): BRCA (Breast invasive carcinoma); (C)-(D): CESC (Cervical and endocervical cancers); (E)-(F): LUAD (Lung adenocarcinoma).

A. *CD27* methylation in the BRCA cohort

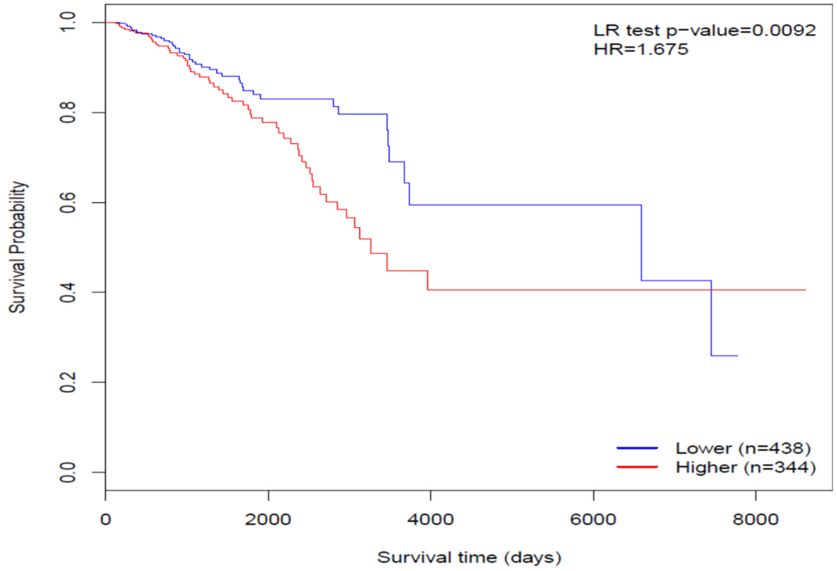

B. *CD27* methylation in the CESC cohort

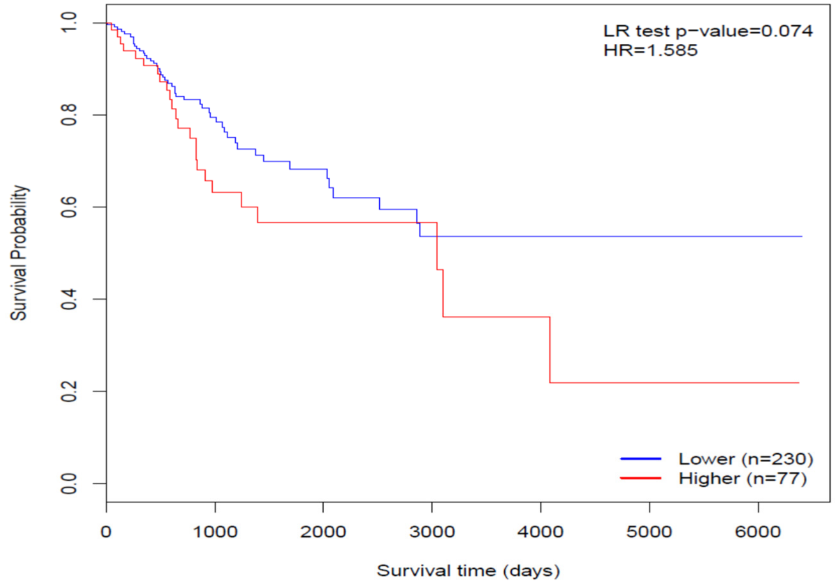

C. *CD27* methylation in the LUAD cohort

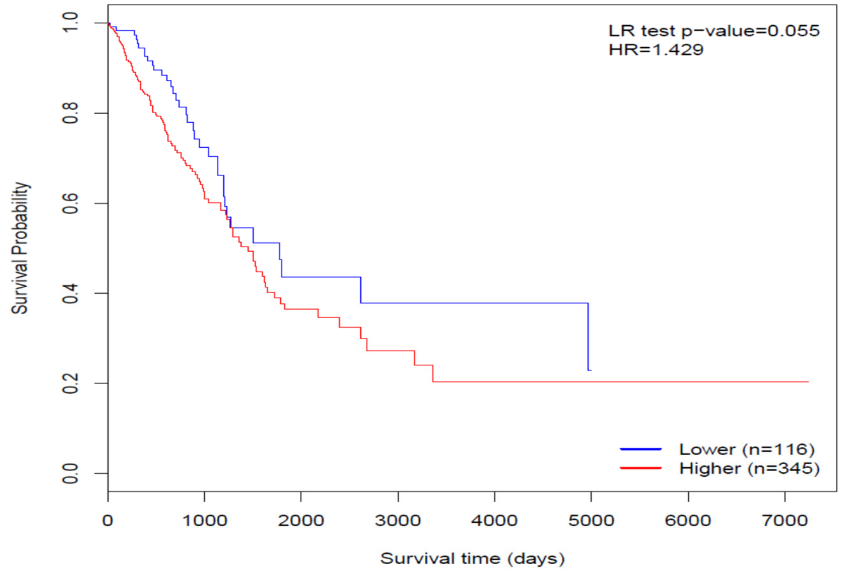

D. *CEACAM1* methylation in the BRCA cohort

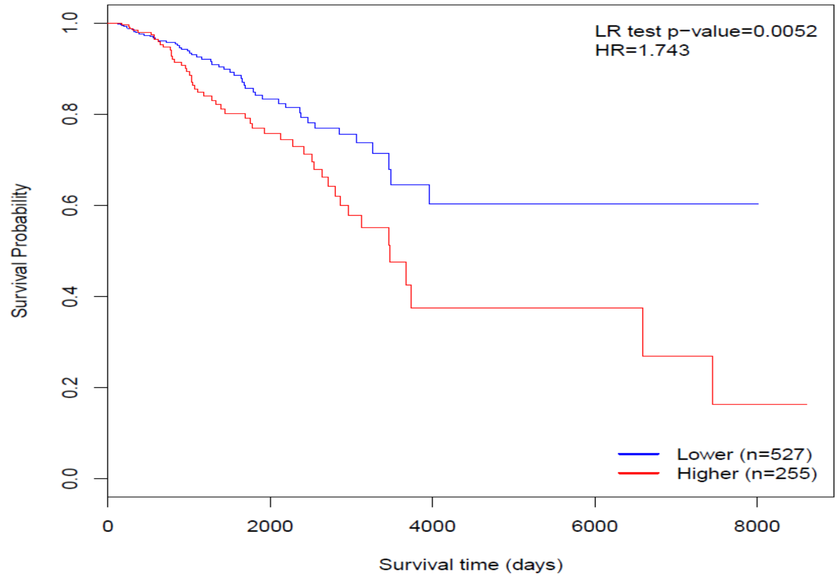

Figure S9. DNA methylation levels of the six genes of the Immu6Metagene signature and their associations with overall survival in the TCGA cohorts. Higher methylation levels of these six genes were significantly associated with worse overall survival outcomes in the TCGA cancer cohorts of BRCA (A, D, F, I, L, N), CESC (B, E, G, J, O) and LUAD (C, H, K, M, P).

E. *CEACAM1* methylation in the CESC cohort

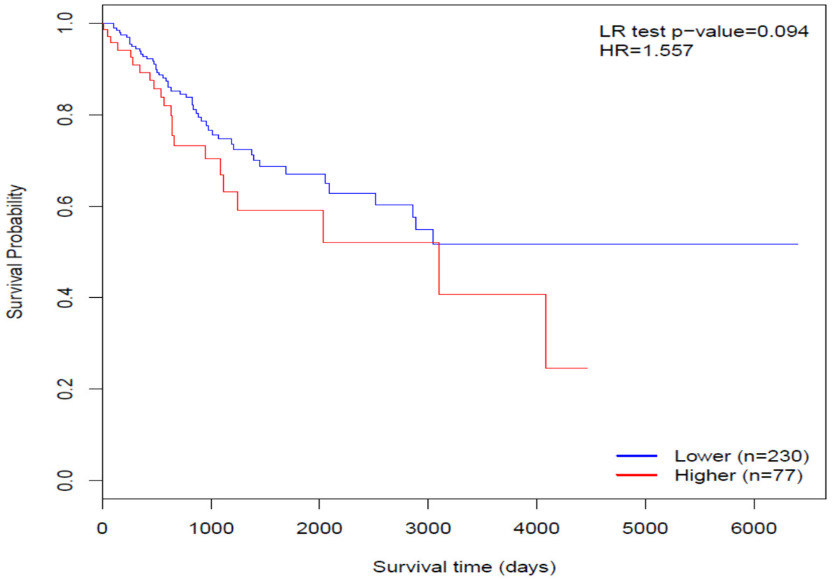

F. *CTLA4* methylation in the BRCA cohort

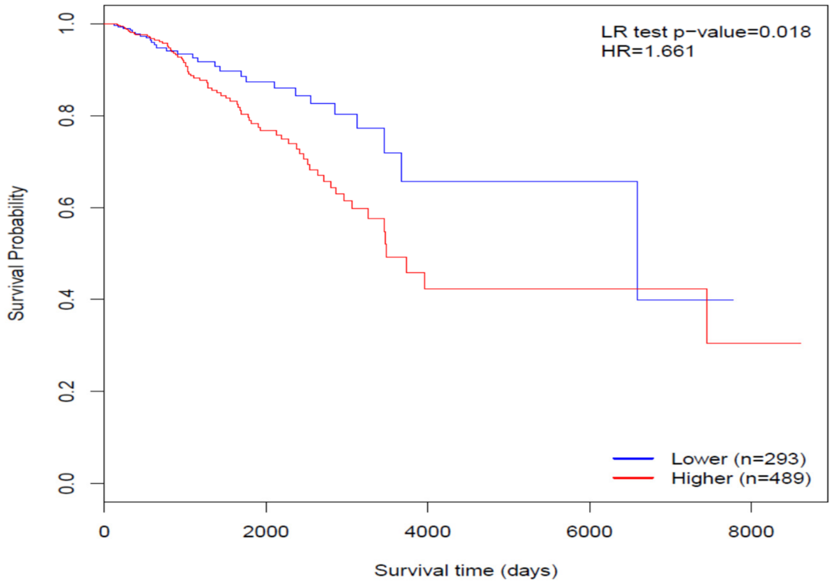

G. *CTLA4* methylation in the CESC cohort

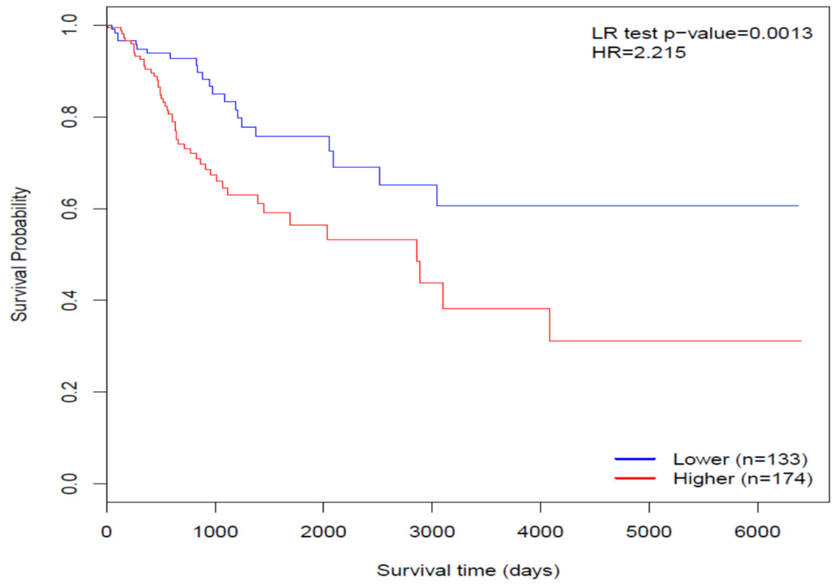

H. *CTLA4* methylation in the LUAD cohort

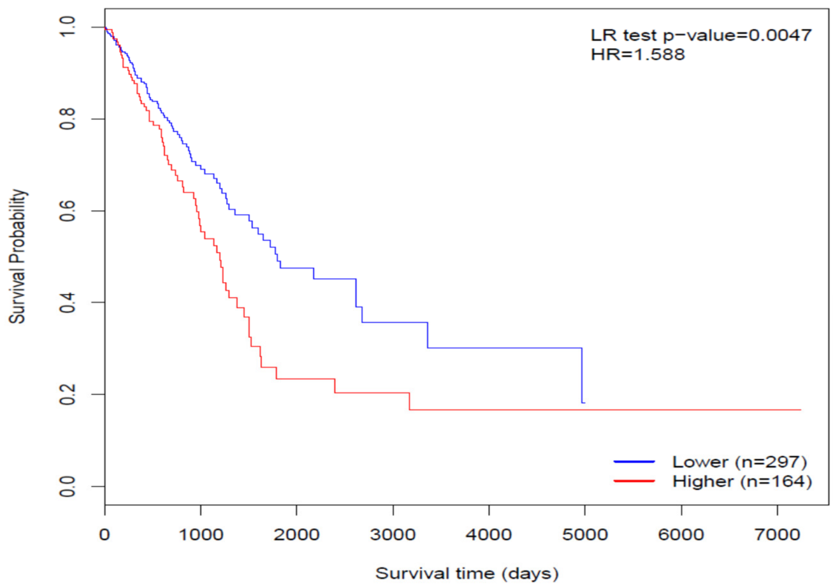

Figure S9. DNA methylation levels of the six genes of the Immu6Metagene signature and their associations with overall survival in the TCGA cohorts. Higher methylation levels of these six genes were significantly associated with worse overall survival outcomes in the TCGA cancer cohorts of BRCA (A, D, F, I, L, N), CESC (B, E, G, J, O) and LUAD (C, H, K, M, P).

I. *LRIG1* methylation in the BRCA cohort

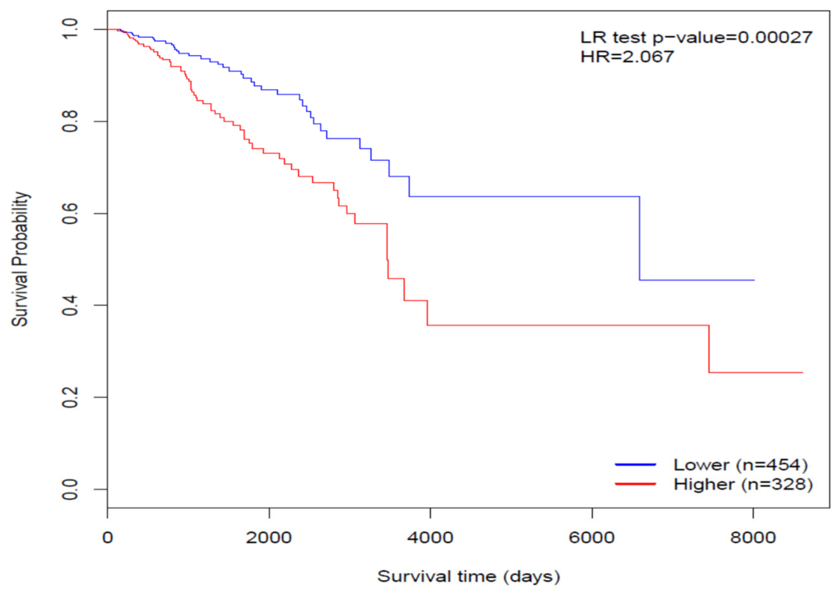

J. *LRIG1* methylation in the CESC cohort

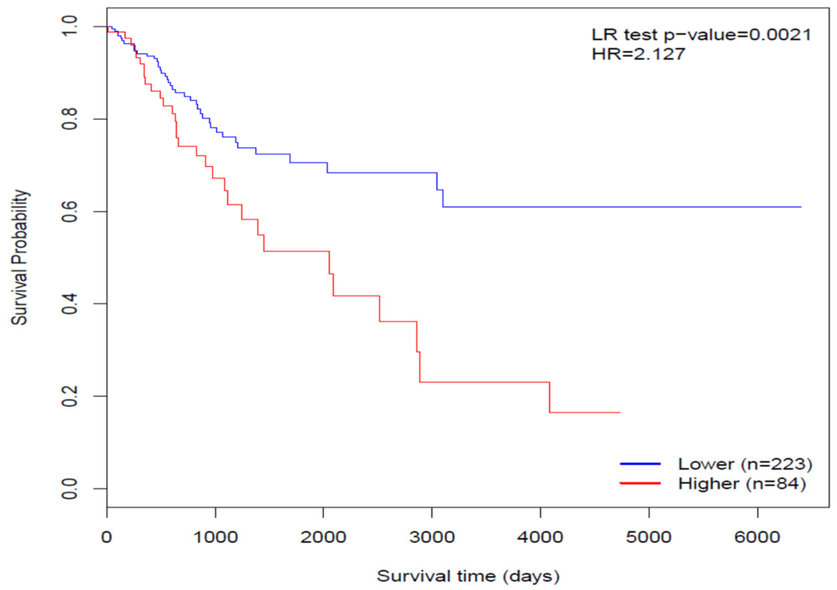

K. *LRIG1* methylation in the LUAD cohort

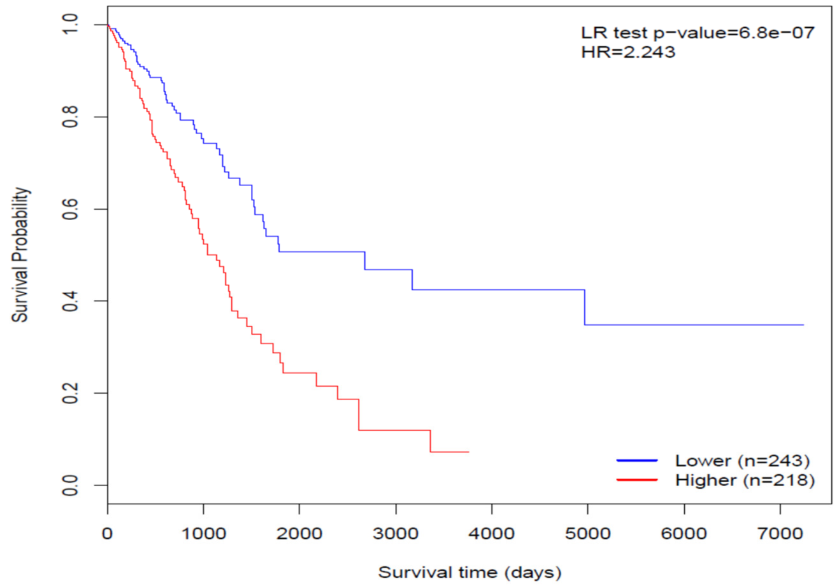

L. *PDCD1LG2* methylation in the BRCA cohort

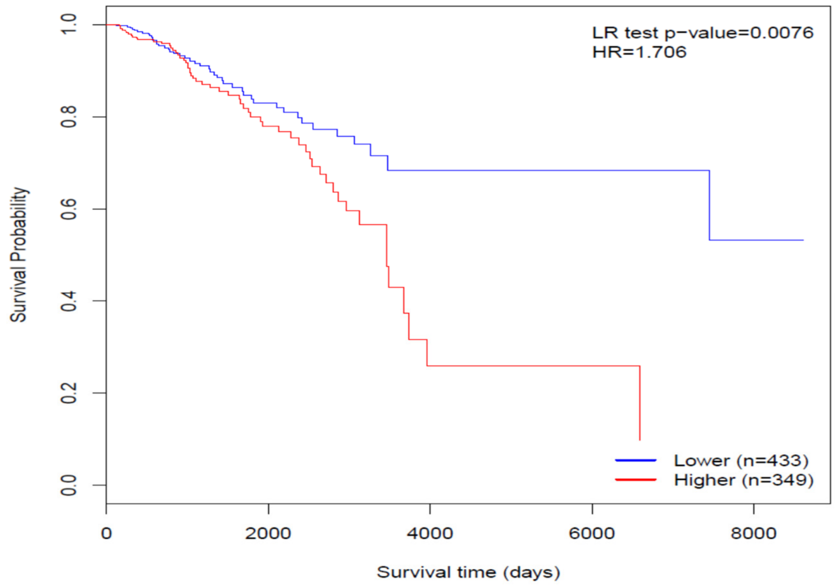

Figure S9. DNA methylation levels of the six genes of the Immu6Metagene signature and their associations with overall survival in the TCGA cohorts. Higher methylation levels of these six genes were significantly associated with worse overall survival outcomes in the TCGA cancer cohorts of BRCA (A, D, F, I, L, N), CESC (B, E, G, J, O) and LUAD (C, H, K, M, P).

M. *PDCD1LG2* methylation in the LUAD cohort

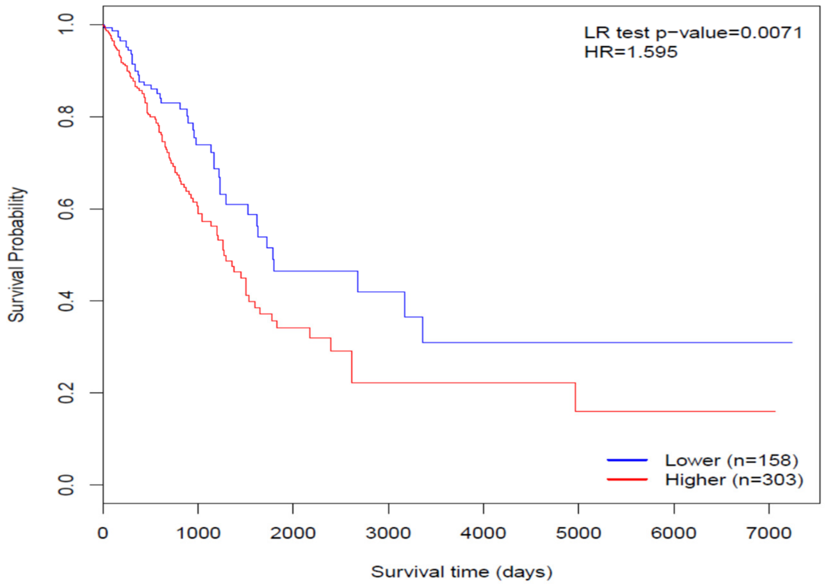

N. *TNFRSF18* methylation in the BRCA cohort

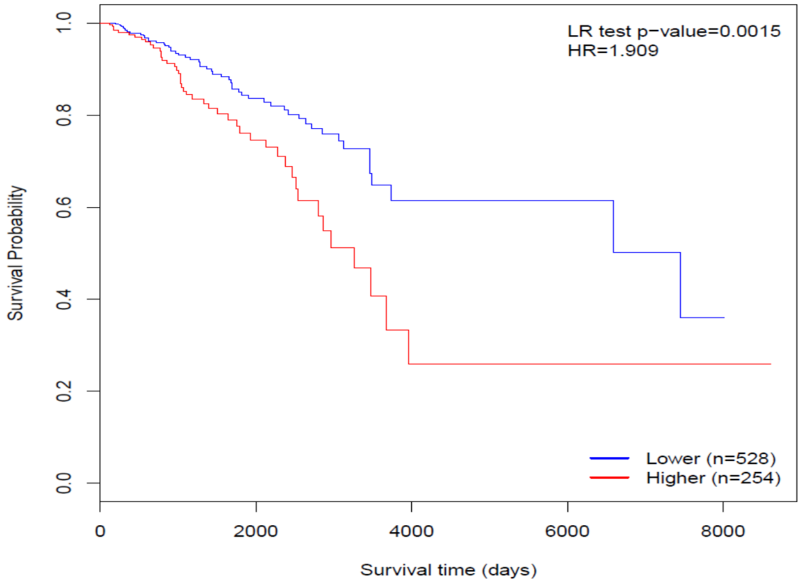

O. *TNFRSF18* methylation in the CESC cohort

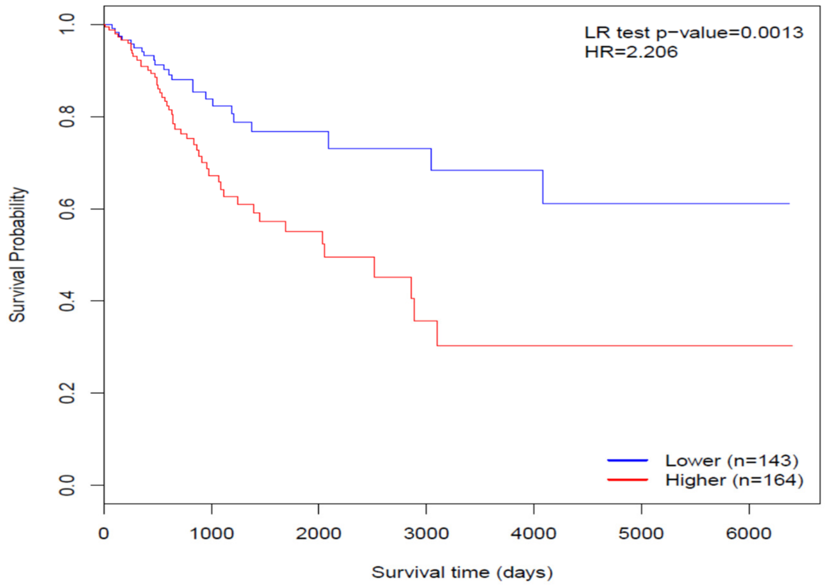

P. *TNFRSF18* methylation in the LUAD cohort

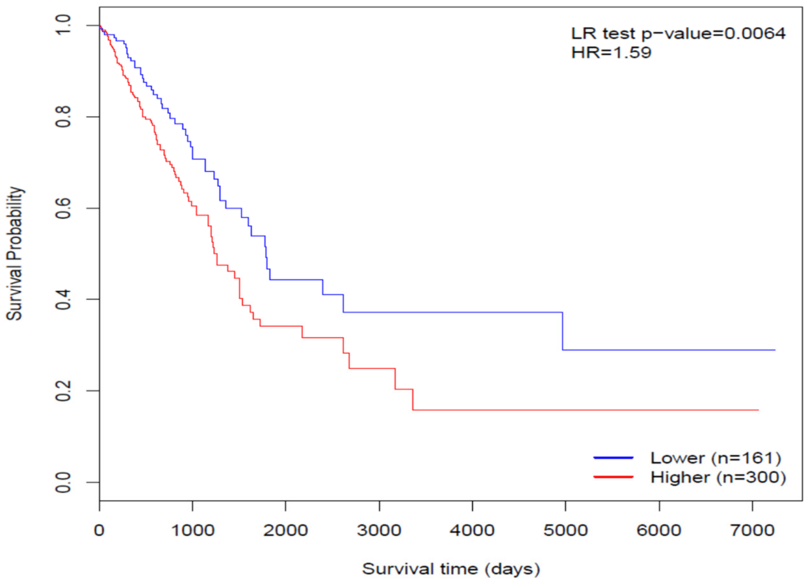

Figure S9. DNA methylation levels of the six genes of the Immu6Metagene signature and their associations with overall survival in the TCGA cohorts. Higher methylation levels of these six genes were significantly associated with worse overall survival outcomes in the TCGA cancer cohorts of BRCA (A, D, F, I, L, N), CESC (B, E, G, J, O) and LUAD (C, H, K, M, P).

A. *CD27*

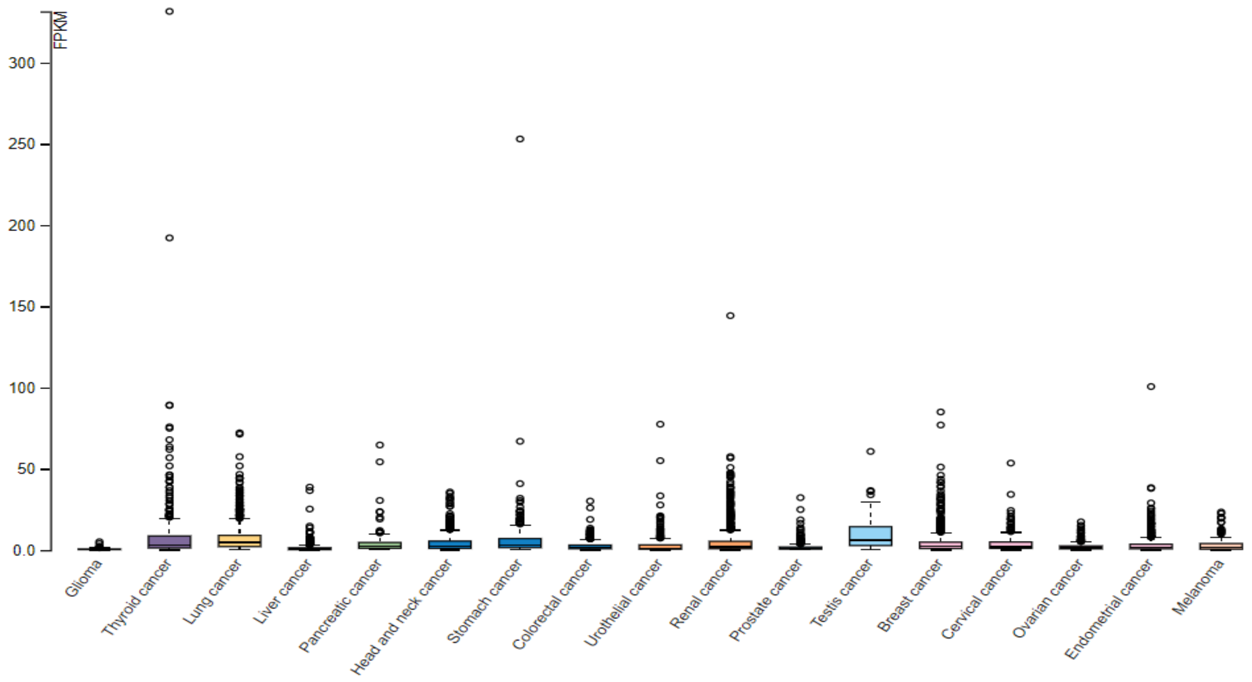

B. *CEACAM1*

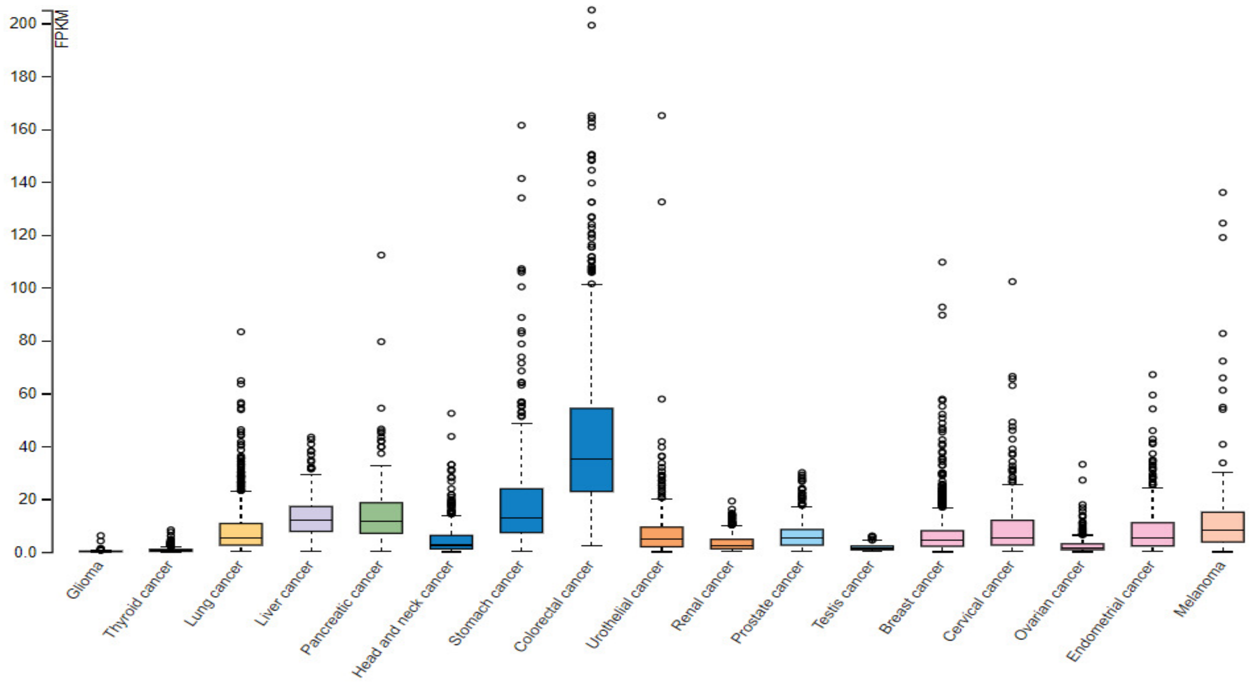

Figure S10. RNA expression overview of the genes of the Immu6Metagene signature. Box plots of the mRNA expression levels across the TCGA cancer types were given for the six genes – (A) *CD27*, (B) *CEACAM1*, (C) *CTLA4*, (D) *LRIG1*, (E) *PDCD1LG2* (*PD-L2*), (F) *TNFRSF18*.

C. *CTLA4*

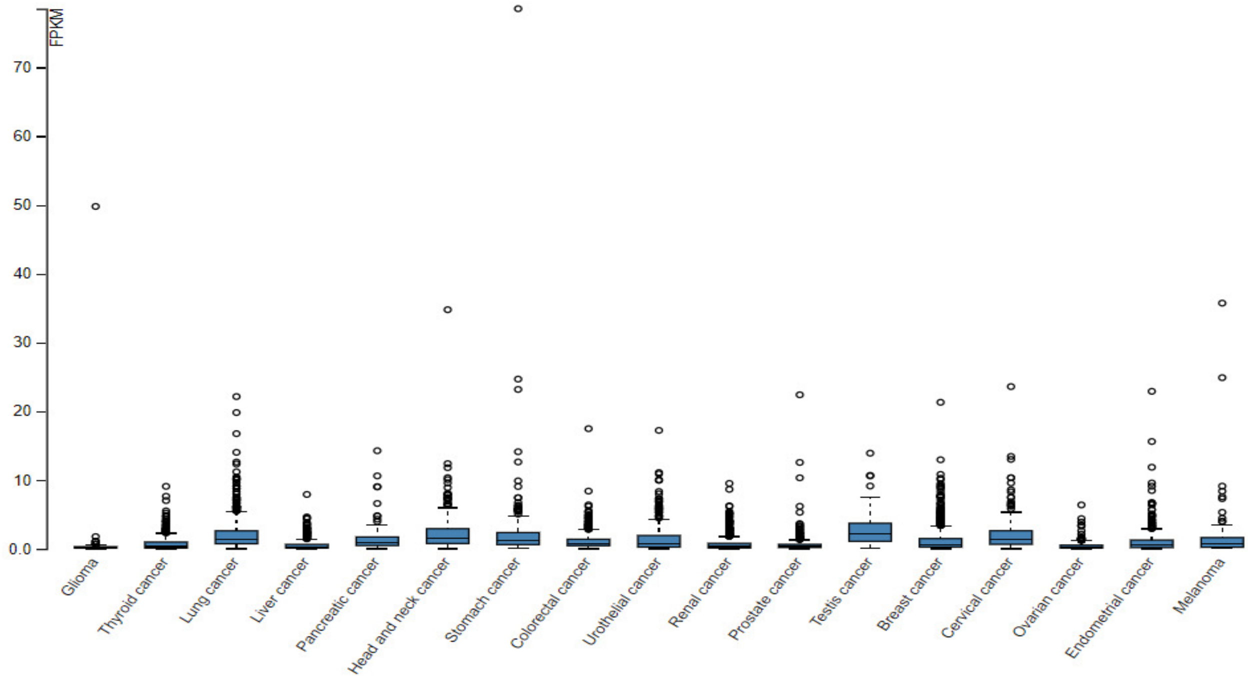

D. *LRIG1*

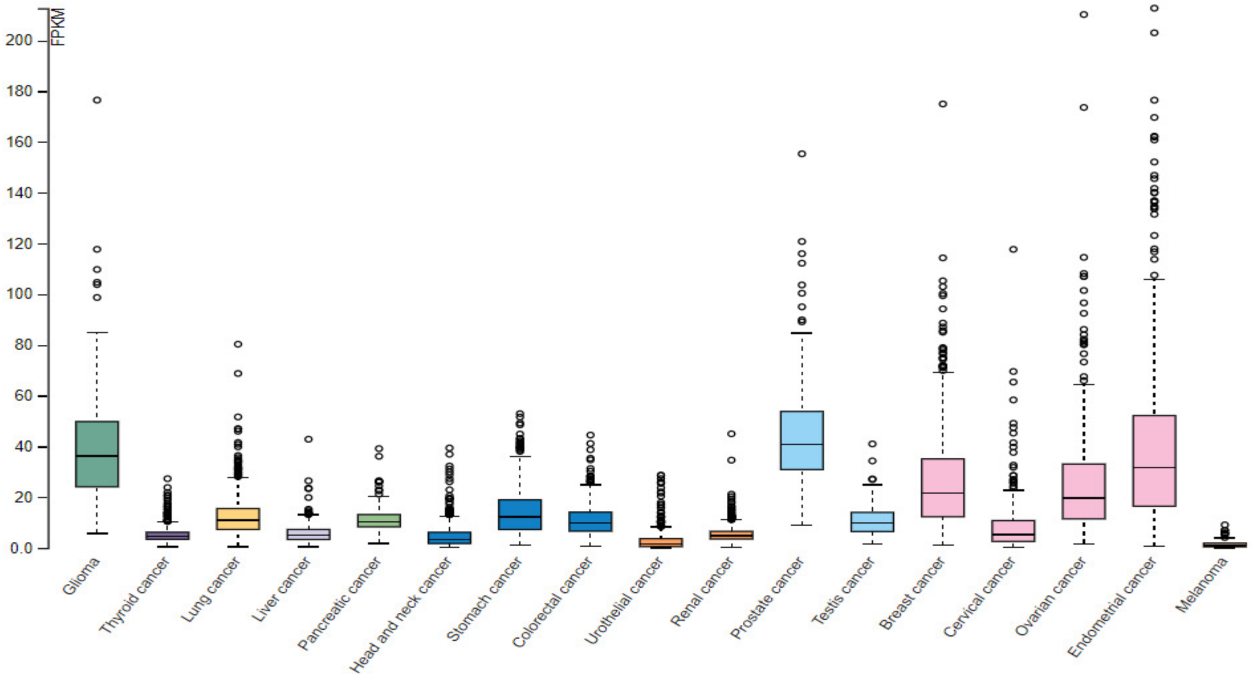

Figure S10 (continuing). RNA expression overview of the genes of the Immu6Metagene signature. Box plots of the mRNA expression levels across the TCGA cancer types were given for the six genes – (A) *CD27*, (B) *CEACAM1*, (C) *CTLA4*, (D) *LRIG1*, (E) *PDCD1LG2* (*PD-L2*), (F) *TNFRSF18*.

E. *PDCD1LG2*

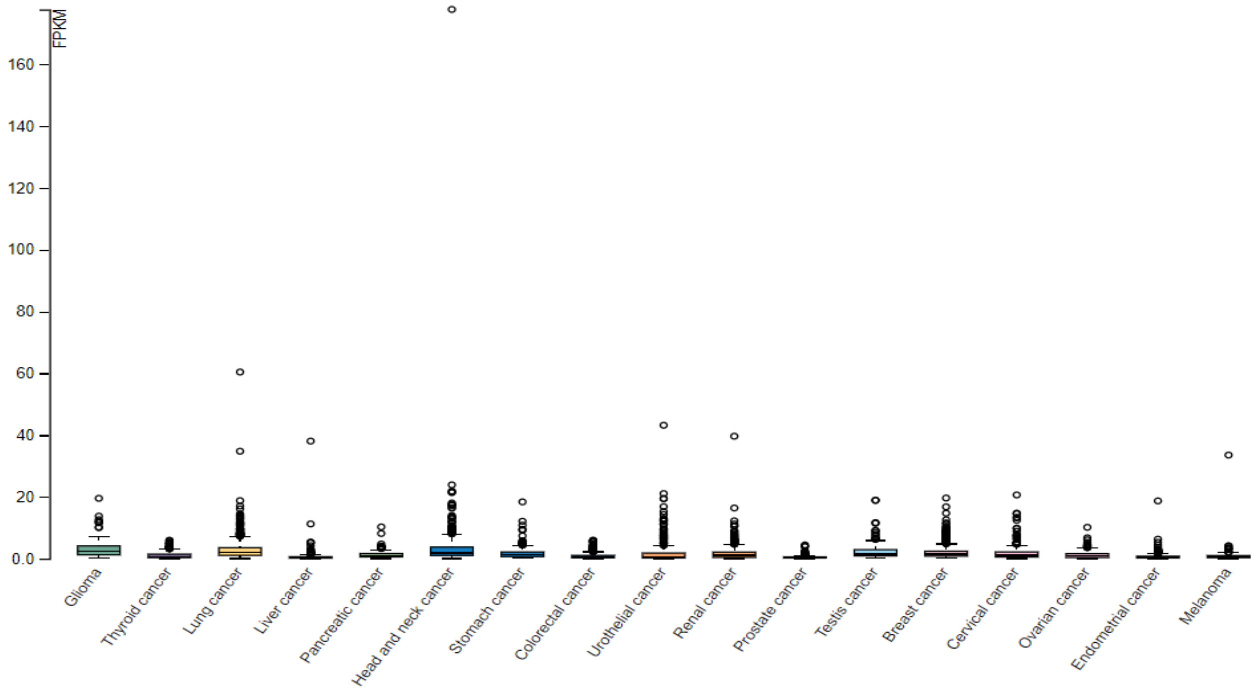

F. *TNFRSF18*

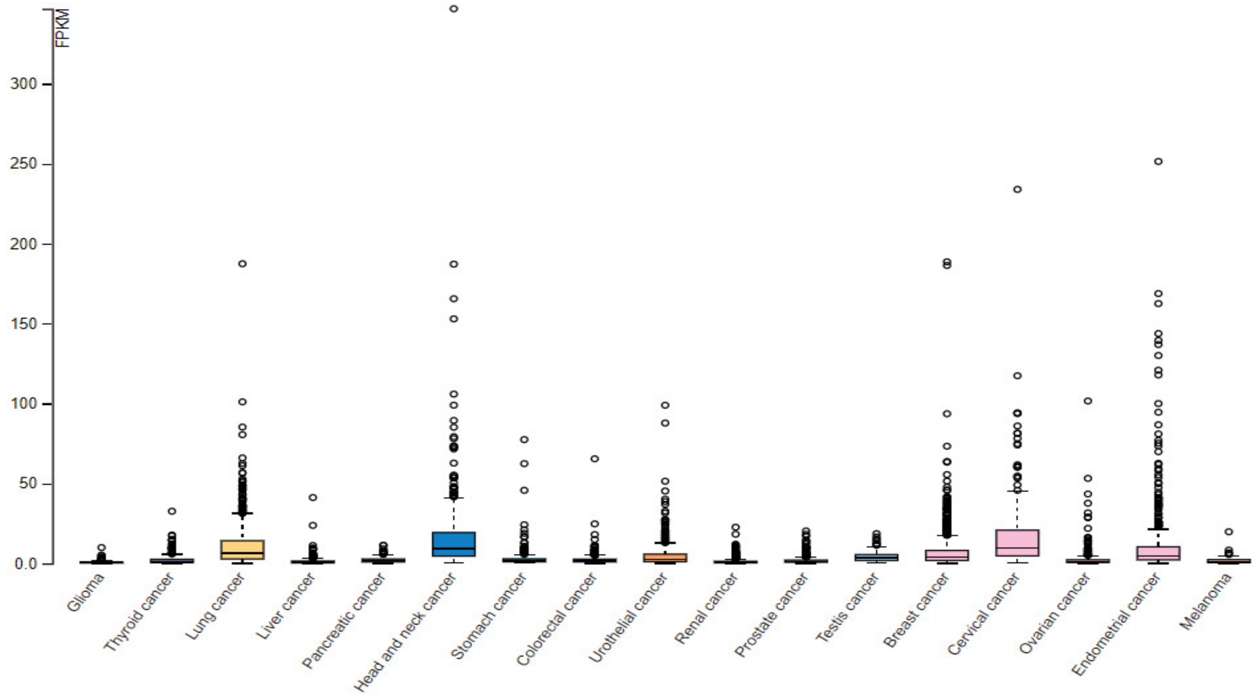

Figure S10 (continuing). RNA expression overview of the genes of the Immu6Metagene signature. Box plots of the mRNA expression levels across the TCGA cancer types were given for the six genes – (A) *CD27*, (B) *CEACAM1*, (C) *CTLA4*, (D) *LRIG1*, (E) *PDCD1LG2* (*PD-L2*), (F) *TNFRSF18*.

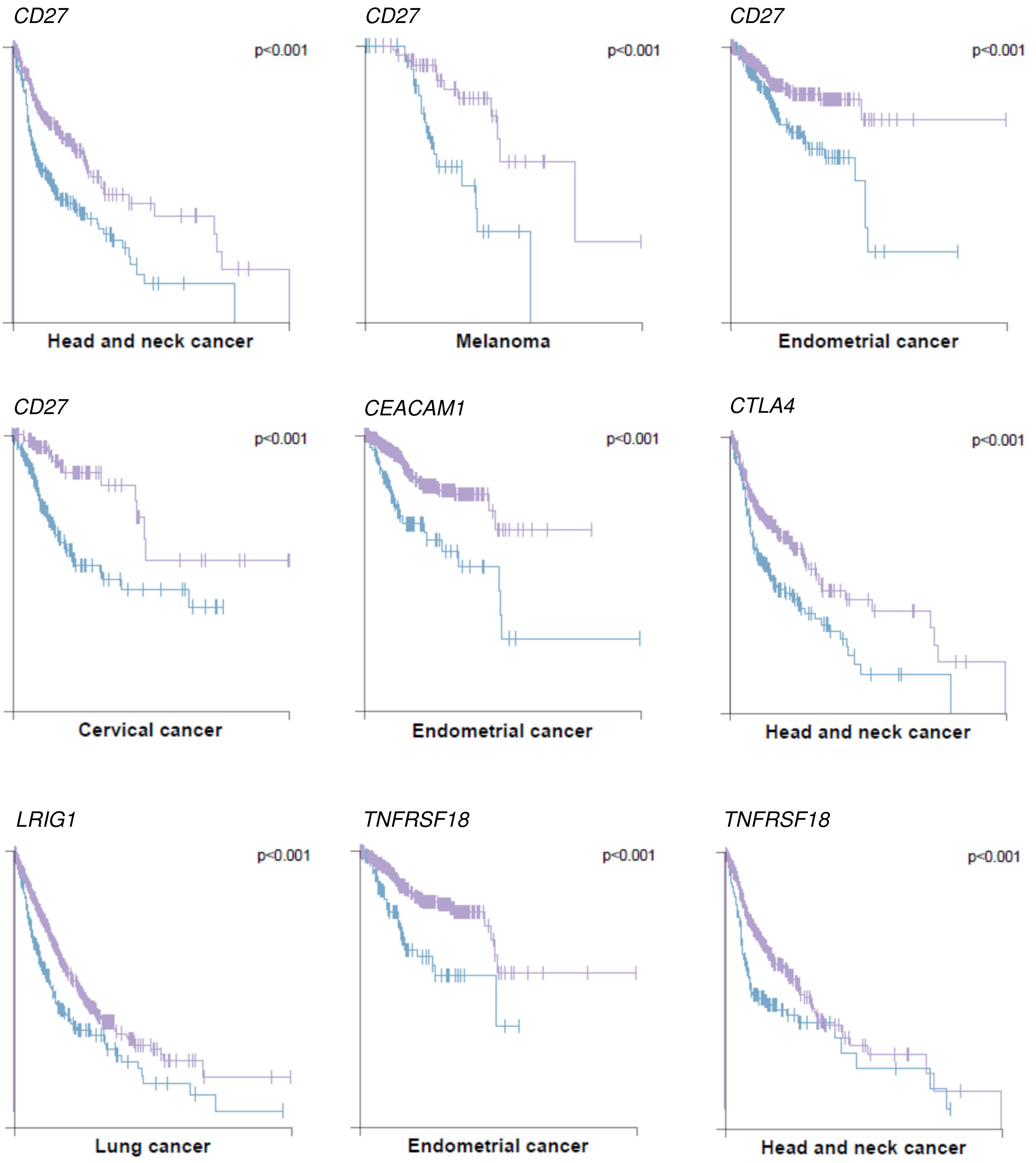

Figure S11. The low expressions of the genes – *CD27*, *CEACAM1*, *CTLA4*, *LRIG1*, *TNFRSF18* of the Immu6Metagene signature significantly associated with the shorter overall survival of the patients of different cancer types. The Kaplan–Meier survival curves were obtained from THE HUMAN PROTEIN ATLAS database. The patients have been stratified into high (purple lines) or low (blue lines) expression group of each gene. The log rank P values were shown in the plots.

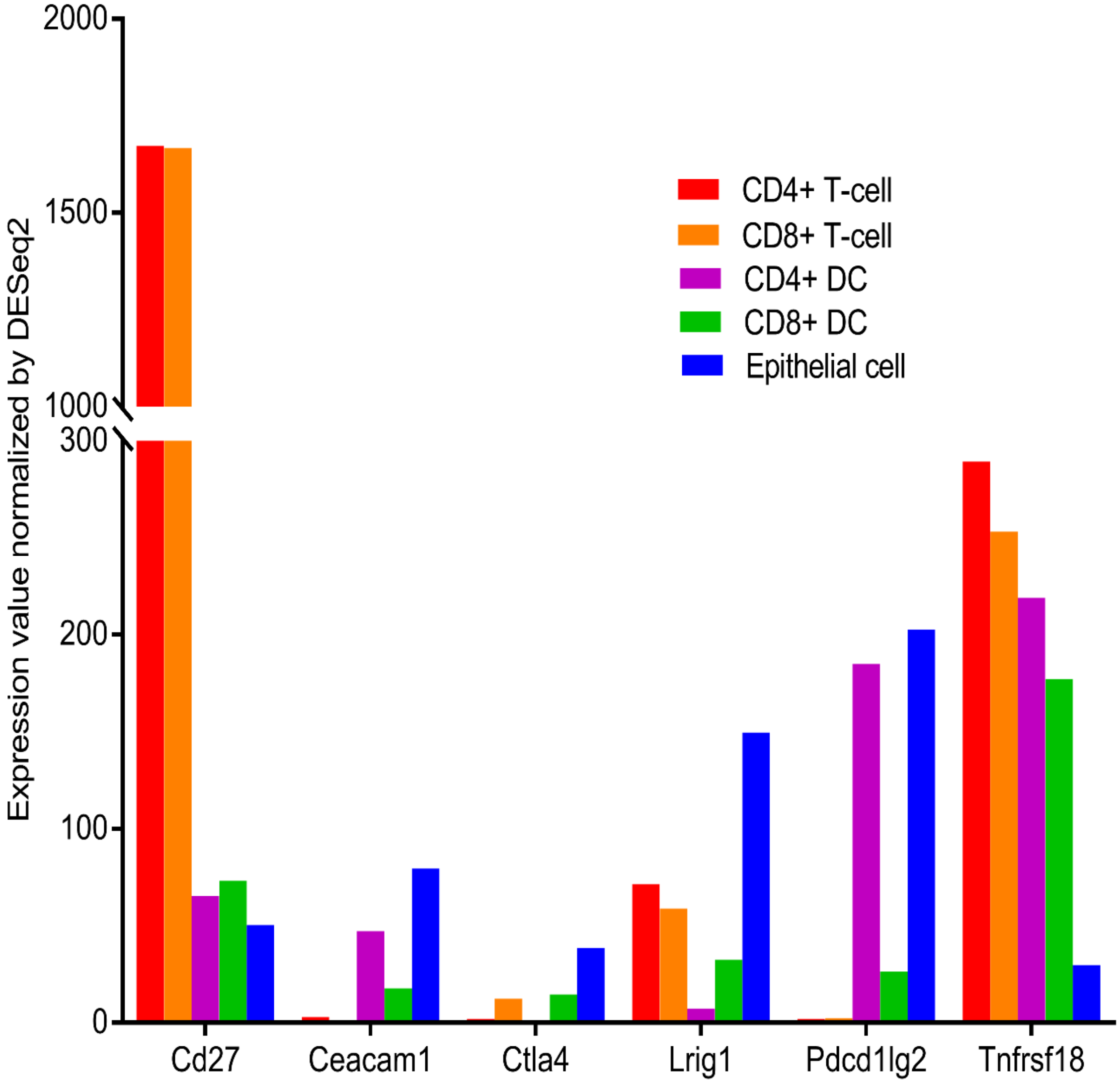

Figure S12. The expression of the six genes of the Immu6Metagene signature across the CD4+ or CD8+ T cells, CD4+ or CD8+ dendritic cells (DCs) and normal epithelial cells. The analysis was performed for the mouse data downloaded from the 'Gene Skyline' browser (http://rstats.immgen.org/Skyline/skyline.html) of the Immunological Genome project. The gene expression values were normalized by using the DESeq2 software.
